# Template-Directed Synthesis of Recognition-Encoded Melamine Oligomers Using a Base-Filling Strategy

**DOI:** 10.1021/jacs.5c05681

**Published:** 2025-05-15

**Authors:** Joseph T. Smith, Joaquin Baixeras Buye, Ben Iddon, Daniil O. Soloviev, Christopher A. Hunter

**Affiliations:** Yusuf Hamied Department of Chemistry, 2152 University of Cambridge, Cambridge CB2 1EW, U.K.

## Abstract

Replication of molecular information in nature is based on the synthesis of the backbone of the copy strand by polymerization of monomers bound to a template. An alternative strategy is to use a preassembled polymer backbone devoid of sequence information as the copy strand and to attach side chains in a sequence determined by binding to a template, i.e., base-filling. Base-filling strategies were investigated for template-directed synthesis of recognition-encoded melamine oligomers (REMO) using H-bond base-pairing interactions between 4-nitrophenol and phosphine oxide side chains. A template with three 4-nitrophenol H-bond donor recognition units was used with a blank copy strand equipped with three aldehyde groups for the reversible attachment of amine recognition units via dynamic imine chemistry. Equilibration of the template and blank strands in dichloromethane in the presence of benzylamine and a phosphine oxide recognition unit equipped with an amine resulted in selective incorporation (79%) of the phosphine oxide recognition unit into the resulting copy strand. Covalent attachment of the blank strand to the template with a diester linker increased the selectivity of the base-filling process to 85%, and carrying out the experiment in toluene further increased the selectivity to 92%. The imines in the copy strand were trapped by reduction, and cleavage of the ester linkages allowed recovery of the template strand along with the kinetically stable tris-phosphine oxide copy. Fidelity of templating is determined by the concentration of the template strand, the association constant for the base-pairing interaction, and the effective molarities of the intramolecular interactions in the duplex.

## Graphical abstract

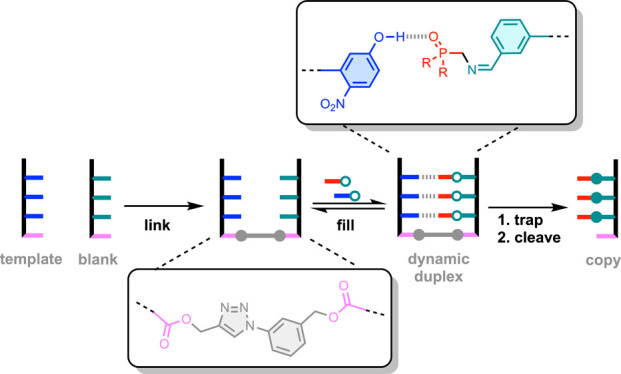

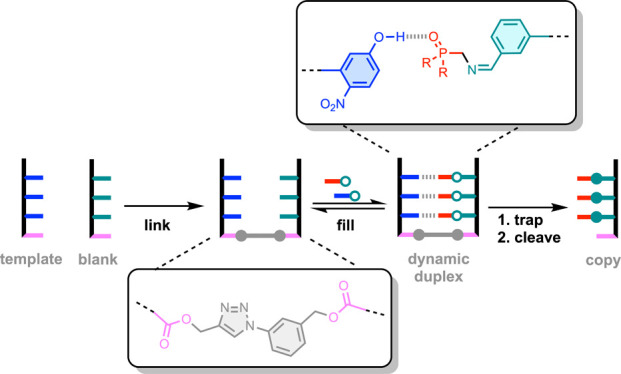

## Introduction

The template-directed synthesis of nucleic acids by enzymatic processes allows the replication of genetic information and hence evolution.^1^ Functional nucleic acids with new properties have been obtained by directed evolution using SELEX processes.^2–4^ By expanding directed evolution to synthetic polymers, which have quite different chemical properties, new chemical space could be explored and new functional polymers obtained. To achieve this goal, the template-directed synthesis of polymers must be realized to allow information transfer between generations of oligomers created in an evolutionary cycle.

Previous attempts to replicate sequence information in synthetic oligomers have relied on the templated polymerization of monomers.^5–20^ In these templating processes, monomers are bound to a template to form a pre-ZIP intermediate, then polymerization yields a duplex (Figure 1). Dissociation of the duplex gives the copy strand, which is complementary in sequence to the template. Formation of the duplex during the ZIP reaction requires that monomers bound to the template only react with monomers bound to adjacent sites. The template must therefore organize adjacent monomers in close proximity, and all other monomers must be held further apart to prevent mis-coupling and macrocyclisation reactions. If noncovalent or dynamic base-pairing interactions are used, then off-template binding can lead to competing intramolecular monomer–monomer reactions within the monomer complex. The relative rates at which competing reactions occur depend on the effective molarities (EM) for the different intramolecular processes, which are often similar to one another and difficult to predict.^14^

**Figure 1:**
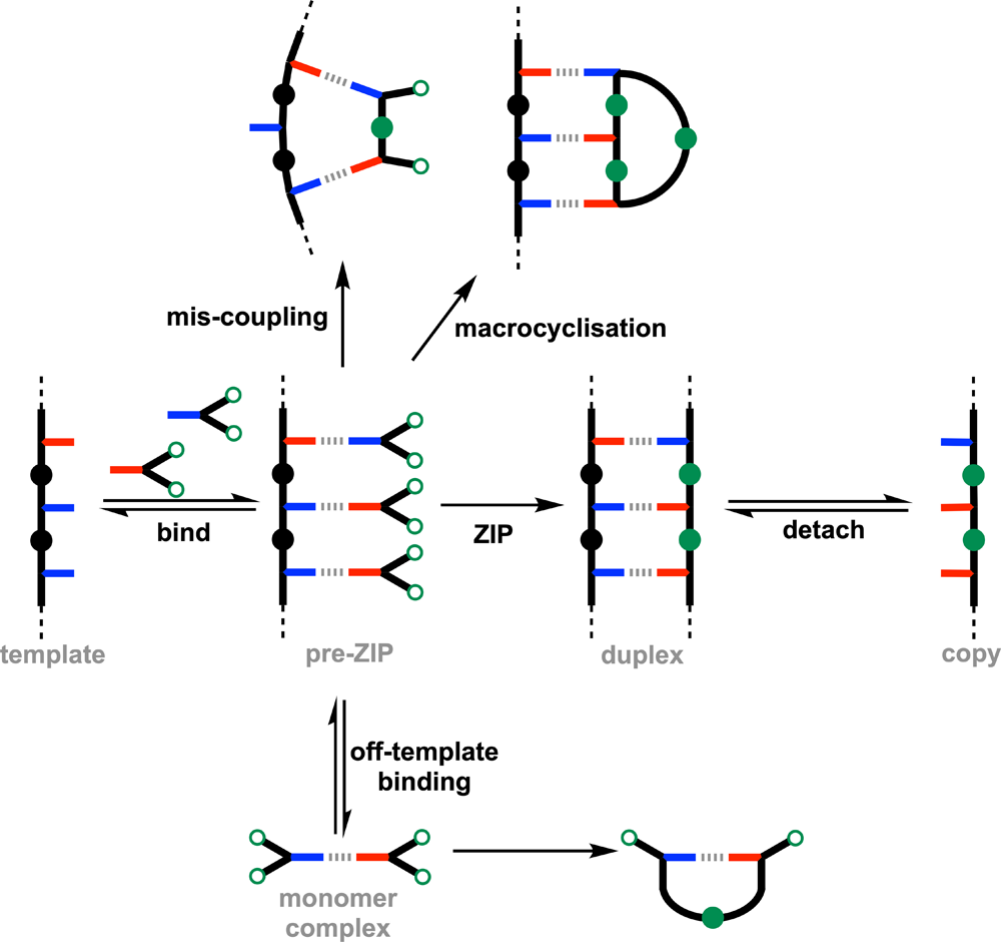
Template-directed polymerization using noncovalent base pairs. Sequence replication is based on the binding of monomers to a template followed by a ZIP reaction to assemble the backbone of the copy strand. Mis-coupling reactions on the template and off-template coupling in the monomer complex compete with the ZIP reaction.

An alternative strategy for the replication of sequence information in synthetic polymers is base-filling (Figure 2).^21,22^ In this approach, a preassembled blank backbone that is devoid of sequence information is used as the copy strand. Side-chain recognition units are attached to the blank copy strand using dynamic covalent chemistry, and a template strand is used to specify the sequence by formation of a H-bonded duplex. In this case, the formation of competing macrocyclic products is not possible. Similarly, off-template reactions are not a problem, because the recognition units cannot react with one other. Although mis-coupling may occur during base-filling, the reversible chemistry used to attach the side-chains provides error-correction pathways, which should allow equilibration to the fully complementary duplex, provided this is the most stable species. To obtain a kinetically stable copy from this process, the dynamic duplex must be trapped by an irreversible step. The trapped duplex can then be dissociated to yield the copy strand and recover the template.

**Figure 2:**
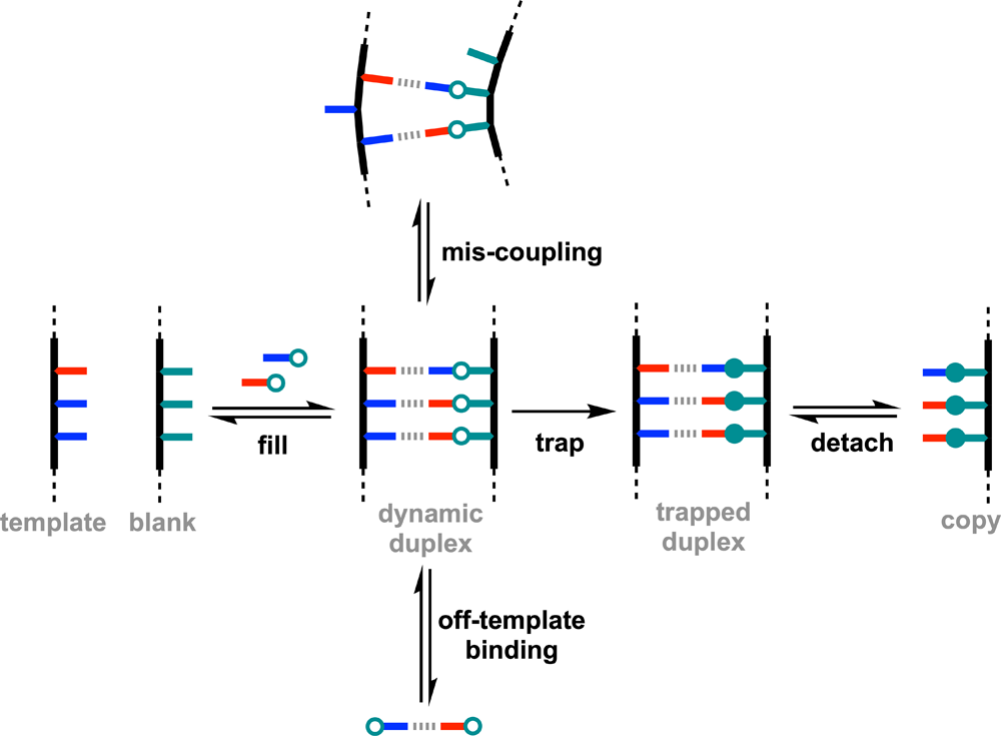
Template-directed base-filling using noncovalent base pairs (red and blue). Sequence replication is based on dynamic attachment of side-chain recognition units to the preassembled backbone of a blank copy strand. A reversible reaction is used to equilibrate complementary recognition units onto the copy strand in the dynamic duplex, which is trapped using an irreversible reaction. Mis-coupling reactions that compete with assembly of the dynamic duplex are reversible, providing pathways for error correction.

Ghadiri et al. have successfully used the base-filling approach to template the attachment of thioester nucleobase side-chains onto cysteine-functionalized PNA blank strands using DNA templates.^22,23^ Mixing the DNA template and PNA blank strands with two different nucleobase thioesters yielded copy strands that incorporated about 80% of the sequence-complementary side-chain, but it was not possible to trap the dynamic thioester products for use in subsequent replication cycles. DNA templates have also been used to fill a single blank amine site on a PNA or DNA oligomer with the complementary aldehyde nucleobase side-chain using reductive amination.^24–27^

Figure 3 shows an alternative version of the base-filling process in Figure 2, where the template and blank stranded are first linked with a covalent base-pair. This approach reduces the possibility of template-template interactions and promotes the formation of the dynamic duplex, because all of the noncovalent interactions are intramolecular. Here, we describe a base-filling strategy based on dynamic imine chemistry to template the synthesis of recognition-encoded melamine oligomers (REMO) using both of the approaches shown in Figures 2 and 3.^28^ Fidelity of over 90% was achieved in the copying process, and the dynamic imine products could be trapped by reduction and isolated as the corresponding amines.

**Figure 3:**
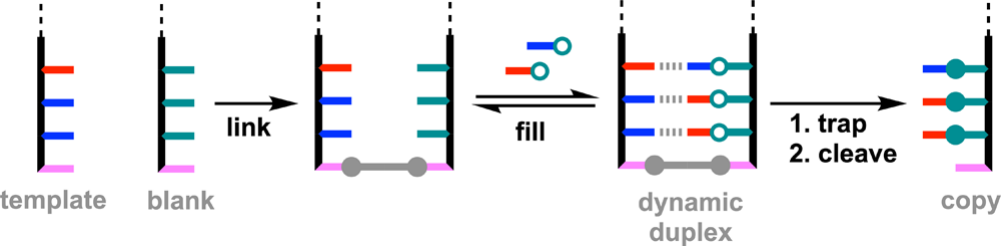
Template-directed base-filling using a covalent base pair (pink and gray) to attach the blank strand to the template. A reversible reaction is used to equilibrate complementary recognition units (red and blue) onto the copy strand in the dynamic duplex, which is trapped using an irreversible reaction. Cleavage of the covalent base pair separates the template and copy strands.

REMO have an alternating 1,3,5-triazine-piperazine backbone and side-chains equipped with recognition units.^28^ Figure 4 shows the REMO used to realize the replication schemes in Figures 2 and 3. The template strand is equipped with three 4-nitrophenol (**D**) recognition units, which form H-bonded base-pairing interactions with complementary phosphine oxide (**A**) recognition units. The blank strand is equipped with three aldehyde side chains that allow dynamic attachment of amines equipped with recognition units using imine chemistry. Both the template and blank strand are equipped with a terminal carboxylic acid, which can be used to covalently link the two strands for the approach shown in Figure 3.

**Figure 4:**
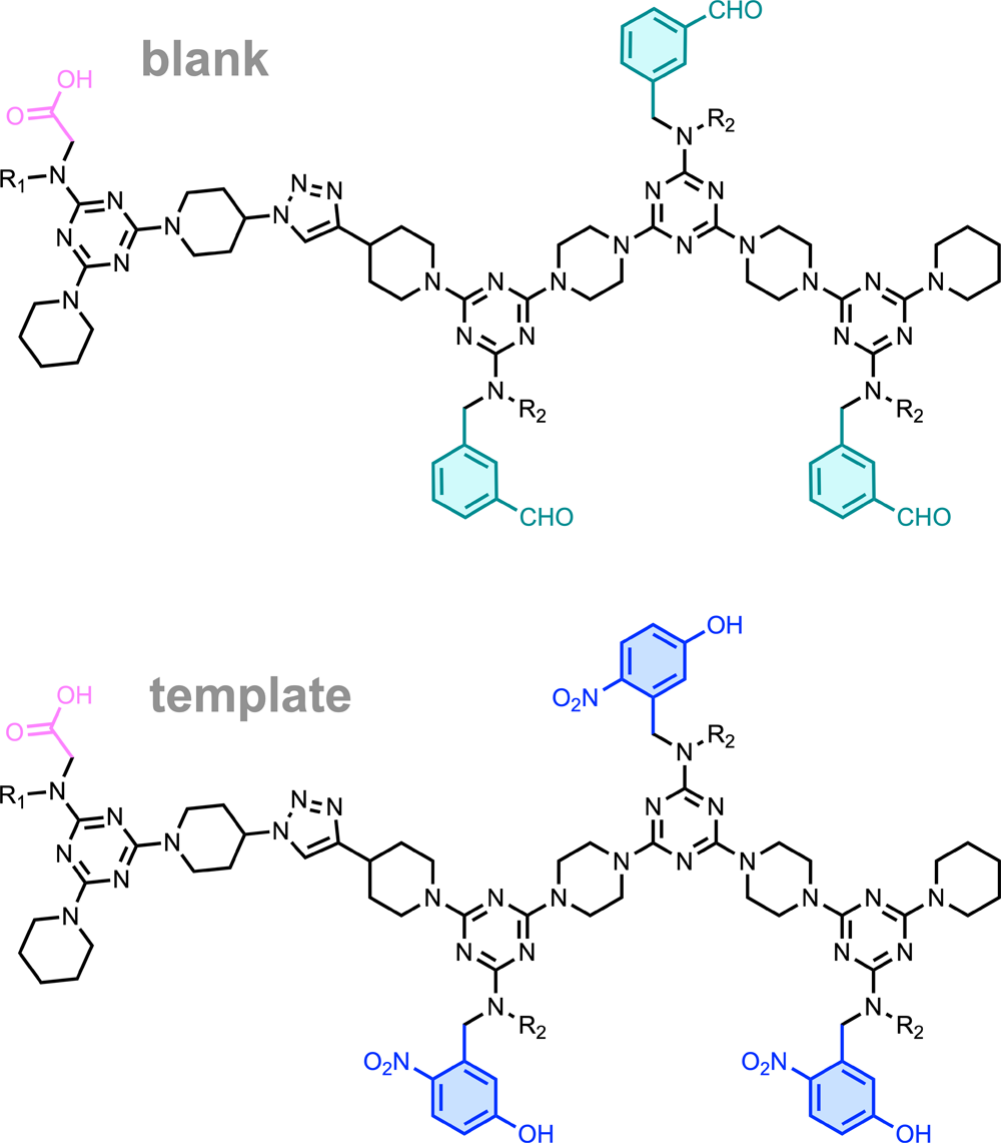
Structures of the blank and template REMO strands used to investigate the base-filling schemes in Figures 2 and 3.

## Results and Discussion

### Synthesis

An amine equipped with a phosphine oxide recognition unit (**2**) was synthesized from **1**, which is previously described.^28^ Mesylation of the alcohol, nucleophilic substitution with 4-methoxybenzylamine, and hydrogenation to remove the 4-methoxybenzyl group gave **2** in good yield (Scheme 1).

**Scheme 1:**
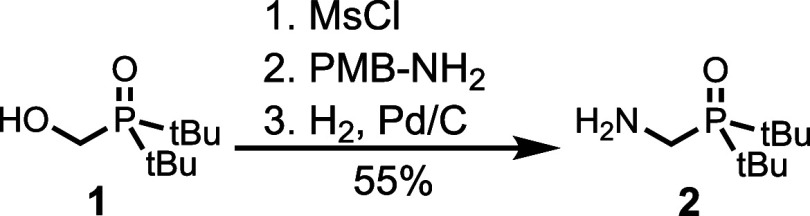
Synthesis of an Amine Equipped with a Phosphine Oxide Recognition Unit

Synthesis of the REMO template and blank strands used the dichlorotriazine building blocks shown in Scheme 2. The protected carboxylic acid dichlorotriazine **3** was synthesized by reductive amination of *iso*-butanal with *tert*-butyl aminoacetate hydrochloride, followed by reaction with cyanuric chloride. To make the aldehyde dichlorotriazine **5**, 3-(diethoxymethyl)­benzaldehyde was reacted with 2-ethylhexylamine then sodium borohydride to yield the secondary amine **4**. Reaction of **4** with cyanuric chloride yielded the corresponding dichlorotriazine, which was subjected to hydrochloric acid to deprotect the aldehyde and give **5**. The 4-nitrophenol dichlorotriazine **6** was synthesized as previously described.^29^

**Scheme 2:**
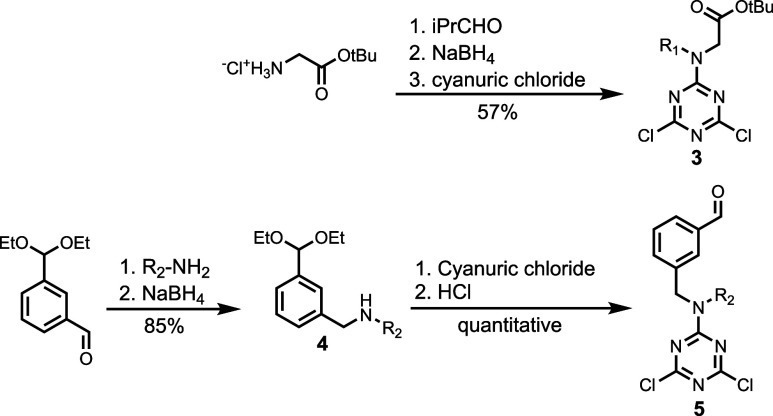
Synthesis of Dichlorotriazine Building Blocks^a^

a R_1_ = *iso*-butyl. R_2_ = 2-ethylhexyl.

REMO 3-mers equipped with aldehyde or 4-nitrophenol side-chains and a terminal alkyne were synthesized via sequential S_N_Ar coupling reactions (Scheme 3). Each of the dichlorotriazines was substituted with piperidine then piperazine to yield **7a** and **7b**. *Tert*-butyl-4-ethynylpiperidine-1-carboxylate was treated with TFA to remove the Boc protecting group then reacted with either dichlorotriazine **5** or **6**. The resulting monochlorotriazines were both substituted with piperazine to yield **8a** and **8b**, which were then reacted with a further equivalent of either **5** or **6** to yield **9a** and **9b**. Finally, substitution of **9a** and **9b** with either **7a** or **7b** yielded the two REMO 3-mers **10a** and **10b**. Deprotection of **10b** with TFA gave **11**, which was used as the template strand in the approach outlined in Figure 2.

**Scheme 3:**
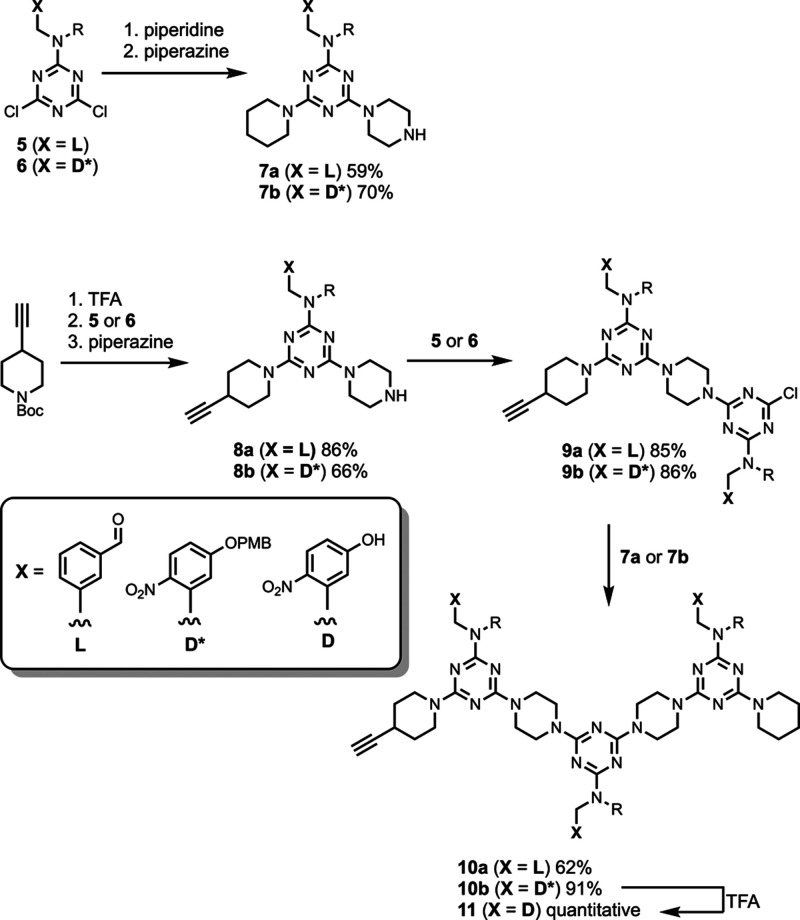
Synthesis of REMO 3-Mers^a^

a R = 2-ethylhexyl.

To attach carboxylic acid recognition units to the end of each REMO 3-mer, the 1-mer **15**, which is equipped with a carboxylic acid recognition unit and an azide, was synthesized (Scheme 4). The 4-azidopiperidine derivative **12** was prepared as previously described,^30^ deprotected with TFA and coupled with dichlorotriazine **3** to give **13**. The monochlorotriazine was then substituted with piperidine to give **14** and treated with TFA to deprotect the *tert*-butyl ester and yield the carboxylic acid **15**. REMO 1-mer **15** was then attached to each of **10a** and **10b** by a copper-catalyzed azide–alkyne cycloaddition (CuAAC) reaction to give the blank strand **16a** and template **16b**.

**Scheme 4:**
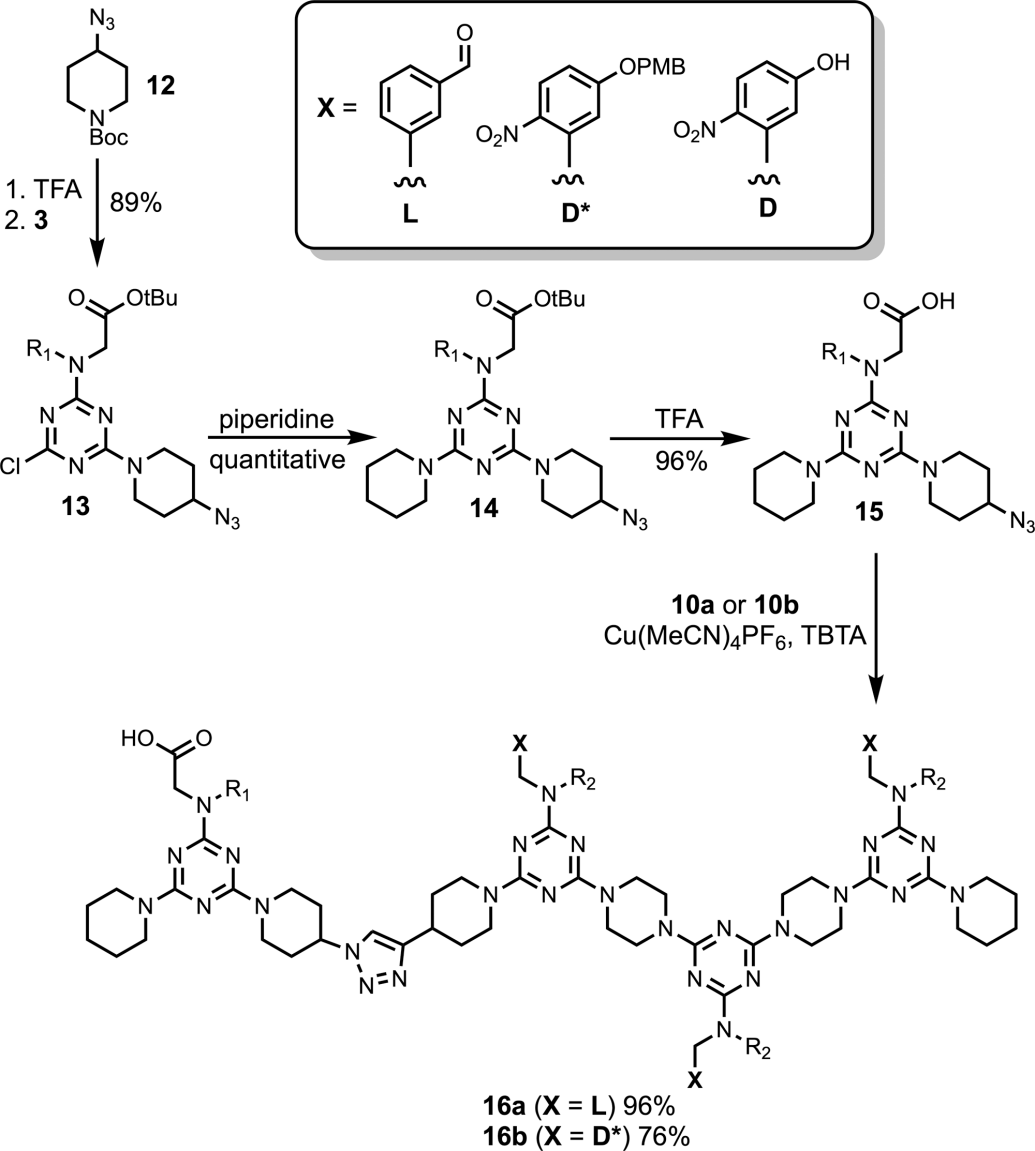
Synthesis of **16a** and **16b** ^a^

a R_1_ = *iso*-butyl, R_2_ = 2-ethylhexyl.

To study the templating scheme in Figure 3, the carboxylic acid recognition units on the template and blank strands were functionalized with an azide and an alkyne respectively, and a CuAAC reaction was used to link the two strands (Schemes 5 and 6). Azide **17**, which was prepared as previously described,^31^ was attached to **16a** by esterification with EDC and DMAP giving **18** (Scheme 5). Propargyl alcohol was attached to the carboxylic acid of **16b** by esterification to give **19** (Scheme 6). **18** and **19** were then coupled using a CuAAC reaction to give **20**, and deprotection of the 4-nitrophenol recognition units with TFA yielded the covalently linked strands **21**.

**Scheme 5:**
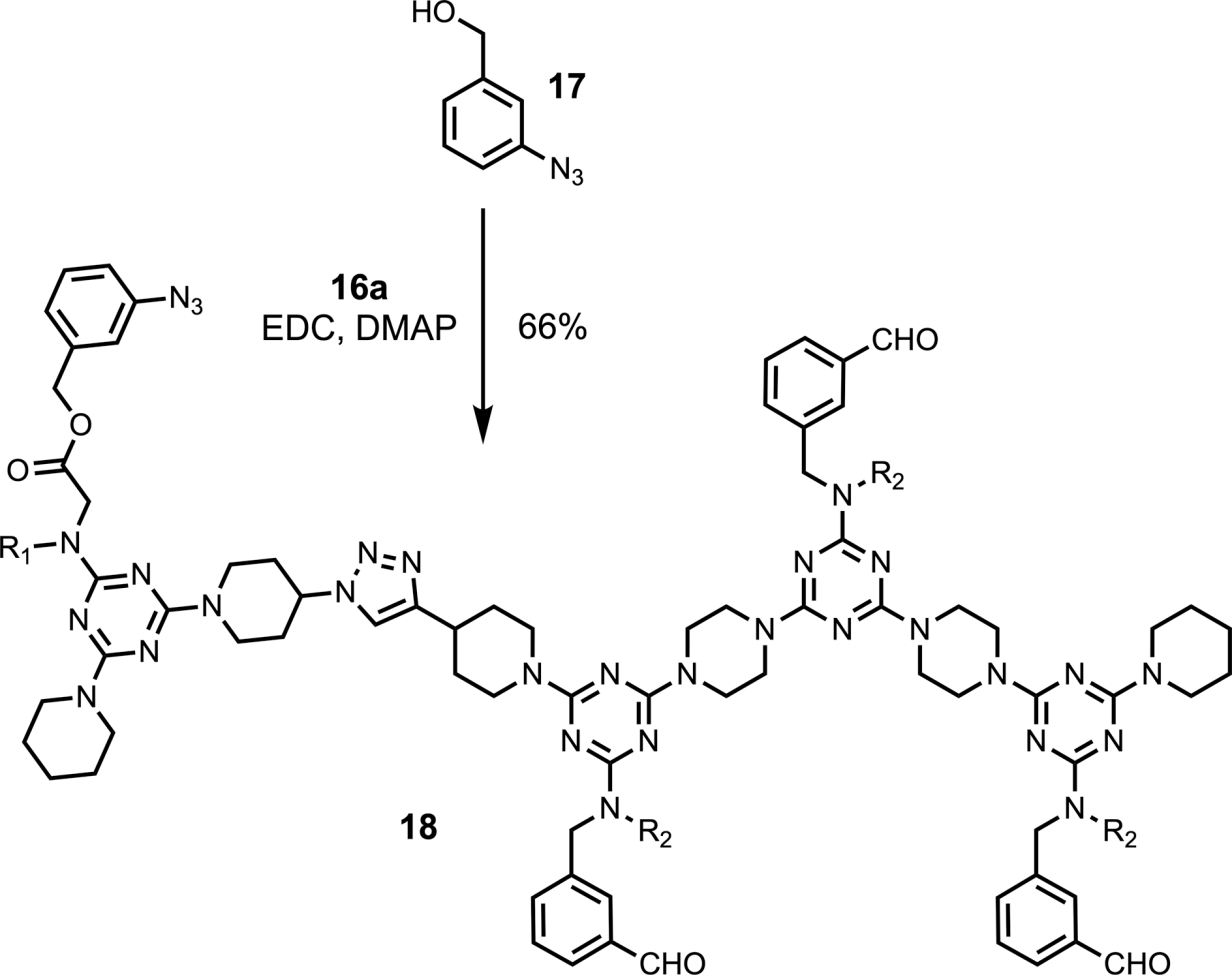
Synthesis of **18** ^a^

a R_1_ = *iso* **-**butyl, R_2_= 2-ethylhexyl.

**Scheme 6:**
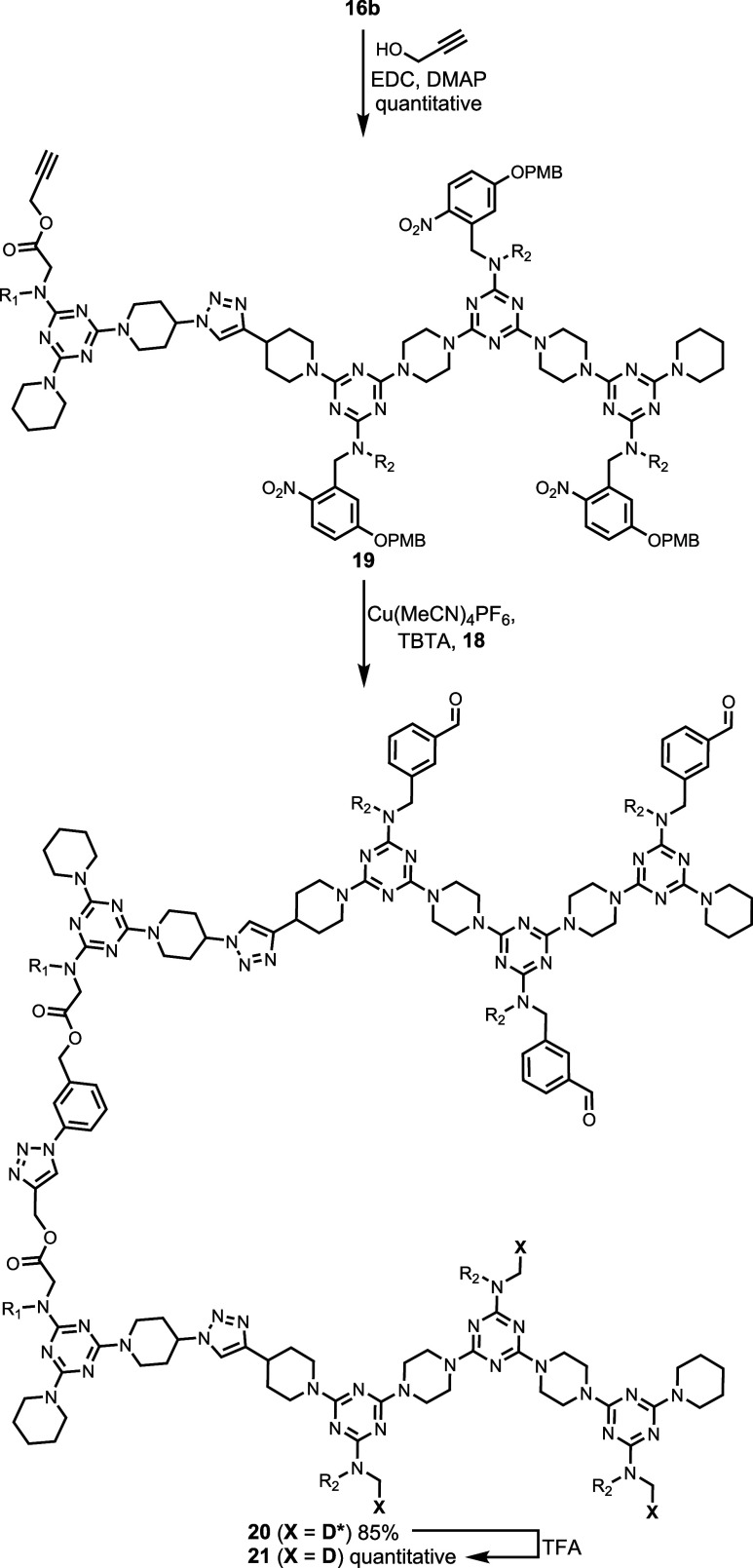
Synthesis of **21** ^a^

a R_1_ = *iso*-butyl, R_2_ = 2-ethylhexyl.

### Base-Filling an Unlinked Blank Strand

NMR spectroscopy was used to monitor dynamic attachment of the side chains in the base-filling scheme shown in Figure 2. Template strand **11**, which has the sequence **DDD**, was dissolved in deuterodichloromethane and mixed with the blank strand **18** (1 equiv), the phosphine oxide amine **2** (6 equiv) and benzylamine (6 equiv). Benzylamine was added as a competing amine that cannot bind to the template but has very similar reactivity to **2** (Figure S47). The ^1^H NMR spectrum was monitored over a period of 24 h, but the system had equilibrated after the first hour, and no further changes were observed. Comparison of the initial mixture (Figure 5a) and the equilibrated mixture (Figure 5b) showed complete disappearance of the signal at 10 ppm due to the aldehydes on the blank strand and the appearance of two new signals between 8.0 and 8.5 ppm. Control experiments mixing the blank strand with only one of the two amines showed that these two signals correspond to the two different types of imine, which give well-resolved ^1^H NMR signals (highlighted in gray for the benzylamine imine and red for the phosphine oxide imine in Figure 5b). The relative intensity of these two peaks therefore provides a direct readout of the magnitude of the template effect: if the base-filling worked perfectly, there would be only one imine signal corresponding to exclusive incorporation of the phosphine oxide. Figure 5b shows that at a concentration of 1 mM template, benzylamine was incorporated into the blank strand in similar proportions to the phosphine oxide. Integration of the two imine signals was used to quantify the template effect on the product distribution, and at 1 mM template, the selectivity for the phosphine oxide was 67:33. However, when the system was diluted, the proportion of phosphine oxide imine increased significantly, and at 0.1 mM template, the selectivity for the phosphine oxide increased to 79:21.

**Figure 5:**
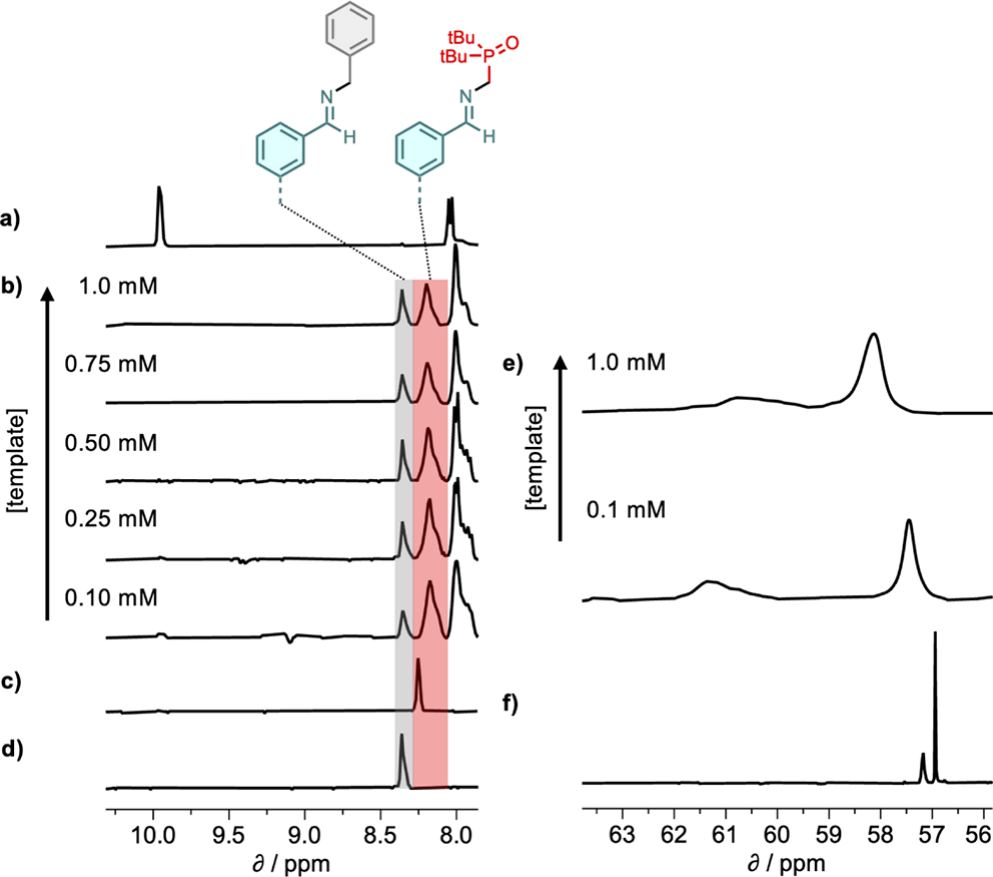
Left: partial ^1^H NMR spectra (400 MHz, CD_2_Cl_2_, 298 K) of (a) a mixture of **11** (1 mM), **18** (1 mM), **2** (6 mM), and benzylamine (6 mM), (b) equilibrated mixtures of **11** (1 equiv), **18** (1 equiv), **2** (6 equiv) and benzylamine (6 equiv) at different template concentrations, (c) an equilibrated mixture of **18** (1 mM) and **2** (6 mM) (imine signal highlighted in red), (d) an equilibrated mixture of **18** (1 mM), and benzylamine (6 mM) (imine signal highlighted in gray). Right: ^31^P NMR spectra (162 MHz, CD_2_Cl_2_, 298 K) of (e) equilibrated mixtures of **11** (1 equiv), **18** (1 equiv)¸ **2** (6 equiv), and benzylamine (6 equiv) at different template concentrations, and (f) an equilibrated mixture of **18** (1 mM) and **2** (6 mM).

Figure 5e shows the corresponding ^31^P NMR spectra. When the blank strand **18** was mixed with an excess of **2** in the absence of template, two ^31^P signals were observed, a sharp signal at 57 ppm due to the amine and broader signal with a small downfield shift due to the imine. In the presence of the template strand, both ^31^P NMR signals became very broad and shifted downfield, which suggests that both the phosphine oxide imines on the copy strand and the excess of **2** in solution are involved in H-bonding interactions with the template. For the signal due to the phosphine oxide imine (60–61 ppm at 1 mM template), the size of the downfield shift and the broadening were both much larger than for the corresponding signal due to the phosphine oxide amine, which suggests that the 4-nitrophenol recognition groups on the template strand preferentially form H-bonds with phosphine oxides on the copy strand than with the excess **2** in solution. However, it is clear than the excess **2** does compete for the recognition sites on the template. On dilution of the solution to 0.1 mM of template, the ^31^P signal due to the excess **2** moved upfield and sharpened, but the signal due to the imine shifted further downfield. This result shows that the preference for H-bonding interactions with the phosphine oxide imines on the copy strand increases as the concentration decreases, because there is reduced competition for the cooperative H-bonds in the duplex from the noncooperative intermolecular interactions with the excess of **2** in solution.

The NMR data reveal that the dynamic covalent chemistry used for base-filling is controlled by the interplay of different intermolecular and intramolecular interactions. Figure 6 illustrates all possible species that are present in equilibrium: template and copy strands that are not H-bonded to one another (purple and red boxes), the fully assembled duplex formed by the template and the sequence-complementary copy (blue box), frayed duplexes with missing H-bonds (gray box), and duplexes that are partially denatured by H-bonding to the excess of **2** (green box). The speciation in the equilibrated mixture is governed by just three parameters: the association constant for the formation of an intermolecular 4-nitrophenol·phosphine oxide H-bond, *K*_h_; the equilibrium constant that quantifies the difference between the stabilities of the two types of imine, *K*_i_; and the effective molarity (EM) for the formation of intramolecular H-bonding interactions in the duplex, assuming that stepwise effective molarities for formation of intramolecular H-bonds after formation of the first intermolecular H-bond are the same.

**Figure 6:**
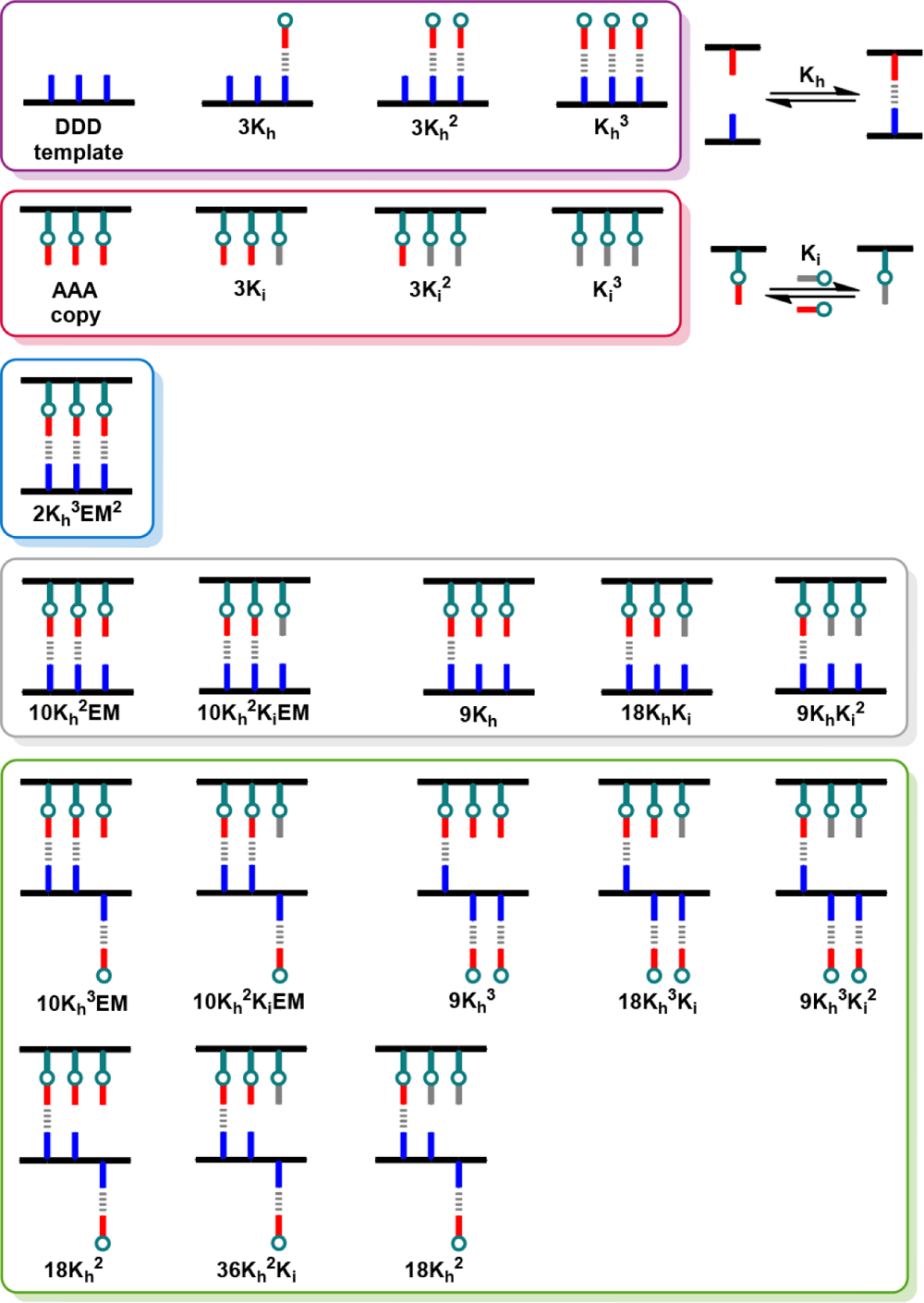
Cartoon representation of the species present in the imine library formed from a mixture of **11**, **18**, **2**, and benzylamine. The global equilibrium constant relative to the free **DDD** template, the free **AAA** copy, and the two free amines is shown for each species. *K*_h_ is the association constant for formation of a 4-nitrophenol·phosphine oxide H-bond, *K*_i_ is the equilibrium constant for imine exchange between **2** and benzylamine, EM is the effective molarity for any intramolecular H-bond formed within a duplex, and the statistical factors reflect the degeneracy of each species (only one of each sequence isomer is illustrated). Species are grouped in boxes as single stranded templates (purple), single stranded copies (red), copy strands that are present as the fully assembled duplex (blue), duplexes with frayed H-bonds (gray), and duplexes that are partially denatured due to interaction with **2** (green).

Figure 6 shows the equilibrium constants for formation of each species starting from the free **DDD** template, the free **AAA** copy and the two free amines. The value of *K*_i_ was independently determined in a separate experiment (*K*_i_ = 0.55, see Supporting Information), *K*_h_ was measured previously (*K*_h_ = 1,500 M^–1^).^29^ The EM is the only unknown variable, and the experimental data in Figure 5a were used to find the value of EM that best describes the results. The total yield of phosphine oxide imine in an equilibrated solution (*P*_PO_) can be calculated from the integrals of the signals due to the two types of imine (eq 1).

1 $$P_{\mathrm{PO}}=\frac{I_{\mathrm{PO}}}{I_{\mathrm{PO}}+I_{B}}$$

where *I*_PO_ and *I*_B_ are the integrals of the signals due to the phosphine oxide and benzyl imine signals, respectively.

Given the total concentration of each of the components added to the mixture and a value for EM, the model in Figure 6 can be used in the Musketeer software to calculate the population of each species, and the total yield of phosphine oxide imine (*P*_PO_) can be used to optimize the value of EM to obtain the best fit to the experimental data (see Supporting Information for details).^32^ Figure 7a compares the total yield of phosphine oxide imine calculated using the best fit EM of 10 mM (line) with the experimental results in Figure 5a (data points).

**Figure 7:**
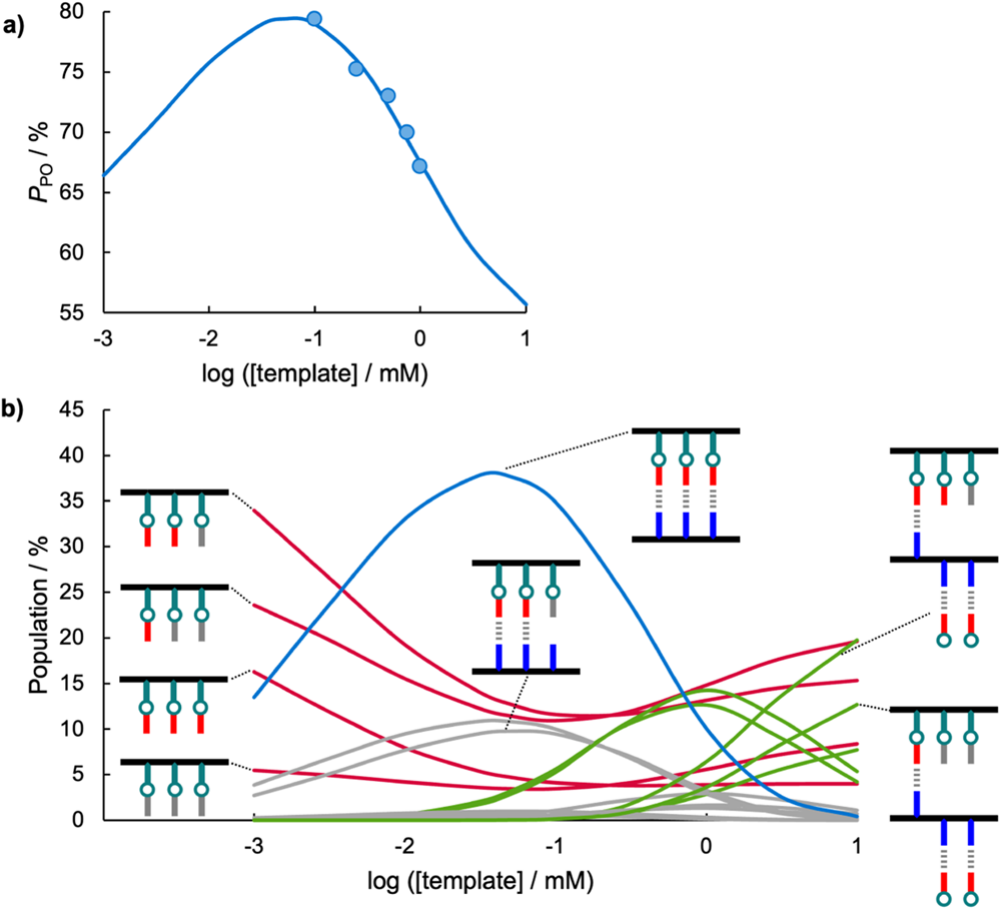
(a) Total yield of phosphine oxide imines (*P*_PO_) in equilibrated mixtures of template **11** (1 equiv), **18** (1 equiv), **2** (6 equiv), and benzylamine (6 equiv) in CD_2_Cl_2_ (data points) compared with the prediction based on the model shown in Figure 6 using *K*_h_ = 1,500 M^–1^, *K*_i_ = 0.55, and EM = 10 mM (line). (b) Calculated populations of species shown in Figure 6 plotted as a percentage of the total concentration of copy strands. Lines are colored according to the groupings shown in Figure 6, and cartoons of representative species are shown.

Figure 7b shows the calculated speciation as a function of template concentration. At a template concentration of 10 mM, the copy strands are predominantly present either as partially denatured duplexes (green) or as free strands that are not bound to the template (red), because the 4-nitrophenol recognition units on the template interact preferentially with the excess phosphine oxide amine in solution. As the solution is diluted below the EM, intramolecular H-bonding interactions become more favorable than intermolecular interactions, and the proportion of copy strands present as the fully formed duplex increases. These results are consistent with the observed changes in the ^31^P NMR spectra described above. The proportion of duplex present peaks at around 40% between 0.1 and 0.01 mM of template and is then predicted to decrease as the solution is diluted further. The reason for this behavior is that the association constant for formation of the duplex is 7 × 10^5^ M^–1^ (=2*K*_h_^3^EM^2^), so the duplex starts to dissociate at lower concentrations. As the duplex falls apart, the copy strands are left free in solution, and the imine populations revert to a statistical distribution at low concentrations. Although the yield of phosphine oxide imine obtained at 0.1 mM of template was 79%, Figure 7b suggests that if the duplex could be prevented from dissociating at low concentrations, then higher yields might be obtained. An obvious method for inhibiting dissociation of the duplex is to covalently attach the blank strand to the template strand.

### Base-Filling a Covalently Linked Blank Strand

The speciation model shown in Figure 6 was adapted for a system with a covalent linker between the template and copy strands by eliminating all of the species in the purple and red boxes and making all of the duplex H-bonding interactions in the blue, gray and green boxes intramolecular (see Supporting Information for details). Figure 8 shows the speciation predicted for the covalently linked system as a function of template concentration using the same values of *K*_i_, *K*_h_ and EM used in Figure 7. Again, at concentrations above the EM (10 mM), the fully formed duplex is not populated (blue), and partially denatured duplexes dominate (green). However, upon dilution to 0.1 mM of template, a very significant increase in the population of duplex is predicted (70%), and this population persists at lower concentrations. In contrast to the unlinked system, where the population of fully formed duplex never exceeded 40%, this model predicts that the covalently linked system should result in twice as much duplex at low concentrations.

**Figure 8:**
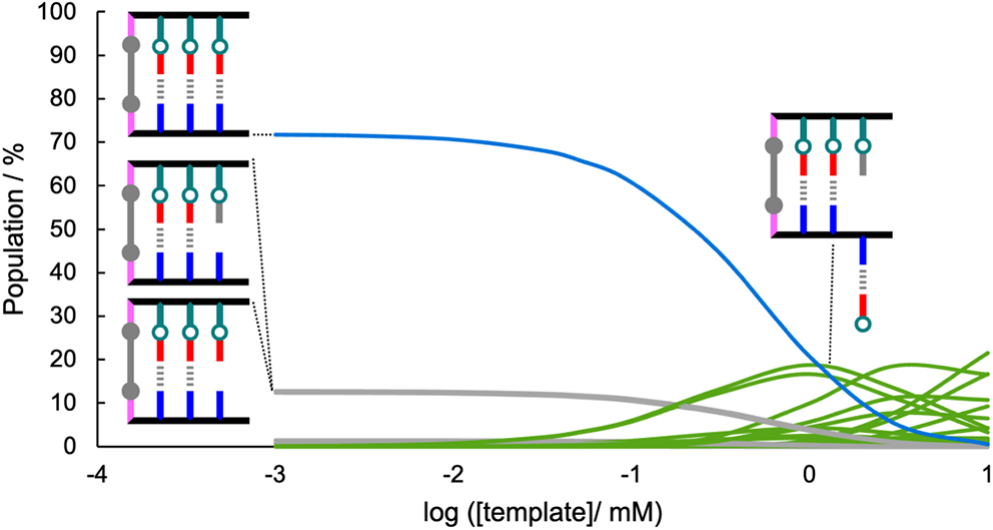
Calculated populations of species in equilibrated mixtures of template **21** (1 equiv), **2** (6 equiv), and benzylamine (6 equiv) in dichloromethane using *K*_h_ = 1,500 M^–1^, *K*_i_ = 0.55, and EM = 10 mM. The fully assembled duplex is shown in blue; duplexes with frayed H-bonds are shown in gray; and duplexes that are partially denatured due to interaction with **2** are shown in green (see Supporting Information for details). Cartoon representations of the major species present are shown.

To experimentally test these predictions, template **21** (10 mM) was mixed with **2** and benzylamine (each 6 equiv) in deuterodichloromethane, and the solution was equilibrated. The solution was then diluted to template concentrations of 1 mM and 0.1 mM, and these solutions were allowed to re-equilibrate (see Supporting Information for details). ^1^H NMR spectra of the mixtures at each concentration were recorded, and the imine region was analyzed as before to determine the total yield of each type of imine in the mixtures (again no signal due to unreacted aldehyde was observed at 10 ppm). At 10 mM of template, benzylamine was incorporated into the blank strand in similar proportions to the phosphine oxide, and the ^1^H NMR spectrum shown in Figure 9a looks very similar to the unlinked system shown in Figure 5b. However, at 0.1 mM of template, the yield of phosphine oxide imine was 87%. Integration of the imine signals highlighted in red and gray in Figure 9a was used to quantify the relative yields of the two types of imine as a function of template concentration (Figure 10).

**Figure 9:**
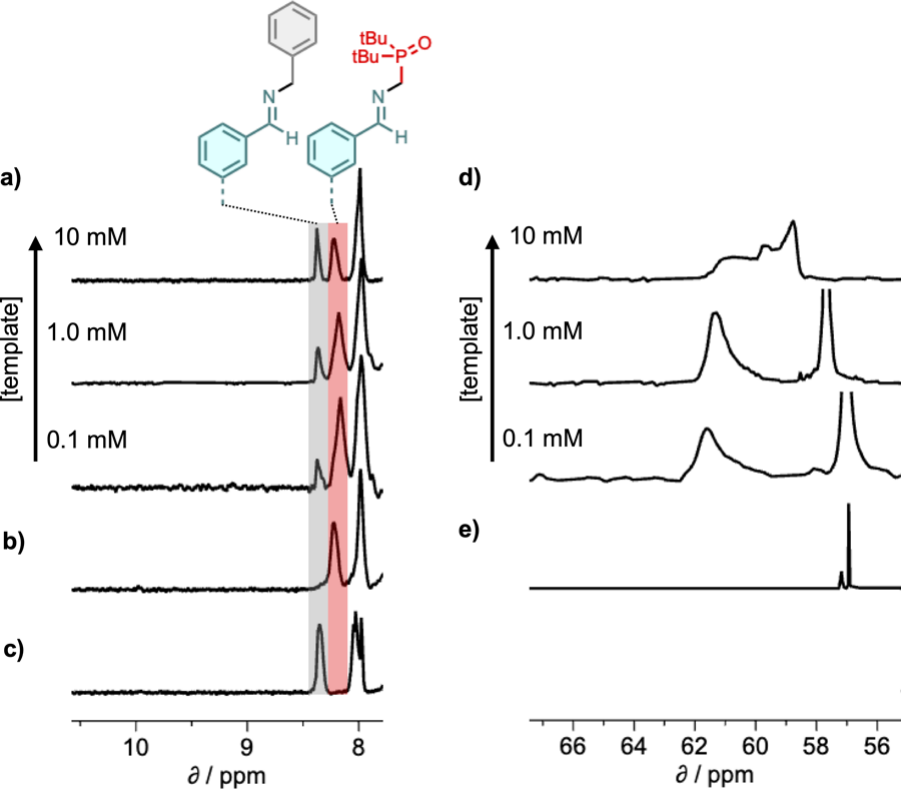
Left: partial ^1^H NMR spectra (400 MHz, CD_2_Cl_2_, 298 K) of (a) equilibrated 1:6:6 mixtures of **21**, **2**, and benzylamine at different template concentrations, (b) an equilibrated mixture of **21** (1 mM) and **2** (6 mM) (imine signal highlighted in red), (c) an equilibrated mixture of **21** (1 mM) and benzylamine (6 mM) (imine signal highlighted in gray). Right: ^31^P NMR spectra (162 MHz, CD_2_Cl_2_, 298 K) of (d) equilibrated 1:6:6 mixtures of **21**, **2**, and benzylamine at different template concentrations, and (e) an equilibrated mixture of **18** (1 mM) and **2** (6 mM).

**Figure 10:**
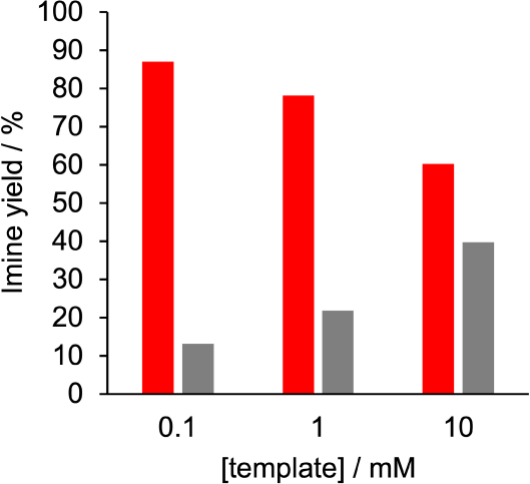
Total yields of phosphine oxide imines (red) and benzyl imines (gray) in equilibrated mixtures of template **21** (1 equiv), **2** (6 equiv), and benzylamine (6 equiv) in CD_2_Cl_2_.

The ^31^P NMR spectrum of each equilibrated mixture was also recorded (Figure 9d), and the results are consistent with the speciation shown in Figure 8. At 10 mM of template, the signals were broad and overlapping, indicating that both phosphine oxides attached to the copy strand as imines and phosphine oxides in solution as amines H-bond to the 4-nitrophenol recognition units on the template with little selectivity. However, on dilution, two distinct signals were observed. The broad signal at 61–62 ppm is indicative of a H-bonded phosphine oxide, and the sharp signal at 57 ppm is the same as the signal observed for amine **2** in the absence of template. This result is consistent with formation of the fully H-bonded duplex with no competition for H-bonding interactions with the excess **2** in solution. It is possible that the duplex could open up and form a dimeric duplex of double the molecular weight at high concentrations, but ^1^H NMR DOSY experiments indicate that there was no dimerization at concentrations lower than 1 mM (see Supporting Information).

The speciation profile in Figure 8 shows that the population of the fully assembled duplex reaches a plateau at low concentrations, and the only other species present are frayed duplexes. When a H-bond in the duplex is broken, the imines can re-equilibrate, with the resulting benzyl imines limiting the fidelity of the base-filling process. Figure 11 illustrates the general result obtained at low concentrations for base-filling using a covalently linked blank strand on a template with N recognition units. Since duplex assembly only takes place when *K*_h_ EM ≫ 1, the amount of fully assembled duplex (**I**) is largely limited by breaking one H-bond leading to **II**, and the subsequent imine exchange that leads to **III**. The limiting fidelity of the base-filling process at low concentrations can therefore be estimated using the values of *K*_h_, *K*_i_ and EM in eqs 4.

2 $$\left[\mathrm{II}\right]=\frac{N}{K_{h}EM}\left[I\right]$$

3 $$\left[\mathrm{III}\right]=K_{i}\left[\mathrm{II}\right]=\frac{NK_{i}}{K_{h}EM}\left[I\right]$$

4 $$\mathrm{Fidelity}=\frac{\left[I\right]+\left[\mathrm{II}\right]}{\left[I\right]+\left[\mathrm{II}\right]+\left[\mathrm{III}\right]}=\frac{K_{h}EM+N}{K_{h}EM+N\left(1+K_{i}\right)}$$

**Figure 11:**
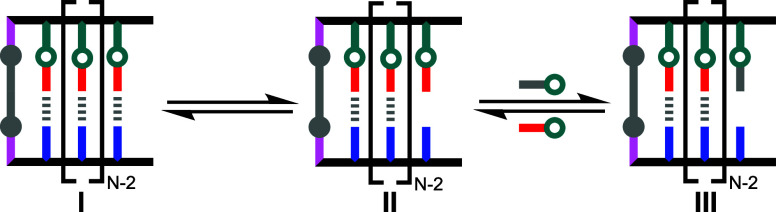
At low concentrations only species **I**, **II**, and **III** are significantly populated for a duplex with N base pairs. There are *N* sequence isomers of species **II** and **III**, but only one is shown.

Equation 4 shows that as the length of the template strand (*N*) increases, the fidelity of base-filling will decrease. Fidelity could be increased by increasing either the effective molarity for duplex formation (EM) or the association constant for formation of a H-bond (*K*_h_). The value of EM is an intrinsic property of the system and is difficult to predict. However, it is possible to increase the strength of the H-bonding interactions by using a less polar solvent. Therefore, the effect of changing the solvent from dichloromethane to toluene was investigated. It is possible to predict the change in the value of *K*_*h*_ using the H-bond parameters for 4-nitrophenol (α = 4.7) and phosphine oxide (β = 10.2) in eq 5 ^33^

5 $$\Delta G^{{}^{\circ}}/\mathrm{kJ}{\mathrm{mol}}^{-1}=-RT\ln K_{h}=-\left(\alpha -\alpha_{S}\right)\left(\beta -\beta_{S}\right)+6$$

where α and β are the H-bond parameters of the two solutes, and α_S_ and β_S_ are the H-bond parameters for the solvent (1.1 and 2.1 for toluene).

Equation 5 predicts an increase of *K*_h_ from 1,500 M^–1^ in dichloromethane to 11,000 M^–1^ in toluene solution, and Figure 12 shows the corresponding speciation profile calculated for the covalently linked base-filling process using different concentrations of template **21** in toluene solution. The yield of the fully assembled duplex shown in blue is limited by the amount of frayed duplex (gray line), which is inversely proportional to the product *K*_h_ EM, and since *K*_h_ is larger in toluene, the predicted yield of the sequence-complementary copy strand is increased significantly compared with dichloromethane (cf Figure 8). The upper limit on the operating concentration is still fixed by the value of EM, which determines the point at which the excess of **2** in solution starts to denature the duplex (green line).

**Figure 12:**
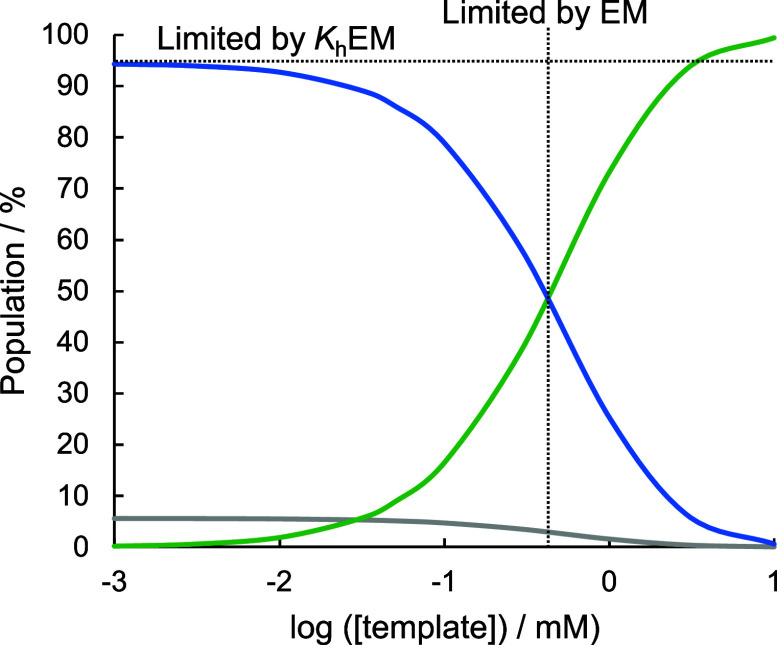
Calculated populations of species in equilibrated mixtures of covalently linked template and copy (1 equiv), phosphine oxide amine (6 equiv), and benzylamine (6 equiv) in toluene using *K*_h_ = 11,000 M^–1^, *K*_i_ = 0.55, and EM = 10 mM. The fully assembled duplex is shown in blue; the population of all duplexes with frayed H-bonds is shown in gray, and the population of all duplexes that are partially denatured due to interaction with phosphine oxide amine is shown in green (see Supporting Information for details). The limits on the population of the fully assembled duplex due to denaturation (EM) and fraying (*K*_h_ EM) are indicated.

The equilibrated mixture of **21** (1 equiv), **2** (6 equiv) and benzylamine (6 equiv) from the 0.1 mM template experiment in dichloromethane was used as a starting point for experiments in toluene. The solvent was removed, and the sample was dissolved in toluene and re-equilibrated for 1 day (see Supporting Information). The imine region of the ^1^H NMR spectrum of the resulting mixture showed broad overlapping peaks, which could not be resolved. The product distribution was therefore determined by UPLC after trapping and cleaving the copy strand. The imines were reduced with trichlorosilane and dimethylformamide to trap the products, and the ester linkers were hydrolyzed with lithium hydroxide to cleave the template from the copy strand.

Figure 13a shows the UPLC trace of the resulting product mixture. The **DDD** template was recovered intact, and a single peak was observed for the copy strand. The mass spectrum of the peak corresponding to the copy strand was used to establish the composition of the different oligomers present in the mixture. The major product was the copy with three phosphine oxides **A3**, which by definition has the sequence **AAA**, i.e., the fully sequence-complementary copy of the template (Figure 13b). Small quantities of mismatch copies containing one benzylamine **A2O** or two benzylamines **AO2** can be seen in the enlargement of the 4^+^ region of the mass spectrum in Figure 13c. To estimate the yield of each copy strand, the extracted ion intensities of the 4^+^ peaks were integrated (Figure 13e). Assuming all copy strands have similar ionizability, the yield of the sequence-complementary **AAA** copy was determined to be 85%. The total phosphine oxide imine yield (*P*_PO_) can also be estimated from these data, and the result is 92%, which represents a significant improvement compared with the experiments in dichloromethane. The trapping and hydrolysis steps used to analyze the templating process in toluene formally complete the base-filling cycle, which is illustrated in full in Figure 14.

**Figure 13:**
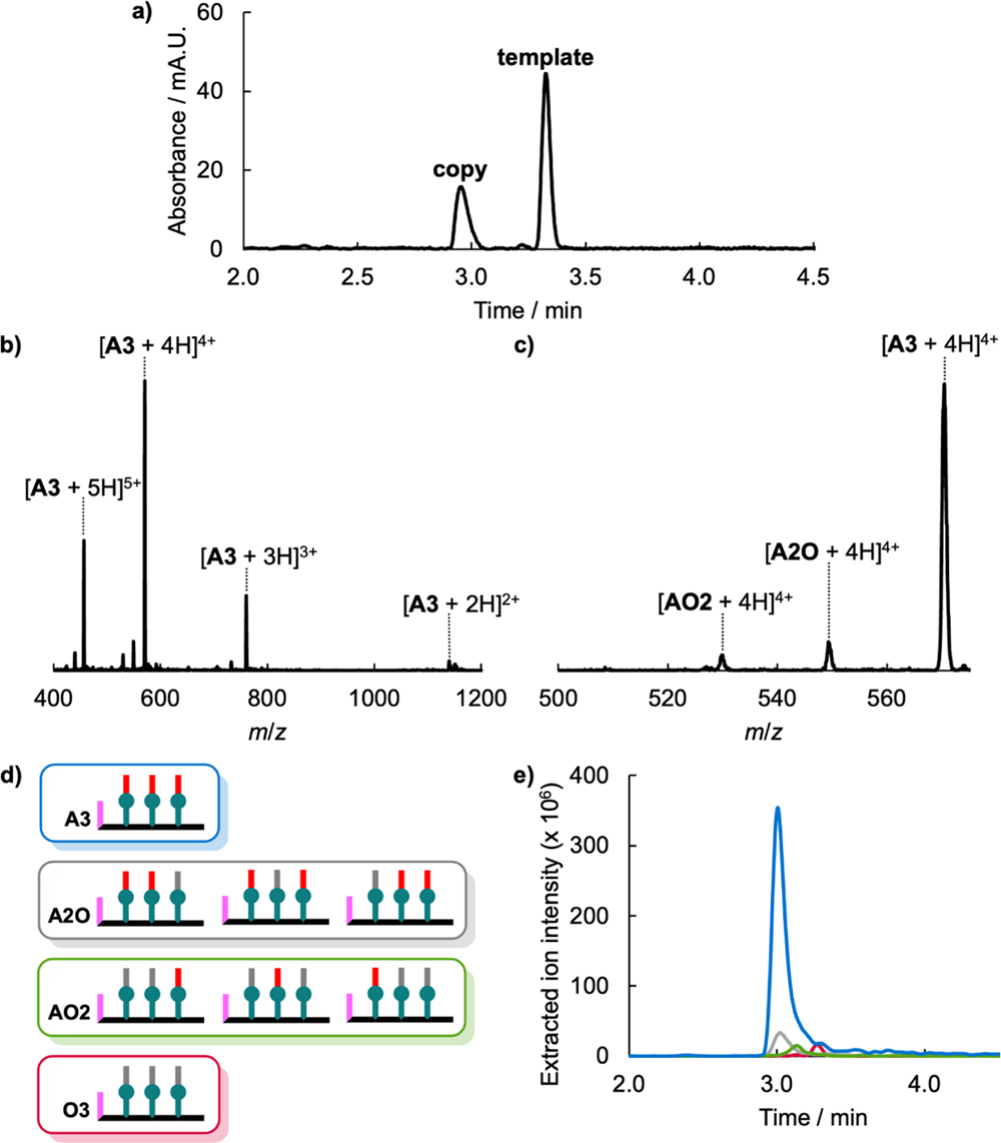
(a) UPLC trace of the crude product mixture obtained after equilibrating a mixture of template **21** (0.1 mM), **2** (0.6 mM), and benzylamine (0.6 mM) in toluene, then reducing with trichlorosilane and dimethylformamide, and hydrolyzing with lithium hydroxide. UPLC conditions: C4 column at 40 °C (254 nm) using water + 0.1% formic acid (A) and THF + 0.1% formic acid (B); gradient of 0–4 min 30–100% B + 2 min 100% B. (b) Mass spectrum of the peak labeled copy in the UPLC trace, and (c) expansion of the 4^+^ region. Strands are labeled according to the composition of recognition units (**A** indicates phosphine oxide and **O** indicates benzyl), and the structures of the copy strands, grouped according to the recognition unit composition, are shown in (d). (e) Extracted ion chromatograms for the [M + 4H]^4+^ ions from the peak labeled copy in the UPLC trace: **A3** (blue), **A2O** (gray), **AO2** (green), and **O3** (red).

**Figure 14:**
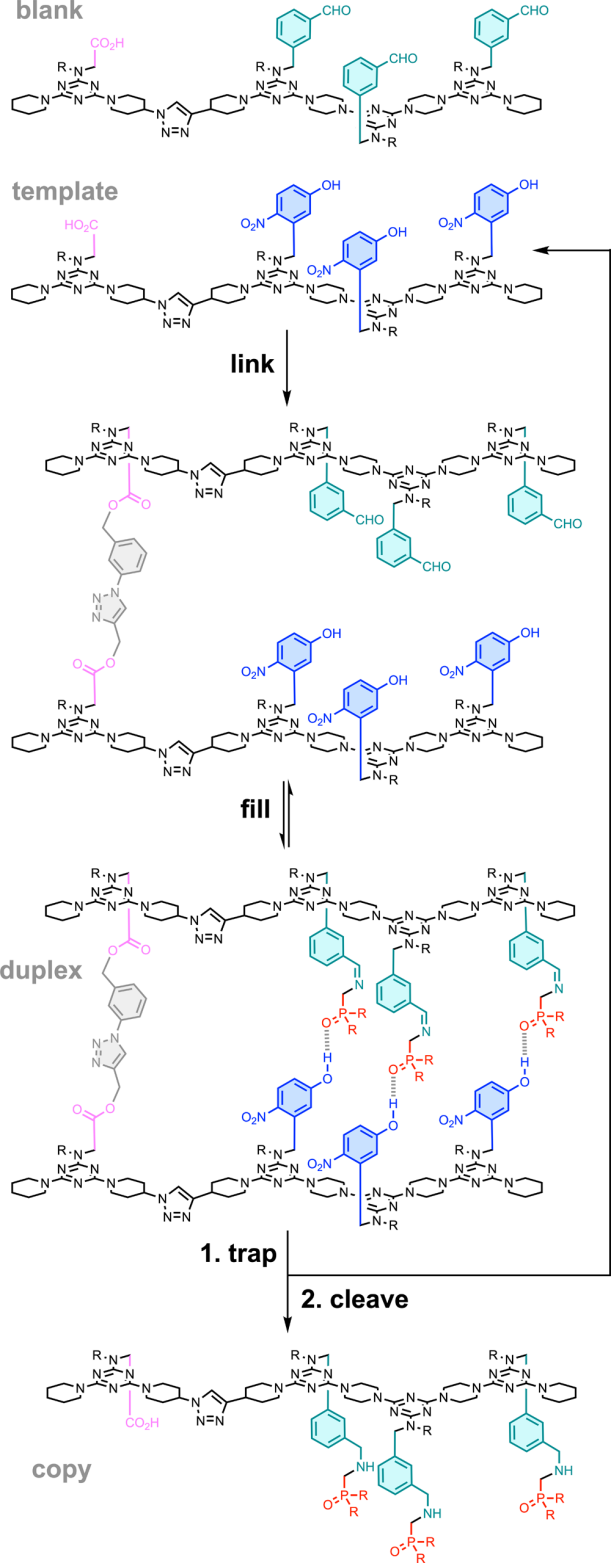
Sequence-selective template-directed REMO synthesis using base-filling. The trialdehyde blank strand was first covalently attached to the template using ester chemistry, and then dynamic imine chemistry was used to base-fill recognition units onto the copy strand. The resulting duplex was trapped and cleaved to yield the complementary copy and regenerate the template.

## Conclusions

Template-directed base-filling is an attractive alternative to templated polymerization for the replication of sequence information in synthetic polymers. Base-filling involves equilibration of recognition units onto a presynthesized blank strand using dynamic covalent chemistry, so that duplex formation via base-pairing interactions with complementary recognition units on a template strand directs the sequence of the resulting copy. Trapping the dynamic bonds on the copy strand completes the replication cycle. Here different strategies were investigated for achieving high fidelity base-filling replication of sequence information in recognition-encoded melamine oligomers (REMO).

A template with three 4-nitrophenol recognition units (**DDD**) was used with a trialdehyde blank strand, and imine chemistry was used for the dynamic attachment of recognition units. The aldehydes on the blank strand were allowed to compete for benzylamine or an amine equipped with a phosphine oxide recognition unit (**A**), which forms strong H-bonds with the complementary 4-nitrophenol recognition units on the template. Although there was some selectivity for the incorporation of the phosphine oxide into the copy strand, the maximum yield was 79% compared with 21% benzyl imine in dichloromethane solution.

NMR experiments indicate that at high concentrations the large excess of phosphine oxide recognition units present in solution compete with the intramolecular H-bonding interactions that should stabilize the duplex and lead to efficient templating. Reducing the concentrations leads to duplex dissociation, and all template effects are lost. However, it is possible to avoid duplex dissociation by covalently linking the blank strand to the template. The blank strand and the template strand were therefore both equipped with a terminal carboxylic acid group, so that they could be covalently connected via ester linkages and then separated again after the replication process by hydrolysis. In the covalently linked system, the selectivity of the base-filling process increased to 87% in dichloromethane solution.

Modeling of the various competing equilibria in the dynamic mixtures revealed that there two key parameters that limit the fidelity of replication by base-filling. The effective molarity for formation of intramolecular base-pairing interactions within the duplex (EM) sets an upper limit on the operating concentration. If the concentration of the recognition units exceeds the EM, then intermolecular interactions with the excess recognition units present in solution will denature the duplex and degrade any template effects. If the template and blank strand are covalently linked, then at low concentrations, the only limit on the fidelity of the copying process is fraying of the duplex. Any unpaired recognition units on the copy strand exchange with amines in solution in an uncontrolled manner, so selectivity depends on the probability of breaking intramolecular interactions in the duplex. This process is governed by the product *K*_h_ EM (where *K*_h_ is the association constant for formation of an intermolecular H-bonded base-pair), so higher affinity base-pairing interactions lead to higher fidelity base-filling. It was possible to increase *K*_h_ for the 4-nitrophenol•phosphine oxide interaction by using a less polar solvent. When the covalently linked base-filling experiments were repeated in toluene, the selectivity for incorporation of the phosphine oxide recognition unit in the copy strand increased to 92%.

These results demonstrate the potential of the base-filling strategy for replication of sequence information in synthetic polymers and delineate some of the conditions required to obtain high sequence fidelity. Base-filling offers an attractive alternative to replication by template-directed polymerization, because the macrocyclisation reactions that represent a major competing pathway in the polymerization process are not possible with the preassembled backbone that is used for base-filling. An advantage of using dynamic chemistry for template-directed base-filling of the copy strand is the error correction mechanism provided by equilibration. The disadvantage is that undesired products are in equilibrium with the sequence-complementary copy, so errors will be difficult to completely eliminate unless there is a very large thermodynamic driving force in favor of the desired product. While some errors may be useful in an evolutionary process that requires mutation to explore sequence space, an error rate that is too high will simply degrade any useful sequence information in the population.

The experiments described here show that the error rate is determined by the strength of the interaction between recognition units and the effective molarity for intramolecular base-pairing in the duplex. The fidelity of the copying process also depends on the length of the template, because the number of errors is proportional to the number of base-pairs. High fidelity templating of long oligomers can therefore be achieved if the error rate is reduced by increasing the strength of the base-pairing interaction (*K*_h_). For example, for an oligomer with 100 recognition units and an effective molarity of 10 mM, templating with error rates of less than 1% due to fraying of base-pairs would require a value of *K*_h_ greater than 5 × 10^5^ M^–1^. Sufficiently high affinity interactions can be achieved by using multiple base-pairing interactions to bind the recognition units to the template,^29^ or stronger base-pairing interactions such as salt bridges in place of H-bonds.^34^ The base-filling process must be carried out at concentrations sufficiently below the effective molarity to prevent partially denatured duplexes. Thus, a second requirement for a high fidelity copying process is that the dynamic chemistry used to attach the recognition units to the blank strands must be both rapid and quantitative at micromolar concentrations.

## Supplementary Material



## Notes

Materials and methods, detailed synthetic procedures, characterization including NMR spectra and HRMS of all compounds, imine equilibration studies, Musketeer speciation models, and DOSY experiments are available (PDF)
