# Supplementary material for: Template-Directed Synthesis of Recognition-Encoded Melamine Oligomers Using a Base-Filling Strategy
Source: J Am Chem Soc. 2025 May 15;147(21):18284–94. doi: 10.1021/jacs.5c05681 (PMC12123603; doi:10.1021/jacs.5c05681)
Supplement: Supplementary file 1 [file ja5c05681_si_001.pdf]

Template-Directed Synthesis of Recognition-Encoded Melamine Oligomers using a Base-Filling Strategy

Joseph T. Smith[1], Joaquin Baixeras Buye[1], Ben Iddon[1], Daniil O. Soloviev[1] and Christopher A. Hunter[1]

[1] Yusuf Hamied Department of Chemistry, University of Cambridge, Cambridge CB2 1EW, U.K.

Supplementary information

Contents

1. General experimental methods S2
2. Compound synthesis and characterisation S3
3. Determination of the relative imine stabilities S63
4. Procedure for the dynamic imine formation in DCM S65
5. Procedure for imine trapping and linker cleavage S67
6. Study of the re-equilibration of the imine mixture in toluene S68
7. Models of the linked and unlinked systems S69
8. DOSY NMR experiments S71
9. Analysis of the reduced and cleaved copy mixture S74
10. References S75

1. General experimental methods

All the reagents and materials were obtained from commercial sources and used without further purification. Dry solvents were taken from the solvent purification system Pure SolvTM by Innovative Technology, Inc.. Thin layer chromatography was carried out using Silica gel 60F on glass. Flash chromatography was carried out on an automated system (Combiflash Rf+ or Rf Lumen) using pre-packed cartridges of silica (25 µm PuriFlash Column). NMR spectra were recorded on a Bruker 400 MHz AVIII400, 400 MHz Neo Prodigy, 400 MHz QNP cryoprobe, 500 MHz TCI cryoprobe or 700 MHz TCO cryoprobe spectrometer. The residual solvent was used as the internal standard. In chloroform-*d*, ^{1}H spectra were referenced to δ 7.26 ppm and ^{13}C spectra to 77.16 ppm for the solvent signal. In dichloromethane-*d2*, ^{1}H spectra were referenced to δ 5.32 ppm and ^{13}C spectra to 53.84 ppm for the solvent signal. All chemical shifts are quoted in ppm on the δ scale. Splitting patterns are given as follows: s (singlet), br s (broad singlet), d (doublet), t (triplet), q (quartet), sept (septet), non (nonet) and m (multiplet). ^{1}H and ^{13}C NMR spectra were assigned using DEPT, COSY, HSQC and HMBC spectra. Psuedo-equivalent environments are labelled with the same number and where there are multiple peaks that are not fully resolved, from pseudo-equivalent environments or rotamers, peaks are grouped in square brackets. Where multiple peaks arise only from rotamers, this is specified. A Waters LCT premier mass spectrometer was used to obtain the ES+ mass spectra. FT-IR spectra were measured on a PerkinElmer Spectrum One spectrometer equipped with an ATR cell. ^{1}H DOSY NMR spectra were recorded using the standard Bruker pulse programme, stebpgp1s, employing a stimulated echo experiment using bipolar gradients, with a convection compensated sequence. DOSY NMR spectra were processed in MestReNova V 15.0.0 using the Peak Fit method. The LCMS analysis of samples was performed using Waters Acquity H-class UPLC coupled with a single quadrupole Waters SQD2. ACQUITY UPLC BEH C4 Column, 300 Å, 1.7 µm, 2.1 mm X 50 mm was used as the UPLC column. The conditions of the UPLC method are as follows. Solvent A: Water + 0.1% Formic acid; Solvent B: THF + 0.1% Formic acid; Gradient of 0-4 minutes 30% - 100% B then 2 minutes 100% B with re-equilibration time of 2 minutes. Flow rate: 0.4 ml/min; Column temperature of 40 °C; Injection volume of 2 µL. The signal was monitored at 254 nm.

2. Compound synthesis and characterisation

Synthesis of 1

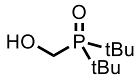

Compound **1** was synthesises according to the literature procedure.[1]

Synthesis of 2

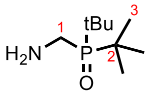

To a solution of **1** (1.0 g, 5.2 mmol, 1 eq.) and triethylamine (1.1 mL, 7.8 mmol, 1.5 eq.) in DCM (20 mL) at 0 °C, mesyl chloride (604 µL, 7.8 mmol, 1.5 eq.) was added dropwise and the reaction was stirred at room temperature overnight. The solution was diluted with DCM (20 mL) and washed with water (2 x 40 mL) and brine (40 mL). The organic phase was dried over magnesium sulphate and the solvent was removed under reduced pressure. The obtained residue was used in the next step without further purification.
The crude from the previous step was dissolved in 4-methoxybenzylamine (4.8 mL, 36 mmol, 7 eq.) and the solution was heated at 120 °C for 2.5 hours under microwave irradiation and a nitrogen atmosphere. The solution was diluted with ethyl acetate (50 mL) and washed with water (100 mL). The organic layer was dried over magnesium sulphate and the solvent removed under reduced pressure. The obtained residue was purified by silica flash chromatography (DCM / methanol (95:5)).
The crude from the previous step was dissolved in methanol (25 mL) and palladium on carbon (553 mg, 5.2 mmol, 1 eq.) was added. The suspension was stirred at room temperature for 1 day under an atmosphere of hydrogen. The suspension was filtered and the obtained filtrate was concentrated under reduced pressure. The obtained residue was redissolved in methanol (5 mL) and formic acid (294 µL, 7.8 mmol, 1.5 eq.) was added. The solution was loaded onto SCX resin and the resin was washed with methanol (100 mL). The resin was then washed with methanolic ammonia (7 N, 100 mL) and the solvent was removed under reduced pressure. **2** was obtained as a pale yellow oil (546 mg, 2.85 mmol, 55%).

^{1}H NMR (400 MHz, $CDCl_3$): δ_H = 3.03 (d, $^2J_{HP}$ = 2.9 Hz, 2H, H_1), 1.22 (d, $^3J_{HP}$ = 13.0 Hz, 18H, H_3).

^{13}C NMR (101 MHz, $CDCl_3$): δ_C = 35.4 (d, $^1J_{CP}$ = 56.1 Hz, C_2), 34.06 (d, $^1J_{CP}$ = 55.0 Hz, C_1), 26.57 (C_3).

^{31}P NMR (162 MHz, $CDCl_3$): δ_P = 57.0.

HRMS (ES+): Calculated for $C_9H_{23}NOP^+$, 192.1517; found 192.1514.

FT-IR (ATR): ν_{max} 3383 (br), 2951, 2904, 2873, 1642, 1477, 1393, 1370, 1205, 1134, 935, 820, 708, 644, 503, 455, 418.

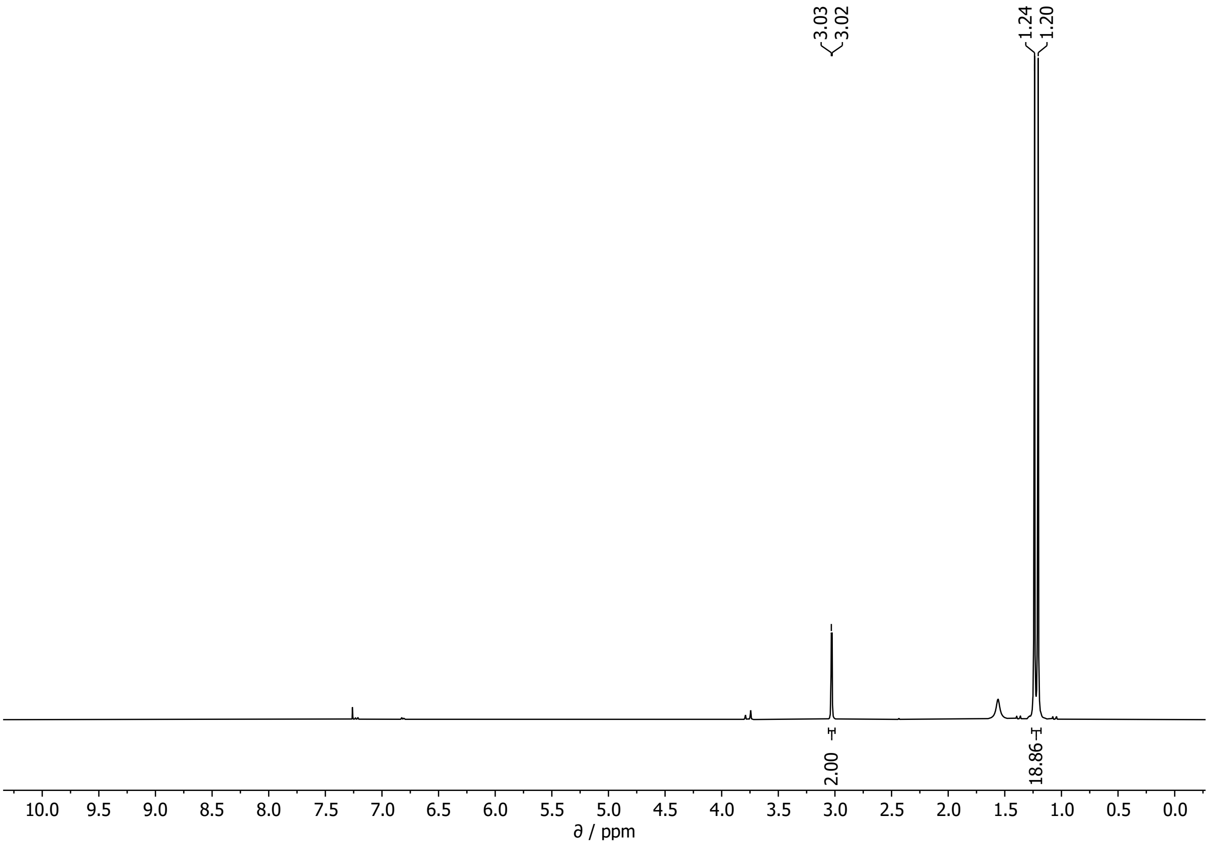

Figure S1 ^{1}H NMR spectrum (400 MHz, $CDCl_3$, 298 K) of **2**.

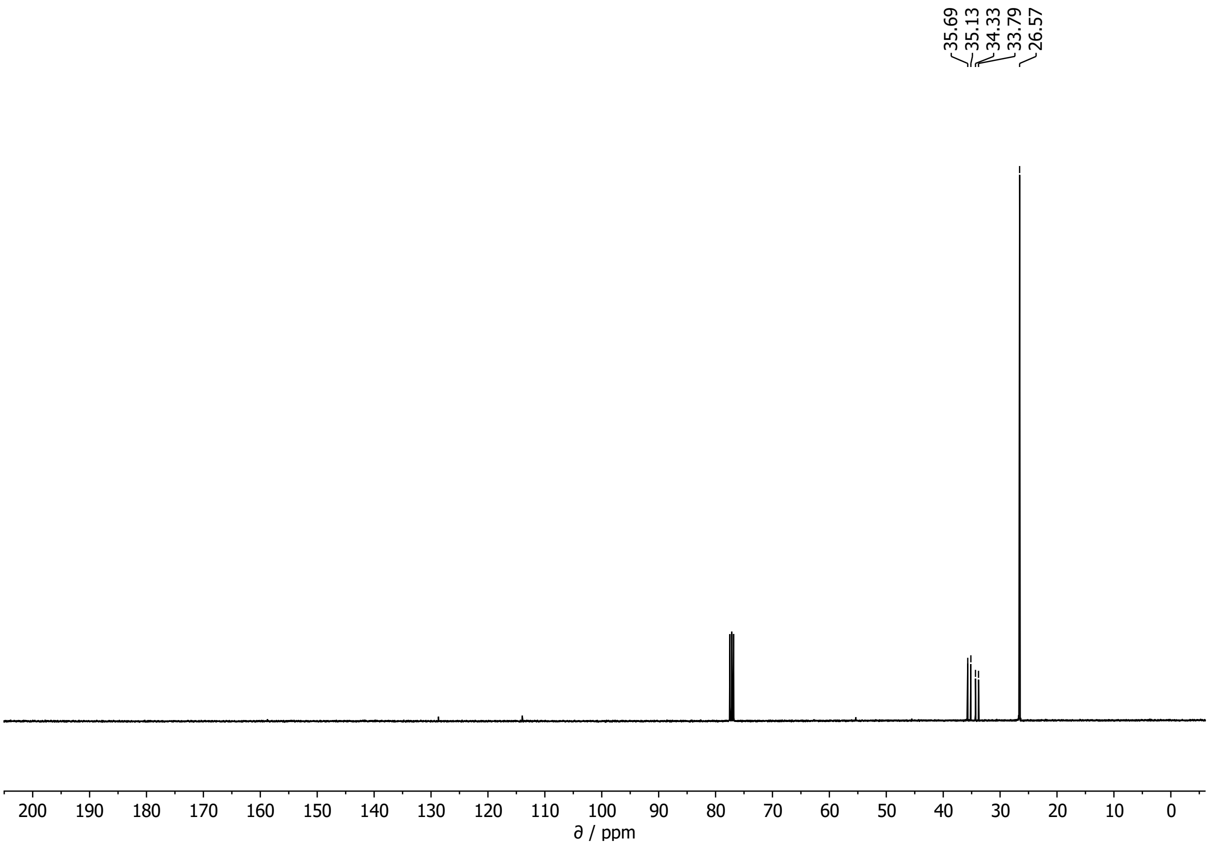

Figure S2 ^{13}C NMR spectrum (101 MHz, $CDCl_3$, 298 K) of **2**.

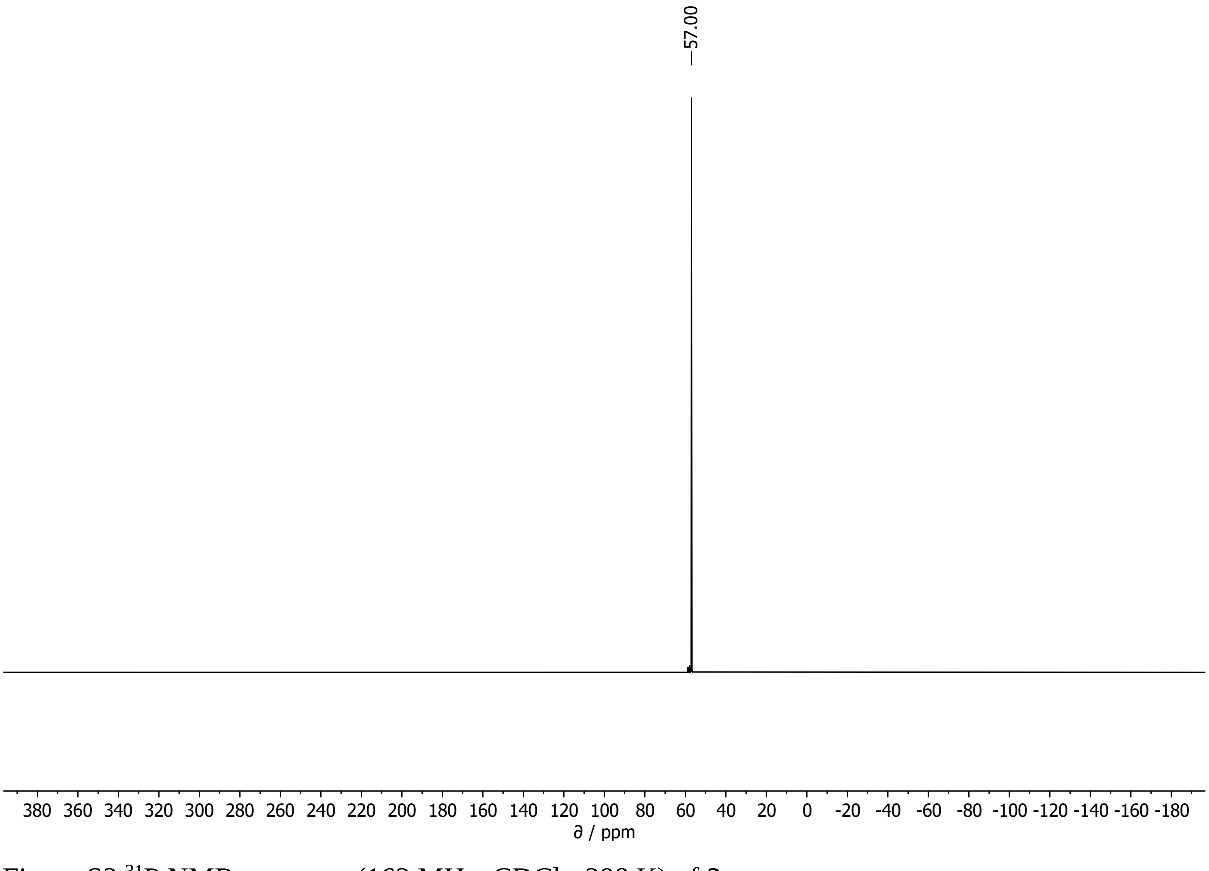

Figure S3 ^{31}P NMR spectrum (162 MHz, $CDCl_3$, 298 K) of **2**.

Synthesis of 3

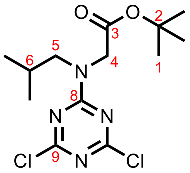

Tert-butyl aminoacetate hydrochloride (5.0 g, 30 mmol, 1.0 eq.), isobutanal (3.0 mL, 33 mmol, 1.1 eq.) and DIPEA (6.0 mL, 35 mmol, 1.2 eq.) were dissolved in dry DCM (80 mL) and stirred at room temperature for one hour. The solution was cooled to 0 °C and sodium borohydride (2.0 g, 54 mmol, 1.8 eq.) and methanol (10 mL) were added then the solution was stirred at room temperature for 15 hours. The solution was cooled to –78 °C then cyanuric chloride (5.7 g, 31.3 mmol, 1.04 eq.) was added quickly and the solution was stirred for one hour. The solvent was removed *in vacuo* and the residues were dissolved in EtOAc (100 mL). The solution was washed with NaOH (1M in water, 3 × 50 mL) and HCl (0.1 M in water, 3 × 50 mL). The combined aqueous phases were extracted with EtOAc (50 mL) then the combined organic phases were dried over magnesium sulphate and the solvent was removed *in vacuo*. The crude was purified by three rounds of recrystallisation from toluene at – 20 °C and the crystals were washed with toluene at -78 °C. **3** was obtained as white crystals (5.69 g, 17.0 mmol, 57%).

^{1}H NMR (400 MHz, $CDCl_3$): δ_H 4.13 (s, 2H, H_4), 3.49 (d, J = 7.4 Hz, 2H, H_5), 2.01 (non, *J* = 6.9 Hz, 1H, H_6), 1.47 (s, 9H, H_1), 0.95 (d, J = 6.7 Hz, 6H, H_7).

^{13}C NMR (101 MHz, $CDCl_3$): δ_C 169.9 (C_9), 167.3 (C_8), 165.9 (C_3), 82.8 (C_2), 56.6 (C_5), 51.1 (C_4), 28.2 (C_1), 27.4 (C_6), 20.2 (C_7).

HRMS (ES+): Calculated for. for $C_{13}H_{21}Cl_2N_4O_2^+$, 335.1036; found 335.1044.

FT-IR (ATR): ν_{max} /cm^{-1} 2962, 1744, 1567, 1483, 1368, 1328, 1226, 1149, 1078, 977, 847, 798.

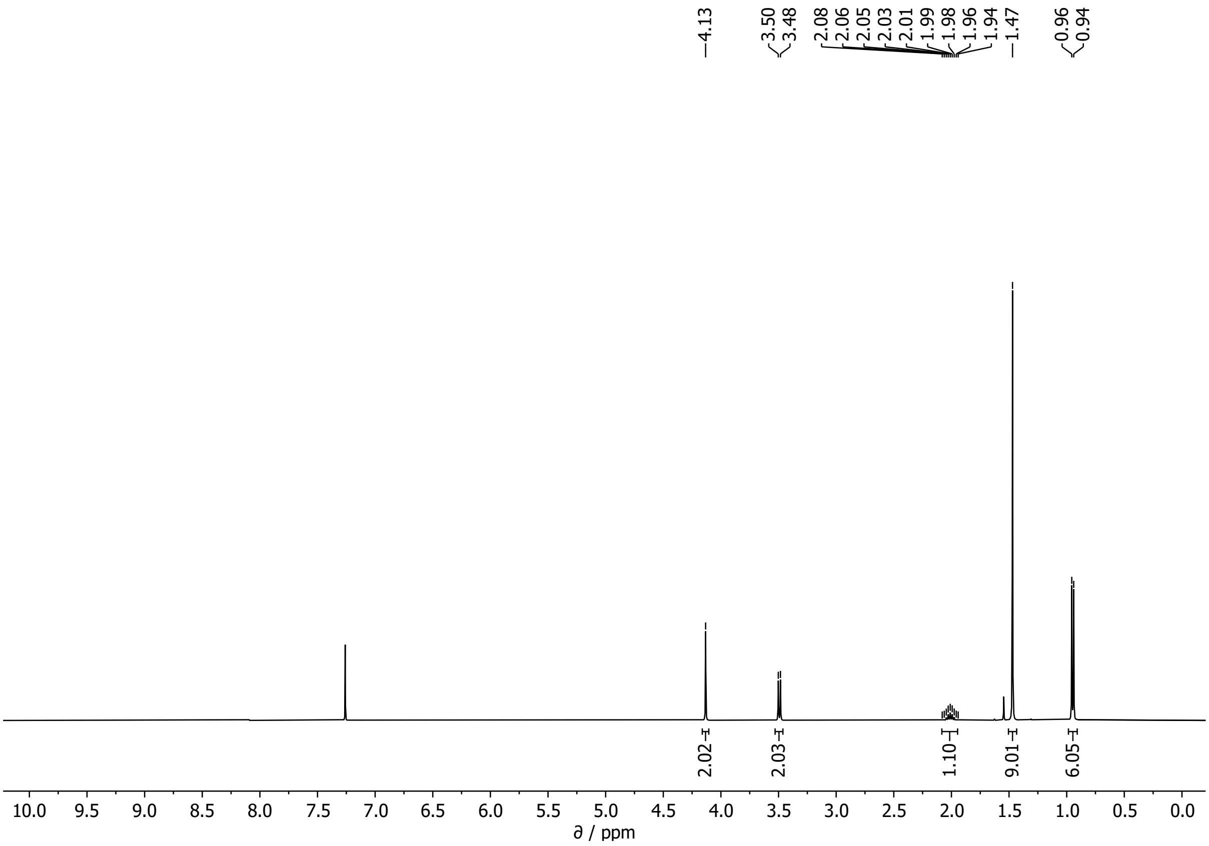

Figure S4 ^{1}H NMR spectrum (400 MHz, $CDCl_3$, 298 K) of **3**.

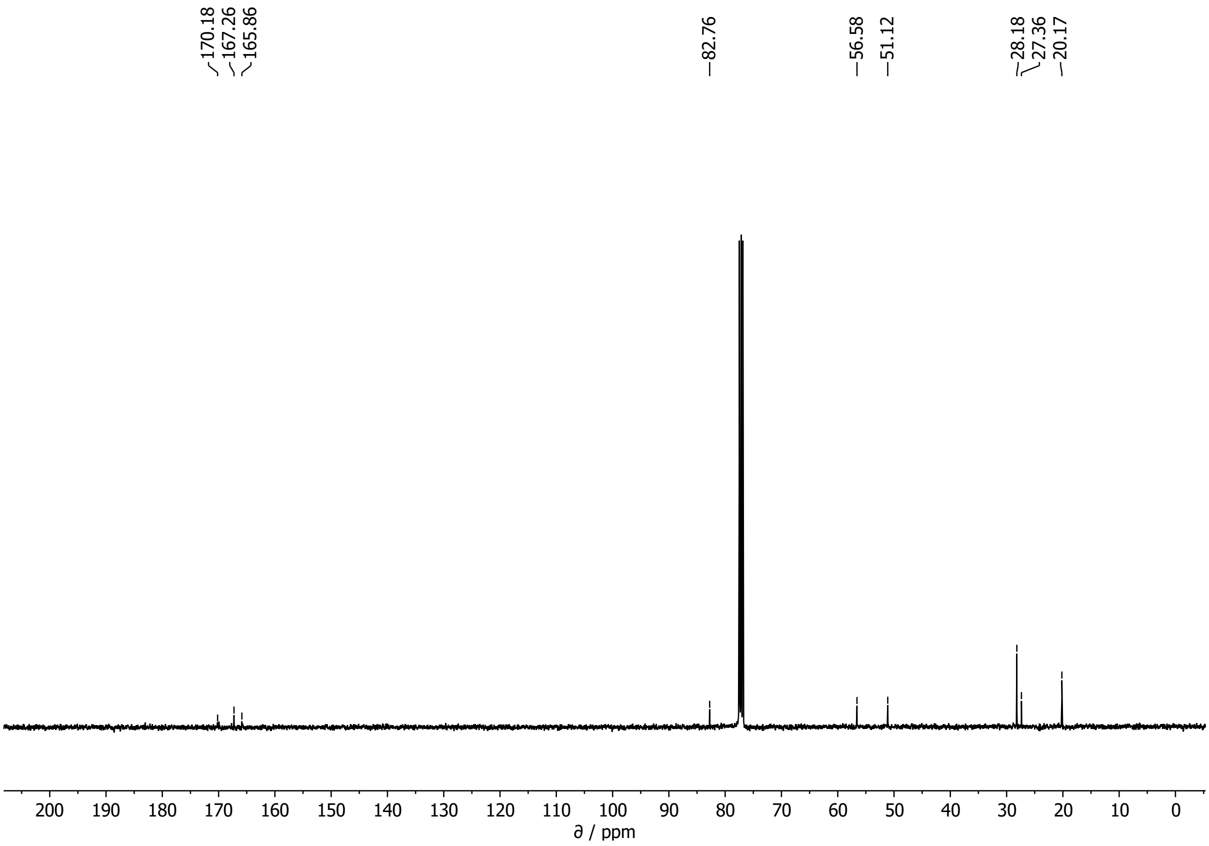

Figure S5 ^{13}C NMR spectrum (101 MHz, $CDCl_3$, 298 K) of **3**.

Synthesis of 4

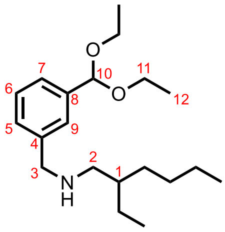

To a solution of 3-(diethoxymethyl)benzaldehyde (2.5 mL, 0.012 mol, 1 eq.) in DCM (50 mL), 2-ethylhexylamine (2.4 mL, 0.015 mol, 1.2 eq.) and molecular sieves (4 Å) were added and the reaction was stirred overnight at room temperature. The reaction mixture was cooled to 0 °C and diluted with methanol (50 mL). Sodium borohydride (570 mg, 0.015 mol, 1.2 eq.) was added and the mixture was stirred for 2 hours. The solvent was removed under reduced pressure and the residue was redissolved in ethyl acetate (50 mL). The organic phase was washed with saturated sodium hydrogen carbonate solution (50 mL), and the aqueous phase was extracted with ethyl acetate (3 x 50 mL). The combined organic phases were dried with magnesium sulphate and the solvent was removed under reduced pressure. The residues obtained were purified by silica flash chromatography (pet ether/ ethyl acetate (8:2)). **4** was obtained as a colourless oil (3.29 g, 0.010 mol, 85%).

^{1}H NMR (700 MHz, $CDCl_3$): δ_H = 7.41 (s, 1H, H_9), 7.35 (d, J = 7.5 Hz, 1H, H_7), 7.31 (t, J = 7.5 Hz, 1H, H_6), 7.28 (d, J = 7.4 Hz, 1H, H_5), 5.49 (s, 1H, H_{10}), 3.79 (s, 2H, H_3), 3.62 (dq, J = 9.7, 7.1 Hz, 2H, H_{11}), 3.54 (dq, J = 9.6, 7.1 Hz, 2H, H_{11}), 2.51 (d, J = 6.2 Hz, 2H, H_2), 1.43 (hept, J = 6.0 Hz, 1H, H_1), 1.40 – 1.25 (m, 8H, CH_2 of alkyl chain), 1.24 (t, J = 7.0 Hz, 6H, H_{12}), 0.88 (t, J = 7.1 Hz, 3H, CH_3 of alkyl chain), 0.84 (t, J = 7.4 Hz, 3H, CH_3 of alkyl chain).
^{13}C NMR (176 MHz, $CDCl_3$): δ_C = 140.9 (C_4), 139.2 (C_8), 128.3 (C_6), 128.2 (C_5), 126.4 (C_9), 125.2 (C_7), 101.8 (C_{10}), 61.2 (C_{11}), 54.3 (C_3), 52.7 (C_2), 39.6 (C_1), 31.5, 29.1, 24.6, 23.3 (CH_2 carbons of alkyl chain), 15.3 (C_{12}), 14.3, 11.0 (methyl carbons of alkyl chain).
HRMS (ES+): Calculated for $C_{20}H_{36}NO_2^+$, 322.2741; found 322.2763.
FT-IR (ATR): ν_{max} 2959, 2926, 2872, 1457, 1443, 1371, 1333, 1158, 1113, 1096, 1053, 1003, 900, 784, 764, 728, 705.

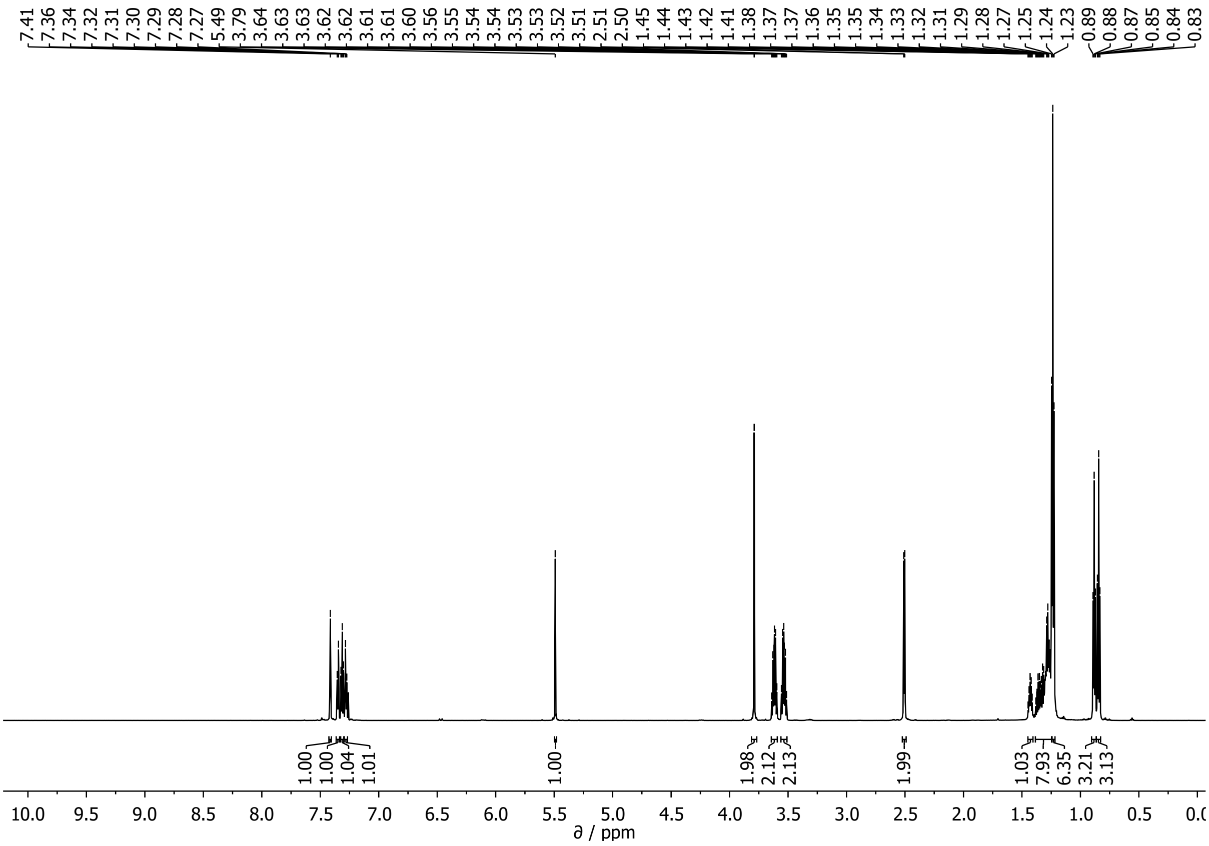

Figure S6 ^{1}H NMR spectrum (700 MHz, $CDCl_3$, 298 K) of **4**.

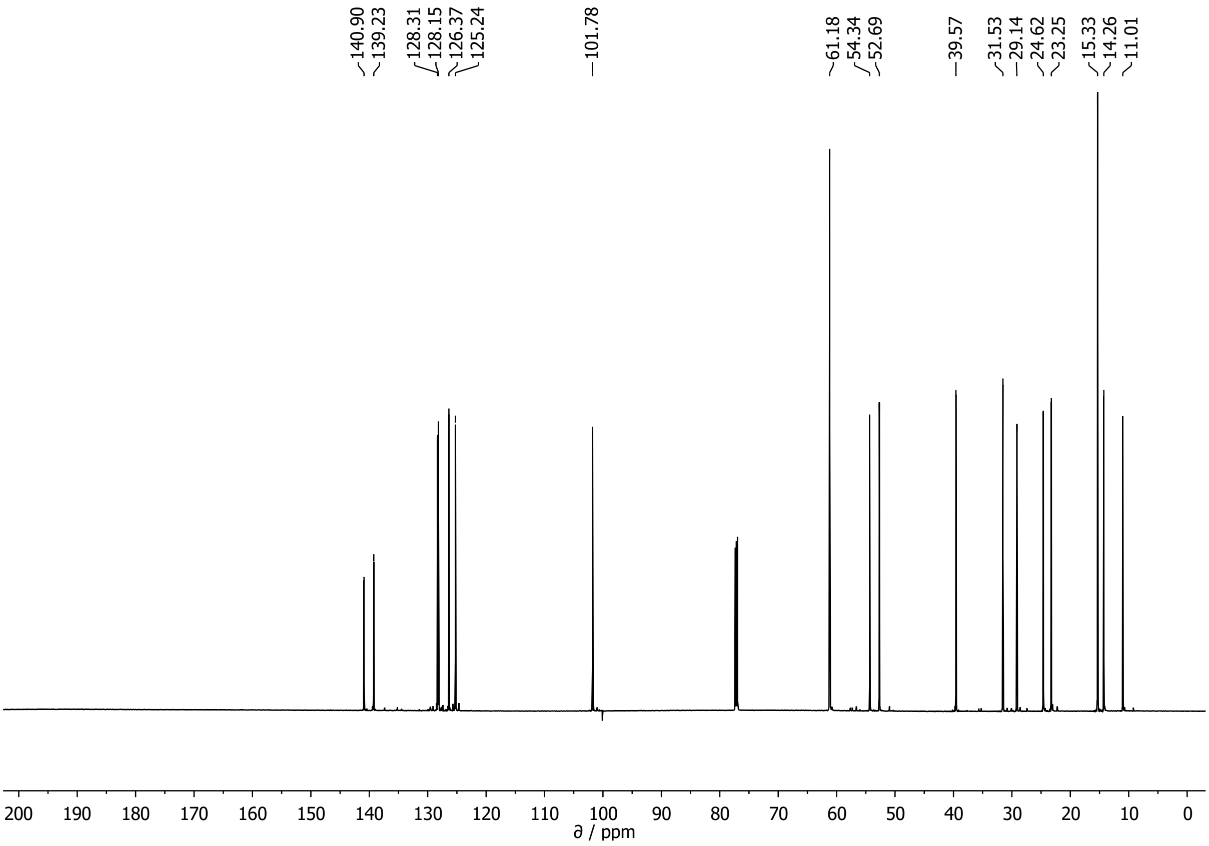

Figure S7 ^{13}C NMR spectrum (176 MHz, $CDCl_3$, 298 K) of **4**.

Synthesis of 5

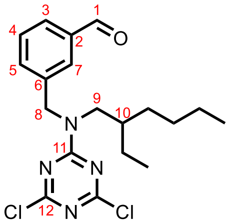

To a solution of cyanuric chloride (1.98 g, 10.7 mmol, 1.05 eq) in THF (28 mL) at -10 °C, a solution of **4** (3.29 g, 10.2 mmol, 1.0 eq.) in THF (6 mL) and DIPEA (7.1 mL, 41 mmol, 4 eq.) was added dropwise. The reaction mixture was stirred at -10 °C for 2 hours. The reaction was filtered and the solvent was removed under reduced pressure. The obtained residue was dissolved in ethyl acetate (50 mL) and washed with citric acid solution (3 x 50 mL). The organic phase was dried with magnesium sulfate and the solvent was removed *in vacuo*. The obtained residue was dissolved in DCM (50 mL). HCl (3 M, 50 mL) was added and the suspension stirred vigorously for 1 hour. The organic phase was separated and washed with saturated sodium hydrogen carbonate solution (50 mL), water (50 mL) and brine (50 mL). The organic phase was dried with magnesium sulfate and the solvent was removed *in vacuo*. The residues obtained were purified by silica flash chromatography (pet ether/ ethyl acetate (95:5)). **5** was obtained as a colourless oil (4.03 g, 10.2 mmol, quantitative).

^{1}H NMR (700 MHz, CDCl$_3$): δ_H = 10.01 (s, 1H, H_1), 7.82 (dt, J = 7.3, 1.6 Hz, 1H, H_3), 7.73 (s, 1H, H_7), 7.53 (t, J = 7.4 Hz, 1H, H_4), 7.51 (dt, J = 7.7, 1.7 Hz, 1H, H_5), 4.94 (d, J = 15.4 Hz, 1H, H_8), 4.90 (d, J = 15.4 Hz, 1H, H_8), 3.51 (d, J = 7.4 Hz, 2H, H_9), 1.80 (hept, J = 7.1 Hz, 1H, H_{10}), 1.46 – 1.11 (br, 8H, CH_2 protons of alkyl chain), 0.97 – 0.80 (br, 6H, methyl protons of alkyl chain).

^{13}C NMR (176 MHz, CDCl$_3$): δ_C = 192.0 (C_1), 170.4 and 170.3 (rotamers, C_{12}), 165.8 (C_{11}), 137.3 (C_6), 137.0 (C_2), 133.8 (C_5), 129.8 (C_3), 129.8 (C_4), 128.5 (C_7), 50.7 (C_9), 50.4 (C_8), 37.4 (C_{10}), 30.4, 28.5, 23.8, 23.1 (CH_2 carbons of alkyl chain), 14.2, 10.7 (methyl carbons of alkyl chain).

HRMS (ES+): Calculated for $C_{19}H_{25}Cl_2N_4O^+$, 395.1405; found 395.1412.

FT-IR (ATR): ν_{max} 2959, 2930, 2872, 2860, 1738, 1704, 1563, 1478, 1441, 1381, 1352, 1328, 1234, 1184, 1160, 1067, 974, 845, 796.

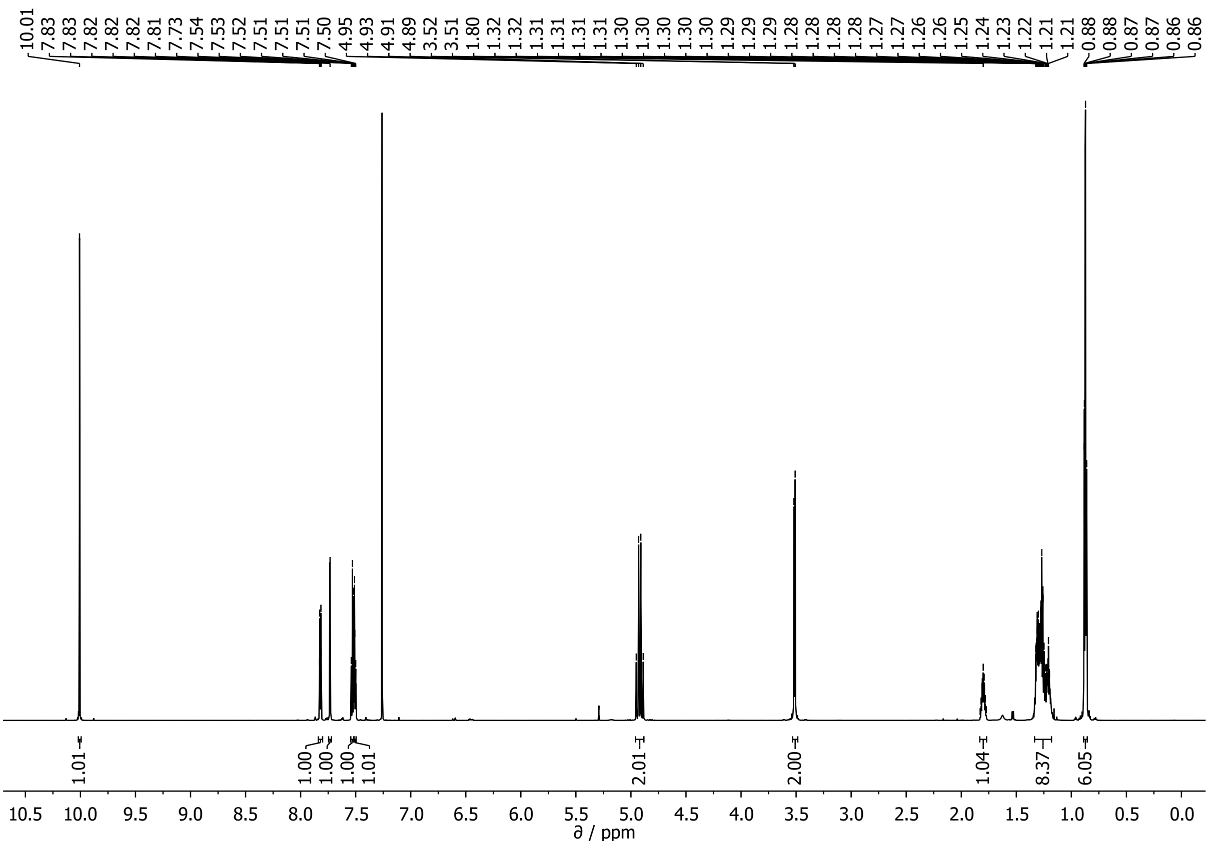

Figure S8 ^{1}H NMR spectrum (700 MHz, $CDCl_3$, 298 K) of **5**.

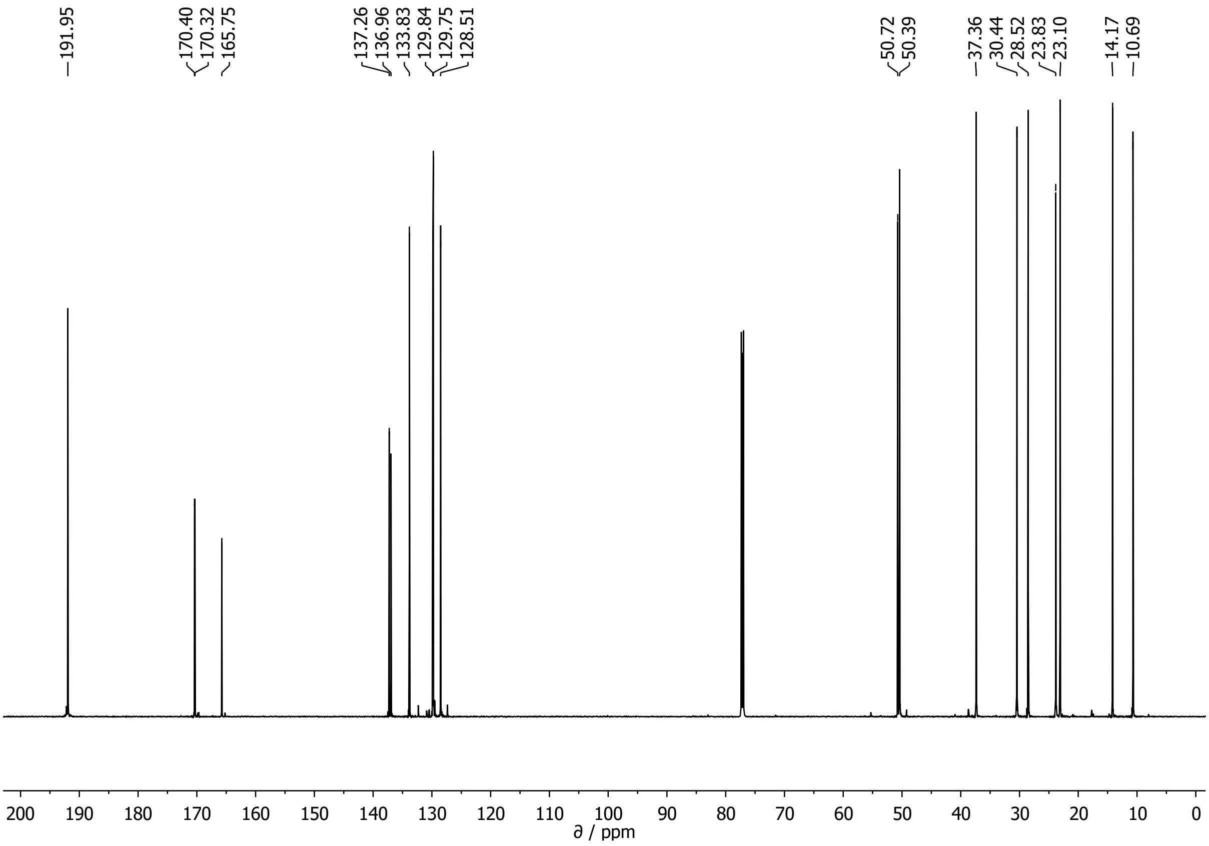

Figure S9 ^{13}C NMR spectrum (176 MHz, $CDCl_3$, 298 K) of **5**.

Synthesis of 6

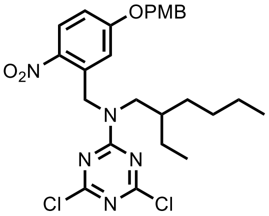

Compound **6** was synthesised according to the literature procedure.[2]

Synthesis of 7a

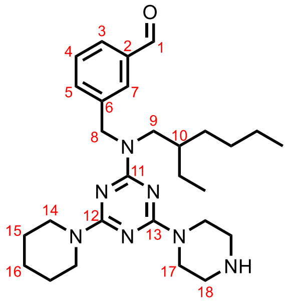

To a solution of **5** (750 mg, 1.90 mmol, 1.0 eq.) in DMF (20 mL), piperidine (187 µL, 1.90 mmol, 1.0 eq.) and potassium carbonate (525 mg, 3.8 mmol, 2 eq.) were added and the solution was stirred at room temperature for 2 hours. The solution was diluted with DMF (170 mL) and piperazine (11.5 g, 133 mmol, 70 eq.) was added. The solution was stirred at 90 °C overnight then cooled to room temperature. The solution was filtered, and the solvent was removed under reduced pressure. The obtained residue was dissolved in ethyl acetate (50 mL) and washed with water (2 x 50 mL) and 5% lithium chloride solution (50 mL). The organic phase was dried with magnesium sulphate and the solvent was removed under reduced pressure. The residues obtained were purified by silica flash chromatography (DCM/ methanol (9:1)). **7a** was obtained as a white foam (558 mg, 1.13 mmol, 59%).

^{1}H NMR (700 MHz, $CDCl_3$): δ_H = 9.98 (s, 1H, H_1), 7.76 – 7.69 (br, 2H, $H_{3,7}$), 7.72 (s, 1H), 7.56 – 7.45 (br, 1H, H_5), 7.43 and 7.39 (rotamers, t, J = 7.6 Hz, 1H, H_4), 4.90 – 4.77 (br, 2H, H_8), 4.01 and 3.91 (rotamers, br, 2H, H_{17}), 3.77 – 3.55 (br, 6H, $H_{14,17}$), 3.49 – 3.34 (br, 2H, H_9), 3.11 and 3.02 (br, 4H, H_{18}), 2.40 (br s, 1H, NH), 1.84 – 1.68 (br, 1H, H_{10}), 1.67 – 1.57 (br, 2H, H_{16}), 1.57 – 1.39 (br, 4H, H_{15}), 1.37 – 1.08 (br, 8H, CH_2 protons of alkyl chain), 0.87 – 0.77 (br, 6H, methyl protons of alkyl chain).

^{13}C NMR (176 MHz, $CDCl_3$): δ_C = [192.5 and 192.5 (rotamers, C_1)], 166.3 (C_{11}), [165.4, 165.4, 165.0, 164.9 ($C_{12/13}$)], 141.0 (C_6), [136.6 and 136.5 (rotamers, C_2)], 133.7 and 133.5

(rotamers, C_5)], [129.1 and 129.1 (rotamers, C_4)], [128.8 and 128.7 (rotamers, C_7)], [128.4 and 128.2 (rotamers, C_3)], [50.3 and 50.2 (rotamers, C_9)], [50.2 and 49.8 (rotamers, C_8)], [44.3 and 44.2 (rotamers, C_{14})], [44.2, 44.1, 44.0 (rotamers, C_{18})], 43.3 (rotamer, C_{14}), 41.4 (C_{17}), [38.1, 38.0, 38.0 (rotamers, C_{10})], [30.8, 29.0 (CH_2 carbons of alkyl chain), [25.9 and 25.8 (rotamers, C_{15})], [25.1 and 25.0 (rotamers, C_{16})], [23.9, 23.3 (CH_2 carbons of alkyl chain)], [14.2, 10.9, 10.8 (methyl carbons of alkyl chain)].

HRMS (ES+): Calculated for $C_{28}H_{44}N_7O^+$, 494.3607; found 494.3598.

FT-IR (ATR): ν_{max} 3300 (br), 2954, 2928, 2853, 1701, 1532, 1485, 1439, 1372, 1349, 1300, 1271, 1252, 1237, 1192, 1137, 1101, 1025, 997, 853, 807.

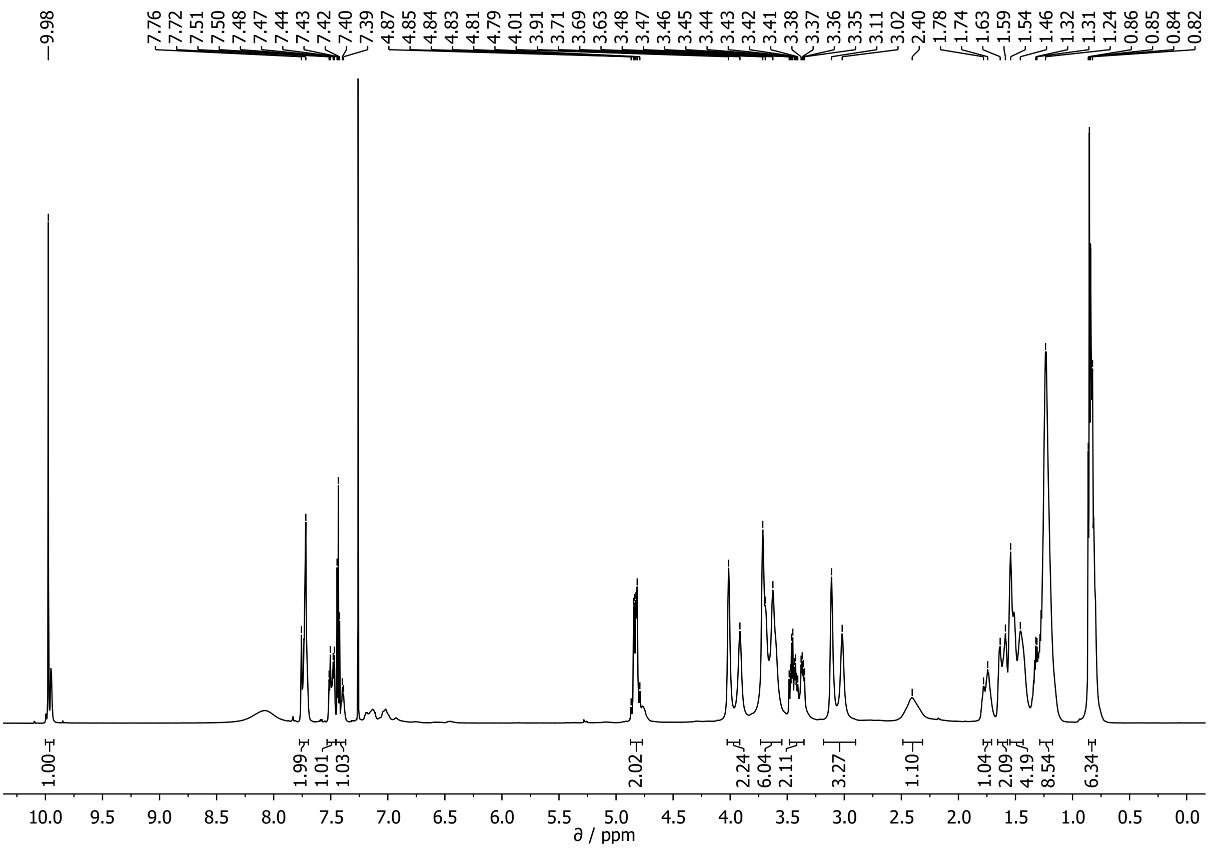

Figure S10 ^{1}H NMR spectrum (700 MHz, $CDCl_3$, 298 K) of **7a**.

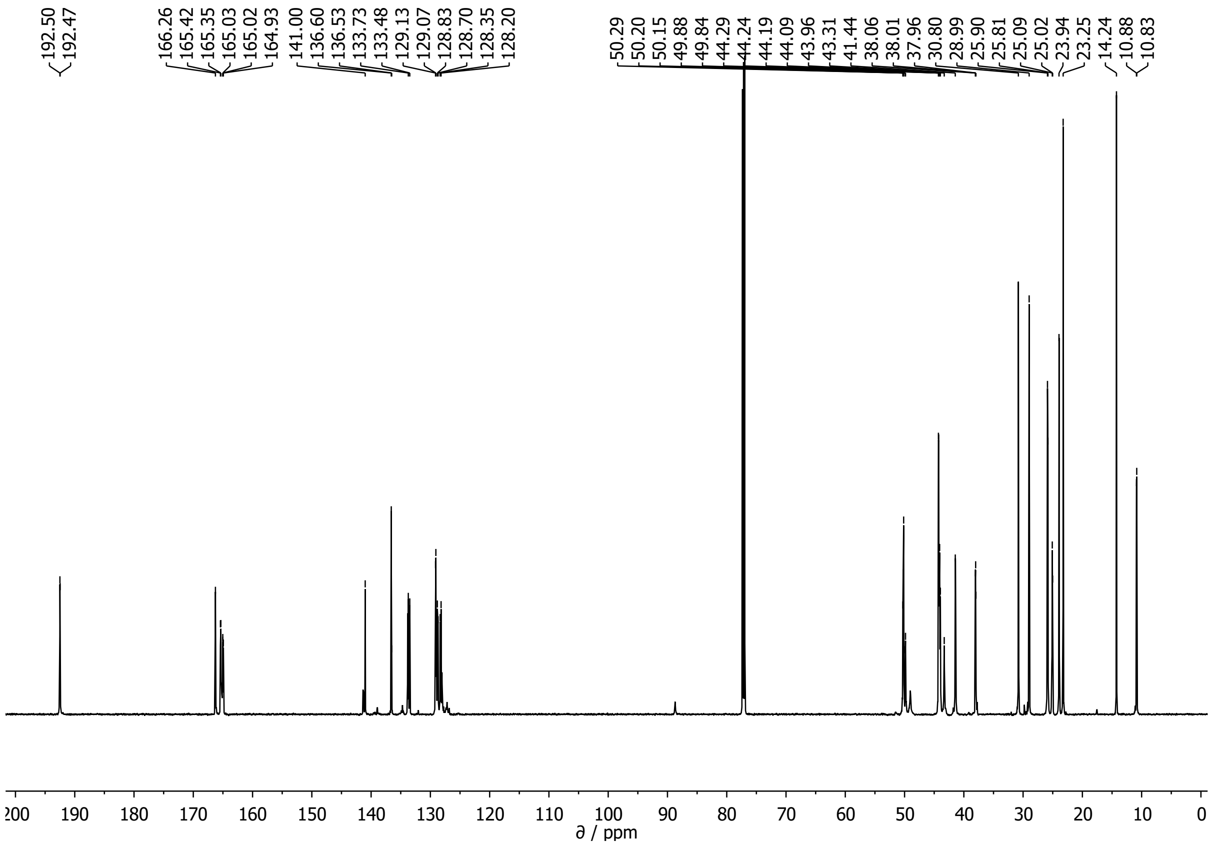

Figure S11 ^{13}C NMR spectrum (176 MHz, $CDCl_3$, 298 K) of **7a**.

Synthesis of 7b

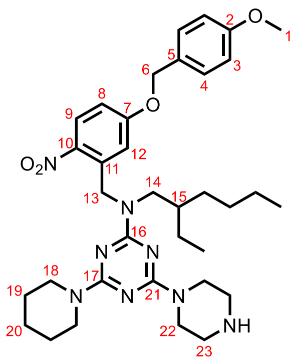

To a solution of **6** (5.00 g, 9.12 mmol, 1 eq.) in DMF (30 mL), piperidine (0.91 mL, 9.21 mmol, 1.01 eq.) and potassium carbonate (2.52 g, 18.2 mmol, 2 eq.) were added and the solution was stirred at room temperature for 2 hours. The solution was diluted with DMF (470 mL) and piperazine (30.1 g, 350 mmol, 38 eq.) was added. The solution was stirred at 90 °C overnight then cooled to room temperature. The solution was filtered, and the solvent was removed under reduced pressure. The obtained residue was dissolved in ethyl acetate (200 mL) and washed with water (2 x 200 mL) and 5% lithium chloride solution (200 mL). The organic phase was dried with magnesium sulphate and the solvent was removed under reduced pressure. The residues obtained were purified by silica flash chromatography (DCM/ methanol (20:1)). **7b** was obtained as a white foam (4.10 g, 6.34 mmol, 70%).

^{1}H NMR (700 MHz, CDCl$_3$): δ_H = 8.09 (d, J = 8.9 Hz, 1H, H_9), 7.28 (d, J = 8.2 Hz, 2H, H_4), 6.88 (d, J = 8.4 Hz, 2H, H_3), 6.84 (d, J = 9.2 Hz, 1H, H_8), 6.78 and 6.76 (s, 1H, rotamers, H_{12}), 5.10 (d, J = 17.8 Hz, 1H, H_{13}), 5.07 (d, J = 17.8 Hz, 1H, H_{13}), 4.97 (s, 2H, H_6), 3.80 (s, 3H, H_1), 3.79 and 3.75 (br, 4H, H_{22}), 3.54 (br, 4H, H_{18}), 3.41 (br, 2H, H_{14}), 2.90 (s, 2H, H_{23}), 2.75 (br s, 2H, H_{23}), 2.20 (br s, 1H, NH), 1.78 (br, 1H, H_{15}), 1.68 – 1.50 (br, 4H, H_{19}), 1.39 (br, 2H, H_{20}), 1.38 – 1.16 (m, 8H, CH_2 of alkyl chain), 0.97 – 0.78 (br, 6H, methyl groups of alkyl chain).

^{13}C NMR (176 MHz, CDCl$_3$): δ_C = 166.2 (C_{16}), [165.5, 165.4, 165.2, 165.0 ($C_{17/21}$)], 162.9 (C_7), 159.8 (C_2), 141.4 (C_{10}), 139.7 (C_{11}), 129.6 (C_4), 127.8 (C_5), 127.7 (C_9), 114.3 (C_{12}), 114.2 (C_3), 112.9 and 112.8 (rotamers, C_8), 70.4 (C_6), 55.4 (C_1), 51.0 (C_{14}), 48.9 (C_{13}), 46.1 and 45.9 (rotamers, C_{23}), 44.3 and 44.3 (rotamers, C_{22}), 44.1 (C_{18}), 38.3 and 38.3 (rotamers, C_{15}), 30.8, 29.0 (carbons of CH_2 chain), 26.0 (rotamer, C_{19}), 25.8 (C_{20}), 25.2 and 25.1 (rotamers, C_{19}), 24.0, 23.3 (carbons of CH_2 chain), 14.3, 10.9 (methyl carbons of chain).

HRMS (ES+): Calculated for $C_{35}H_{51}N_8O_4^+$, 647.4028; found 647.4015.

FT-IR (ATR): ν_{max} 3322 (broad), 2953, 2930, 2854, 1612, 1578, 1531, 1515, 1483, 1440, 1371, 1338, 1315, 1285, 1250, 1229, 1195, 1175, 1101, 1072, 1025, 999, 843, 807, 732.

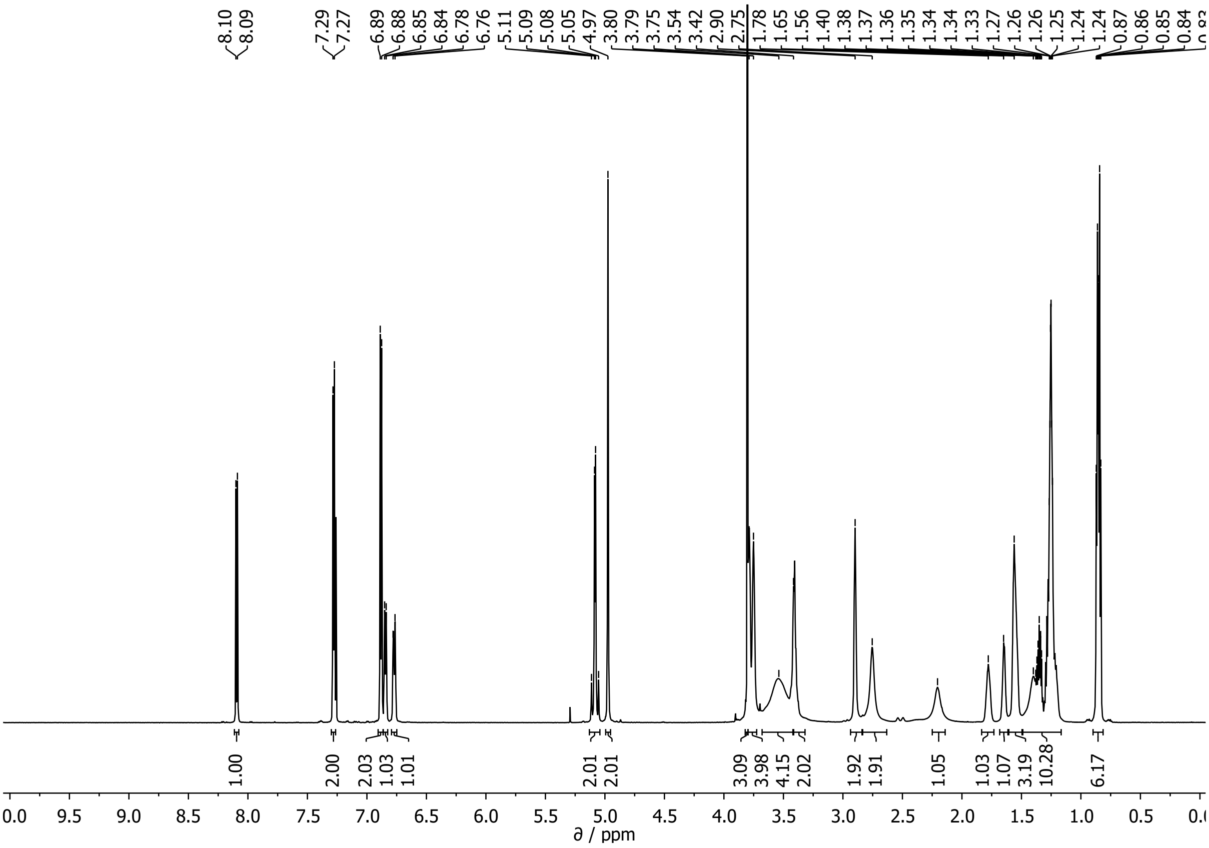

Figure S12 ^{1}H NMR spectrum (700 MHz, $CDCl_3$, 298 K) of **7b**.

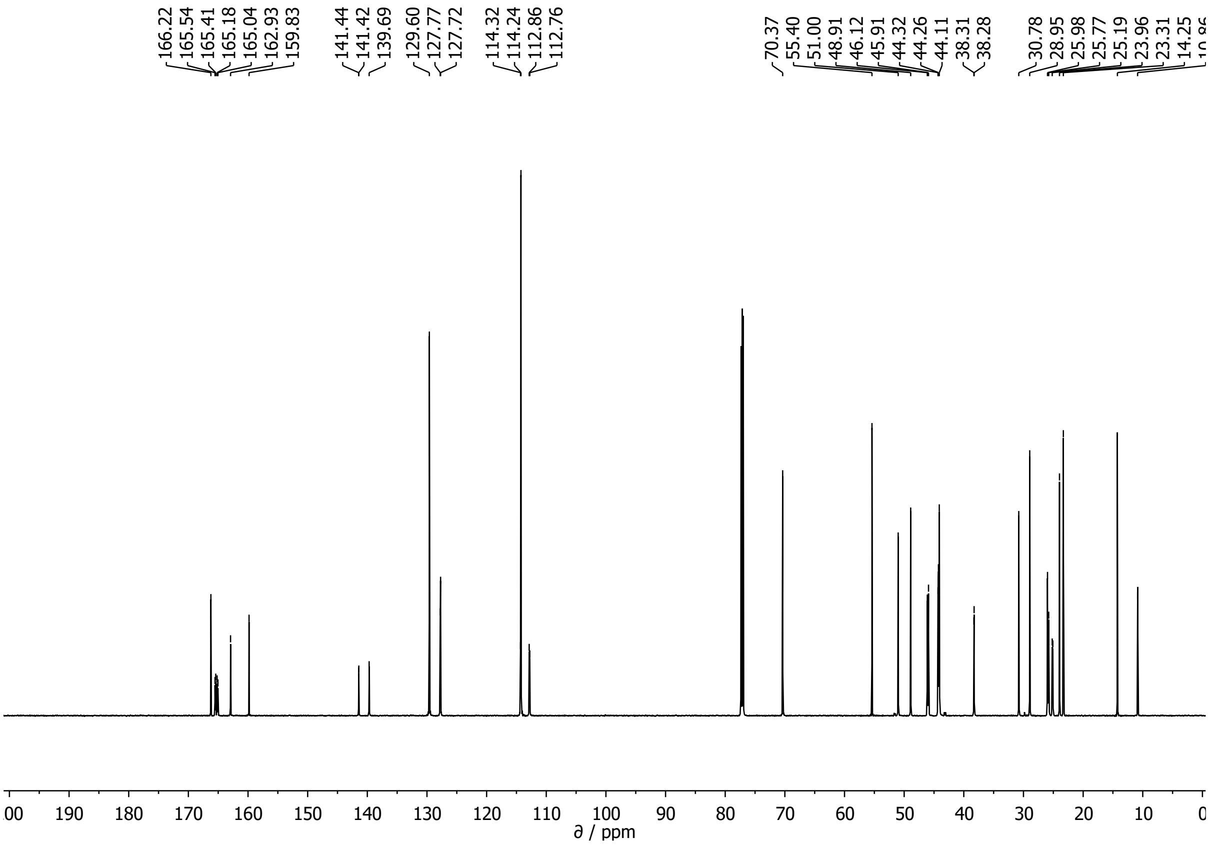

Figure S13 ^{13}C NMR spectrum (176 MHz, $CDCl_3$, 298 K) of **7b**.

Synthesis of 8a

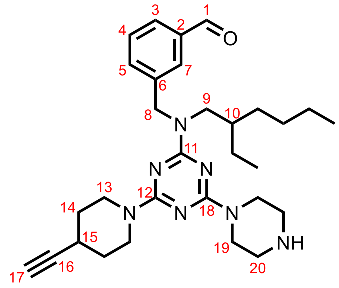

To a solution of *tert*-butyl-4-ethynylpiperidine-1-carboxylate (79.4 mg, 0.379 mmol, 1.0 eq.) in DCM (3 mL), trifluoroacetic acid (0.9 mL) was added and the solution was stirred for 2 hours. The solvent was removed under a nitrogen flow and the salt was redissolved in DMF (1 mL). To a solution of **5** (150 mg, 0.379 mmol, 1.0 eq.) in DMF (3 mL), the solution of 4-ethynlpiperidinium trifluoroacetate and potassium carbonate (105 mg, 0.758 mmol, 2 eq.) were added and the solution was stirred at room temperature for 2 hours. The solution was diluted with DMF (34 mL) and piperazine (2.29 g, 26.5 mmol, 70 eq.) was added. The solution was stirred at 90 °C overnight then cooled to room temperature. The solution was filtered, and the solvent was removed under reduced pressure. The obtained residue was dissolved in ethyl acetate (30 mL) and washed with water (2 x 30 mL) and 5% lithium chloride solution (30 mL). The organic phase was dried with magnesium sulphate and the solvent was removed under reduced pressure. The residues obtained were purified by silica flash chromatography (DCM/ methanol (10:1)). **8a** was obtained as a white foam (168 mg, 0.324 mmol, 86%).

^{1}H NMR (700 MHz, CDCl$_3$): δ_H = 9.98 (s, 1H, H_1), 7.74 and 7.73 (rotamers, s, 1H, H_7), 7.72 (br, 1H, H_3), 7.50 and 7.47 (rotamers, d, J = 7.8 Hz, 1H, H_5), 7.44 (t, J = 7.4 Hz, 1H, H_4), 4.83 (m, 2H, H_8), 4.13 (br s, 2H, H_{13}), 3.97 (br, 4H, H_{19}), 3.58 – 3.31 (br, 2H, H_9), 3.40 – 3.30 (br, 2H, H_{13}), 3.12 and 3.03 (rotamers, br, 4H, H_{20}), 2.66 and 2.60 (rotamers, br, 1H, H_{15}), 2.11 and 2.08 (rotamers, s, 1H, H_{17}), 1.86 – 1.67 (br, 3H, $H_{10,14}$), 1.66 – 1.42 (br, 3H, H_{14} and NH), 1.37 – 1.17 (br, 8H, CH_2 protons of alkyl chain), 0.88 – 0.80 (br, 6H, methyl protons of alkyl chain).
^{13}C NMR (176 MHz, CDCl$_3$): δ_C = 192.5 (C_1), [166.3, 166.3 (rotamers, C_{11})], [165.4, 165.3, 165.1, 165.0 ($C_{12/18}$)], 140.9 (C_6), 136.7 (C_2), [133.7, 133.4 (rotamers, C_5)], [129.2, 129.2 (rotamers, C_4)], [128.9, 128.6 (rotamers, C_7)], 128.1 (C_3), [86.9, 86.8 (rotamers, C_{16})], 69.5 (C_{17}), [50.4, 50.3 (rotamers, C_9)], [50.3, 50.2 (rotamers, C_8)], [44.0, 43.8 (rotamers, C_{20})], [41.8, 41.7 (rotamers, C_{13})], [41.2, 41.2 (rotamers, C_{19})], 38.1 (C_{10}), [31.4, 31.3 (rotamers, C_{14})], [30.8, 29.0 (CH_2 carbons of alkyl chain)], [27.3, 27.2 (rotamers, C_{15})], [24.0, 23.3 (CH_2 carbons of alkyl chain)], [14.3, 10.9, 10.9 (methyl carbons of alkyl chain)].

HRMS (ES+): Calculated for $C_{30}H_{44}N_7O^+$, 518.3607; found 518.3622.

FT-IR (ATR): ν_{max} 3307, 2954, 2926, 2855, 1699, 1535, 1487, 1442, 1373, 1313, 1242, 1193, 1136, 1027, 995, 807, 640.

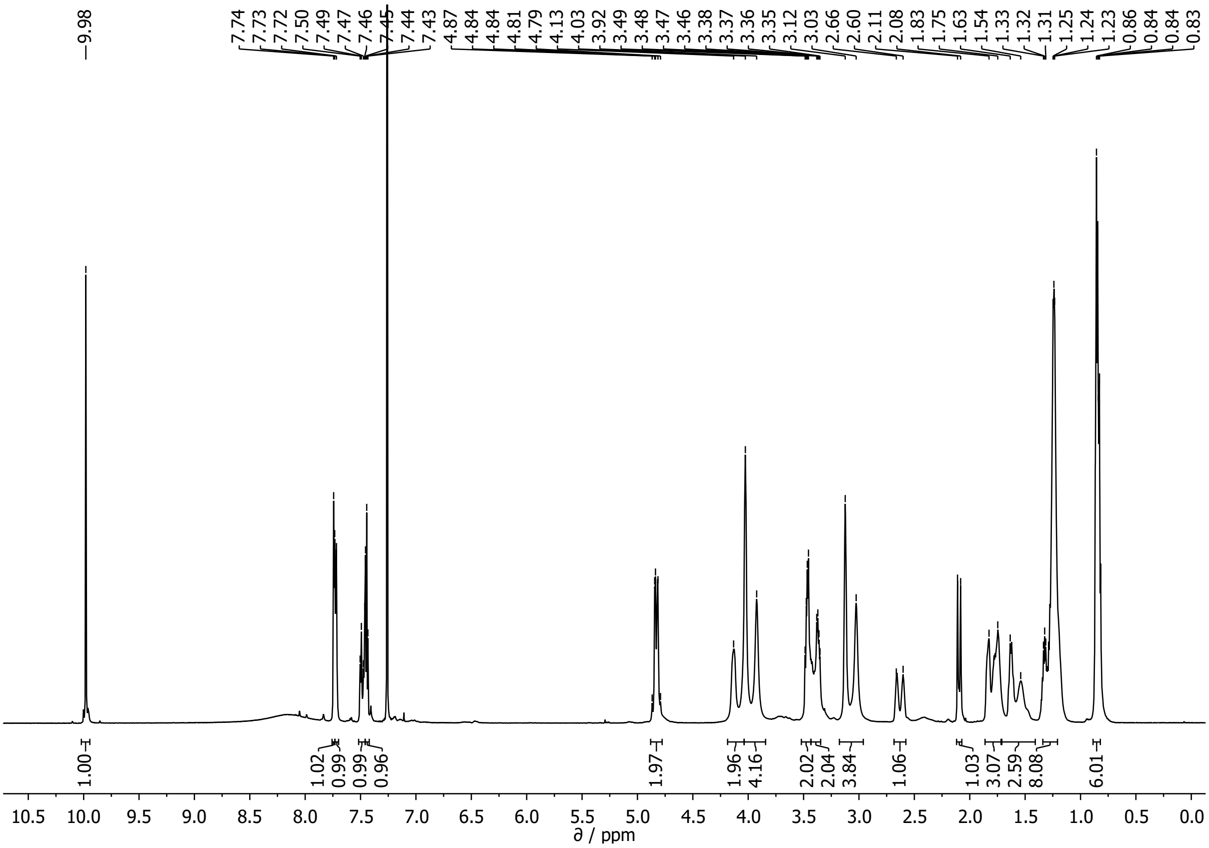

Figure S14 ^{1}H NMR spectrum (700 MHz, $CDCl_3$, 298 K) of **8a**.

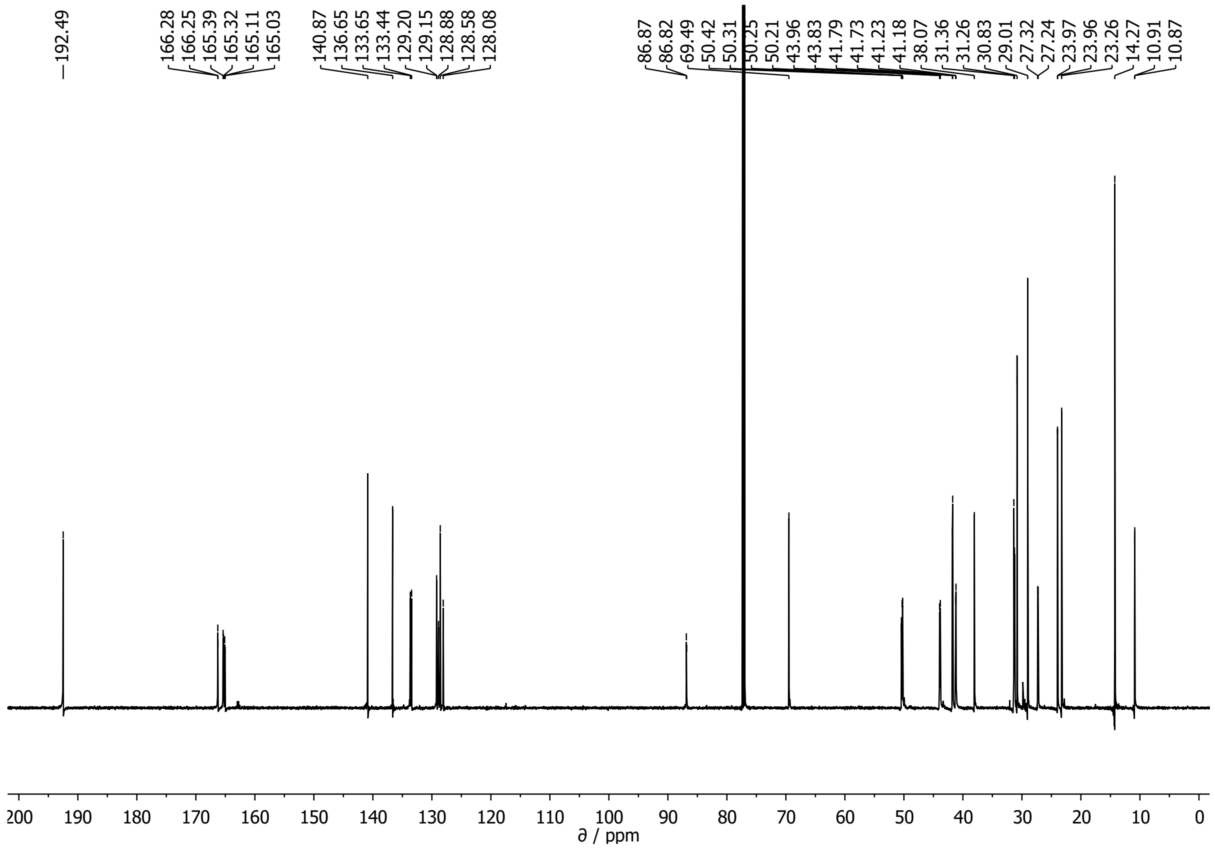

Figure S15 ^{13}C NMR spectrum (176 MHz, $CDCl_3$, 298 K) of **8a**.

Synthesis of 8b

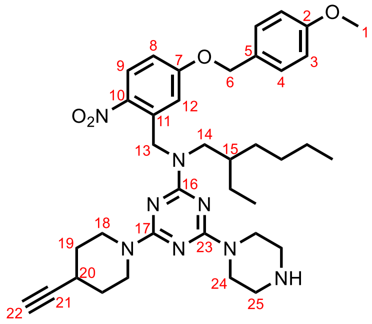

To a solution of *tert*-butyl-4-ethynylpiperidine-1-carboxylate (763 mg, 3.65 mmol, 1 eq.) in DCM (30 mL), trifluoroacetic acid (8 mL) was added and the solution was stirred for 2 hours. The solvent was removed under a nitrogen flow and the salt was redissolved in DMF (5 mL). To a solution of **6** (2.00 g, 3.65 mmol, 1 eq.) in DMF (25 mL), the solution of 4-ethynlpiperidinium trifluoroacetate and potassium carbonate (2.52 g, 18.2 mmol, 2 eq.) were added and the solution was stirred at room temperature for 2 hours. The solution was diluted with DMF (330 mL) and piperazine (12.6 g, 146 mmol, 40 eq.) was added. The solution was stirred at 90 °C overnight then cooled to room temperature. The solution was filtered, and the solvent was removed under reduced pressure. The obtained residue was dissolved in ethyl acetate (200 mL) and washed with water (2 x 200 mL) and 5% lithium chloride solution (200 mL). The organic phase was dried with magnesium sulphate and the solvent was removed under reduced pressure. The residues obtained were purified by silica flash chromatography (DCM/ methanol (20:1)). **8b** was obtained as a white foam (1.62 g, 2.42 mmol, 66%).

^{1}H NMR (700 MHz, $CDCl_3$): δ_H = 8.10 (d, J = 9.0 Hz, 1H, H_9), 7.28 (d, J = 8.1 Hz, 2H, H_4), 6.88 (d, J = 8.2 Hz, 2H, H_3), 6.86 (d, J = 9.1 Hz, 1H, H_8), 6.75 and 6.74 (s, 1H, rotamers, H_{12}), 5.07 (m, 2H, H_{13}), 4.98 (s, 2H, H_6), 4.18 (br, 1H, H_{18}), 3.92 (br, 1H, H_{18}), 3.82 (s, 1H, H_{18}), 3.80 (s, 3H, H_1), 3.60 (br, 2H, H_{24}), 3.50 – 3.44 (br, 1H, H_{18}), 3.42 (br, 2H, H_{14}), 3.23 (br, 2H, H_{24}), 2.93 (s, 2H, H_{25}), 2.79 (br, 2H, H_{25}), 2.66 and 2.54 (rotamers, 1H, H_{20}), 2.11 and 2.06 (s, 1H, rotamers, H_{22}), 1.84 (br, 1H, H_{19}), 1.78 (br, 1H, H_{15}), 1.69 (br s, 1H, NH), 1.68 – 1.42 (br, 3H, H_{19}), 1.40 – 1.18 (br, 8H, CH_2 of alkyl chain), 1.14 – 0.64 (br, 6H, methyl groups of alkyl chain).

^{13}C NMR (176 MHz, $CDCl_3$): δ_C = 166.2 (C_{16}), [165.4, 165.3, 165.2, 165.1 ($C_{17/23}$)], 162.9 (C_7), 159.9 (C_2), 141.4 (C_{10}), 139.5 and 139.4 (rotamers, C_{11}), 129.6 (C_4), 127.8 (C_9), 127.7 (C_5), 114.4 (C_{12}), 114.3 (C_3), 112.7 and 112.6 (C_8), 87.1 and 87.0 (rotamers, C_{21}), 70.4 (C_6), 69.4 and 69.3 (rotamers, C_{22}), 55.4 (C_1), 51.1 (C_{14}), 49.0 (C_{13}), 45.8 and 45.5 (rotamers, C_{25}),

43.9 and 43.6 (rotamers, C_{24}), 41.8 and 41.7 (rotamers, C_{18}), 38.3 (C_{15}), 31.4 and 31.2 (rotamers, C_{19}), 30.8, 29.0 (carbons of CH_2), 27.4 and 27.3 (rotamers, C_{20}), 24.0, 23.3 (carbons of CH_2 chain), 14.3, 10.9 (methyl carbons of chain).

HRMS (ES+): Calculated for $C_{37}H_{51}N_8O_4^+$, 671.4028; found 671.4008.

FT-IR (ATR): ν_{max} 3289, 2954, 2928, 2885, 1612, 1579, 1535, 1516, 1485, 1442, 1372, 1350, 1315, 1284, 1245, 1193, 1175, 1072, 1035, 1001, 808.

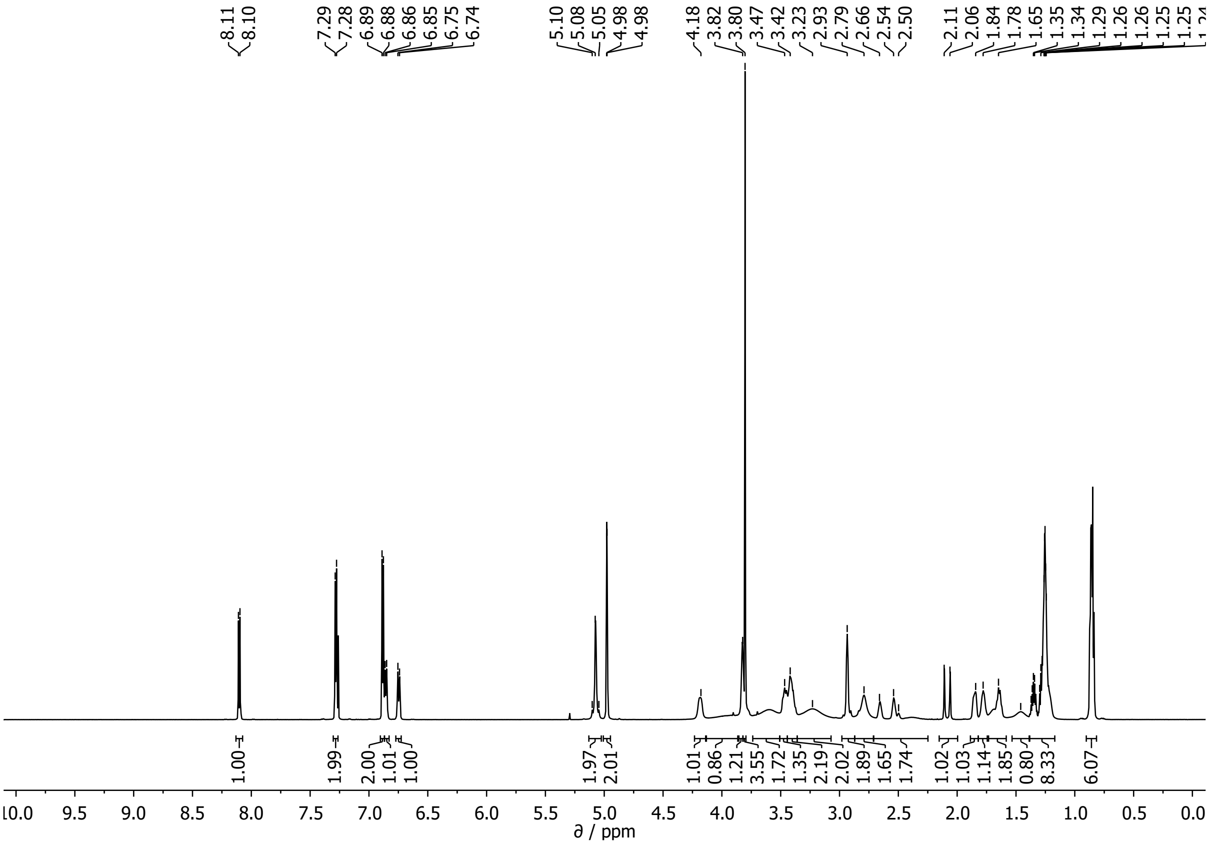

Figure S16 ^{1}H NMR spectrum (700 MHz, $CDCl_3$, 298 K) of **8b**.

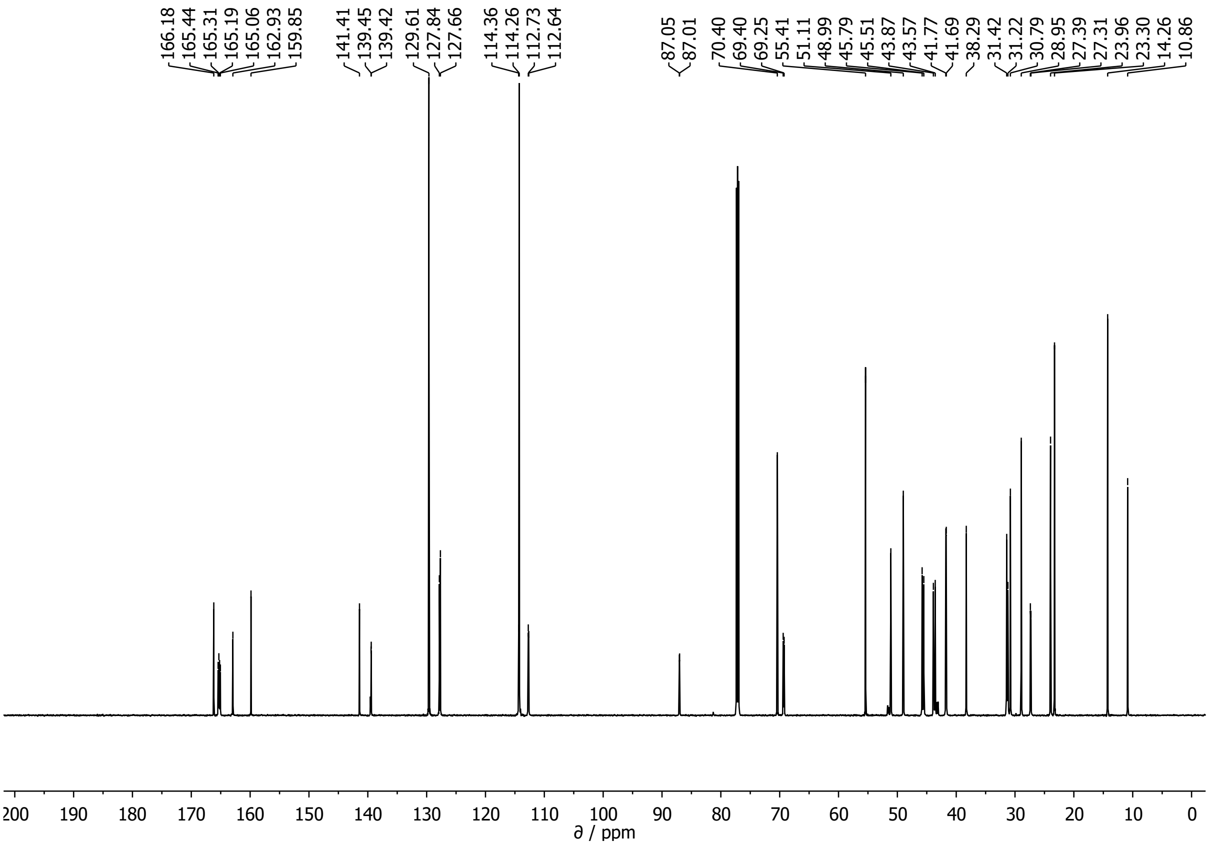

Figure S17 ^{13}C NMR spectrum (176 MHz, $CDCl_3$, 298 K) of **8b**.

Synthesis of 9a

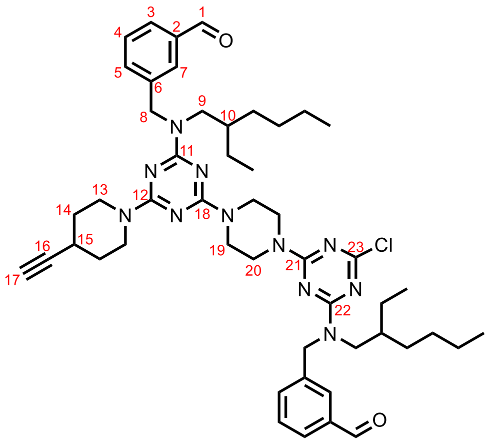

To a solution of **5** (128 mg, 0.324 mmol, 1.0 eq.) in THF (2 mL), a solution of **8a** (168 mg, 0.324 mmol, 1.0 eq.) in THF (1 mL) and DIPEA (113 µL, 0.648 mmol, 2 eq.) were added and the solution was stirred at room temperature for 2 hours. The solution was diluted in ethyl acetate (20 mL). The organic phase was washed with water (2 x 20 mL), dried with magnesium sulphate and the solvent was removed under reduced pressure. The residues obtained were purified by silica flash chromatography (pet ether/ ethyl acetate (9:1)). **9a** was obtained as a colourless oil (240 g, 0.274 mmol, 85%).

^{1}H NMR (700 MHz, $CDCl_3$): δ_H = [10.00, 9.98, 9.97, 9.96 (s, 2H, H_1)], 7.79 – 7.70 (br, 4H, $H_{3,7}$), 7.54 – 7.46 (br, 3H, $H_{4,5}$), 7.44 (t, J = 7.4 Hz, 1H, H_4), 4.96 – 4.77 (br, 4H, H_8), 4.17 and 4.07 (rotamers, br, 2H, H_{13}), 3.89 – 3.55 (br, 8H, $H_{19,20}$), 3.55 – 3.32 (br, 6H, $H_{9,13}$), 2.65 and 2.60 (rotamers, br, 1H, H_{15}), 2.11 and 2.08 (rotamers, s, 1H, H_{17}), 1.85 – 1.69 (br, 4H, $H_{10,14}$), 1.63 and 1.54 (rotamers, br, 2H, H_{14}), 1.37 – 1.14 (br, 16H, CH_2 protons of alkyl chain), 0.89 – 0.80 (br, 12H, methyl protons of alkyl chain).
^{13}C NMR (176 MHz, $CDCl_3$): δ_C = [192.5, 192.3, 192.2 (C_1)], [169.7, 169.6 (rotamers, C_{23})], [166.3, 165.9, 165.9, 165.8 ($C_{11,22}$)], [165.6, 165.5, 165.4, 165.2, 165.2, 165.1, 165.1, 164.6, 164.6, 164.6, 164.5 ($C_{12,18,21}$)], [141.1, 139.4, 139.2 (C_6)], [136.8, 136.6 (C_2)], [134.0, 133.7, 133.6, 133.4 (C_5)], 129.4 (C_4), 129.2 (C_7), 129.1 (C_4), [128.7, 128.6, 128.6, 128.5, 128.5, 128.2 ($C_{3,7}$)], [87.0, 86.9 (rotamers, C_{16})], 69.4 (C_{17}), [50.2, 50.2 (C_9)], [50.2, 50.0, 49.9 (C_8)], [43.5, 43.4, 43.2, 43.1, 43.1, 42.9, 42.8, 42.7, 42.7 ($C_{19,20}$)], [41.8, 41.7 (rotamers, C_{13})], [38.1, 37.8, 37.5 (C_{10})], [31.4, 31.3 (rotamers, C_{14})], [30.8, 30.8, 30.8, 30.5, 29.8, 29.0, 28.6 (CH_2 carbons of alkyl chains)], [27.4, 27.3 (rotamers, C_{15})], [24.0, 23.8, 23.3, 23.2 (CH_2 carbons of alkyl chain)], [14.3, 14.2, 10.9, 10.8, 10.8 (methyl carbons of alkyl chain)].
HRMS (ES+): Calculated for $C_{49}H_{67}ClN_{11}O_2^+$, 876.5162; found 876.5166.

FT-IR (ATR): ν_{max} 3300, 2956, 2926, 2856, 1702, 1565, 1536, 1489, 1436, 1369, 1312, 1283, 1232, 1165, 994, 802.

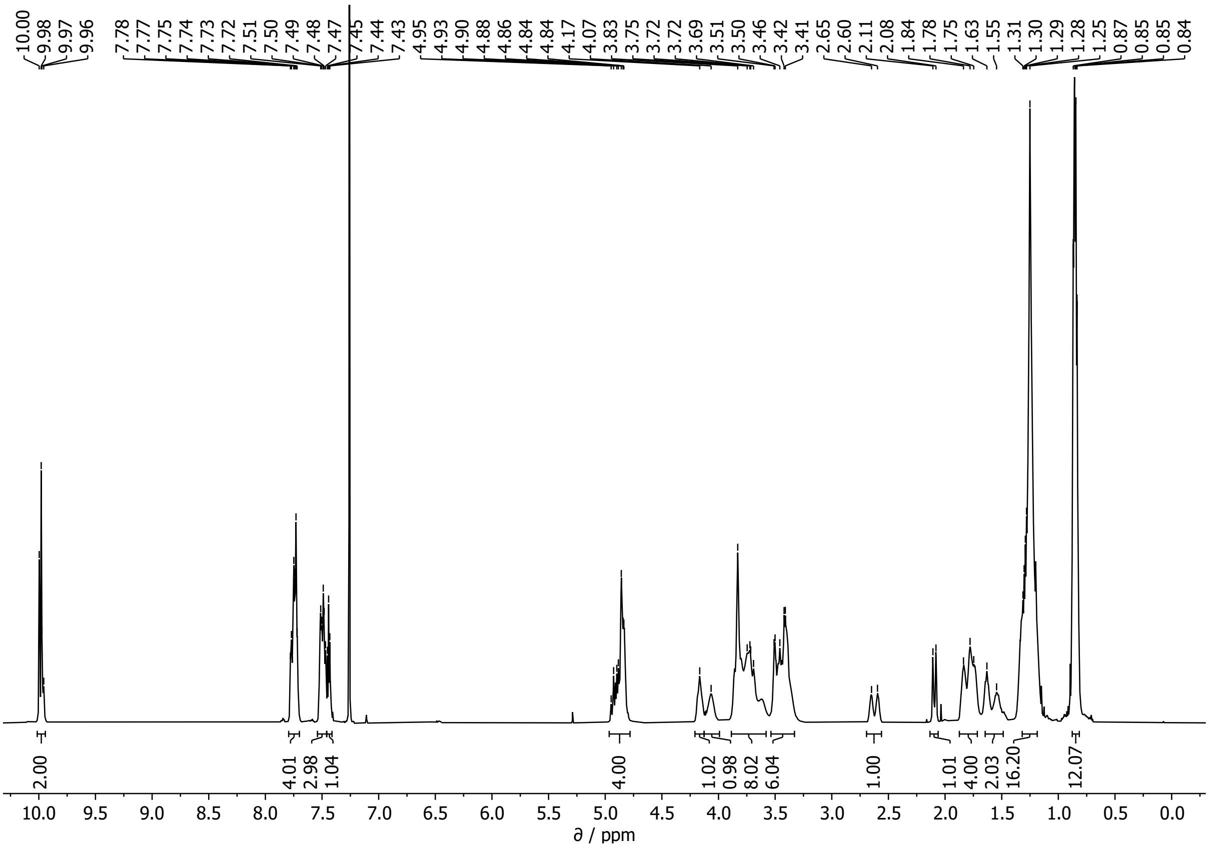

Figure S18 ^{1}H NMR spectrum (700 MHz, $CDCl_3$, 298 K) of **9a**.

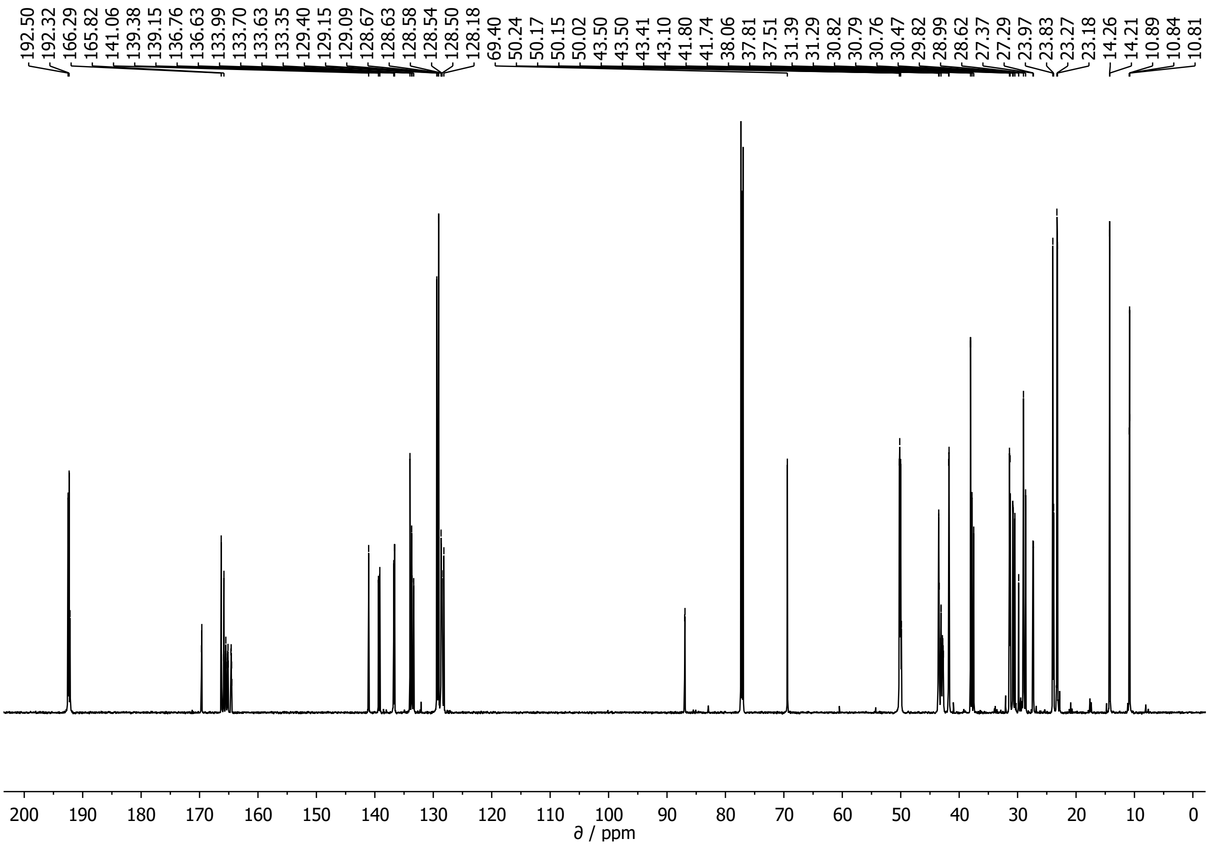

Figure S19 ^{13}C NMR spectrum (176 MHz, $CDCl_3$, 298 K) of **9a**.

Synthesis of 9b

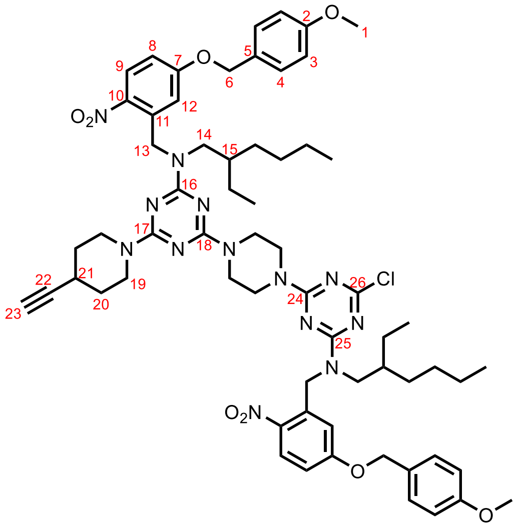

To a solution of **6** (548 mg, 1.00 mmol, 1.0 eq.) in THF (8 mL), a solution of **8b** (671 mg, 1.00 mmol, 1.0 eq.) in THF (2 mL) and DIPEA (700 µL, 4 mmol, 4 eq.) were added and the solution was stirred at room temperature for 2 hours. The solvent was removed under reduced pressure and the obtained residue was dissolved in ethyl acetate (30 mL). The organic phase was washed with water (2 x 30 mL), dried with magnesium sulphate and the solvent was removed under reduced pressure. The residues obtained were purified by silica flash chromatography (pet ether/ ethyl acetate (85:15)). **9b** was obtained as a white foam (1.02 g, 0.861 mmol, 86%).

^{1}H NMR (700 MHz, CDCl$_3$): δ_H = [8.20, 8.19, 8.17, 8.16 (rotamers, 1H, H_9)], [8.13, 8.11, 8.10 (rotamers, 1H, H_9)], [7.29, 7.28, 7.27, 7.26, 7.26, 7.24, 7.23, 7.22 (4H, H_4)], 6.94 - 6.85 (broad, 6H, $H_{2,8}$), [6.77, 6.77, 6.75, 6.74, 6.74, 6.72, 6.72 (rotamers, 1H, H_{12})], [6.66, 6.66, 6.65, 6.65, 6.63, 6.63, 6.62, 6.61 (rotamers, 1H, H_{12})], 5.25 – 5.04 (broad, 4H, H_{13}), 5.02 - 4.95 (broad, 4H, H_6), 4.22 – 3.09 (broad, 22H, $H_{1,14,19}$ and piperazine protons), 2.67 and 2.55 (broad, 1H, H_{21}), [2.13, 2.12, 2.12, 2.11, 2.08, 2.07, 2.07, 2.06 (rotamers, 1H, H_{23})], 1.87 - 1.64 (broad, 6H, $H_{15,20}$), 1.38 - 1.20 (broad, 16H, CH_2 of alkyl chain), 0.90 - 0.84 (broad, 12H, methyl groups of alkyl chain).

^{13}C NMR (176 MHz, CDCl$_3$): δ_C = [169.7, 169.7, 169.6 169.6 (rotamers, C_{26})], [166.2, 166.2, 166.0, 165.8 ($C_{16,25}$)], [165.5, 165.4, 165.4, 165.3, 165.2, 165.1, 165.0, 165.0, 164.6, 164.5, 164.4, 164.4 ($C_{17,18,24}$)], [163.1, 163.1, 162.9, 162.9 (C_7)], [160.0, 159.9, 159.9, 159.9, 159.8, 159.8 (C_2)], [141.5, 141.4, 141.4, 141.4, 141.2 (C_{10})], [139.3, 139.3, 139.2, 137.4, 137.3, 137.2 (C_{11})], [129.6, 129.6, 129.5, 129.5, 129.4, 129.1 (C_4)], [128.5, 128.5, 128.3, 128.0, 127.9, 127.8 (C_9)], [127.6, 127.4, 127.4 (C_5)] , [114.6, 114.5 (C_{12})], [114.4, 114.4, 114.3, 114.3, 114.3 (C_3)],

[113.5, 113.5 (C_{12})], [112.8, 112.7, 112.6, 112.5 (C_8)], [87.0, 87.0, 86.9 (rotamers, C_{22})], [70.6, 70.5, 70.5, 70.5, 70.4, 70.4, 70.4 (C_6)], [69.5, 69.4, 69.3, 69.3 (rotamers, C_{23})], [55.4, 55.4, 55.4 (C_1)], [51.2, 51.2, 51.1, 51.1, 51.1 (C_{14})], [49.1, 49.0, 49.0, 48.9, 48.9, 48.8 (C_{13})], [43.6, 43.6, 43.4, 43.4, 43.3, 43.2, 43.2, 43.0, 43.0, 42.9, 42.8, 42.7, 42.7, 43.5 (carbons of piperazine rings)], [41.8 and 41.7 (rotamers, C_{19})], [38.0, 38.0, 37.7 (C_{15})], [31.4 and 31.2 (rotamers, C_{20})], [30.8, 30.8, 30.7, 30.7, 30.7, 30.4, 28.9, 28.9, 28.5 (CH_2 carbons of alkyl chain)], [27.4 and 27.3 (rotamers, C_{21})], [24.0, 23.9, 23.8, 23.3, 23.2 (CH_2 carbons of alkyl chain)], [14.3, 14.2, 14.2, 10.9, 10.9, 10.8, 10.7 (methyl carbons of alkyl chain)].

HRMS (ES+): Calculated for $C_{63}H_{81}ClN_{13}O_8^+$, 1182.6020; found 1182.5980.

FT-IR (ATR): ν_{max} 3298, 2955, 2927, 2857, 1612, 1564, 1534, 1514, 1485, 1434, 1370, 1351, 1316, 1280, 1230, 1173, 1112, 1072, 1034, 993, 968, 946, 909, 843, 823, 806, 753, 732, 649, 514, 443.

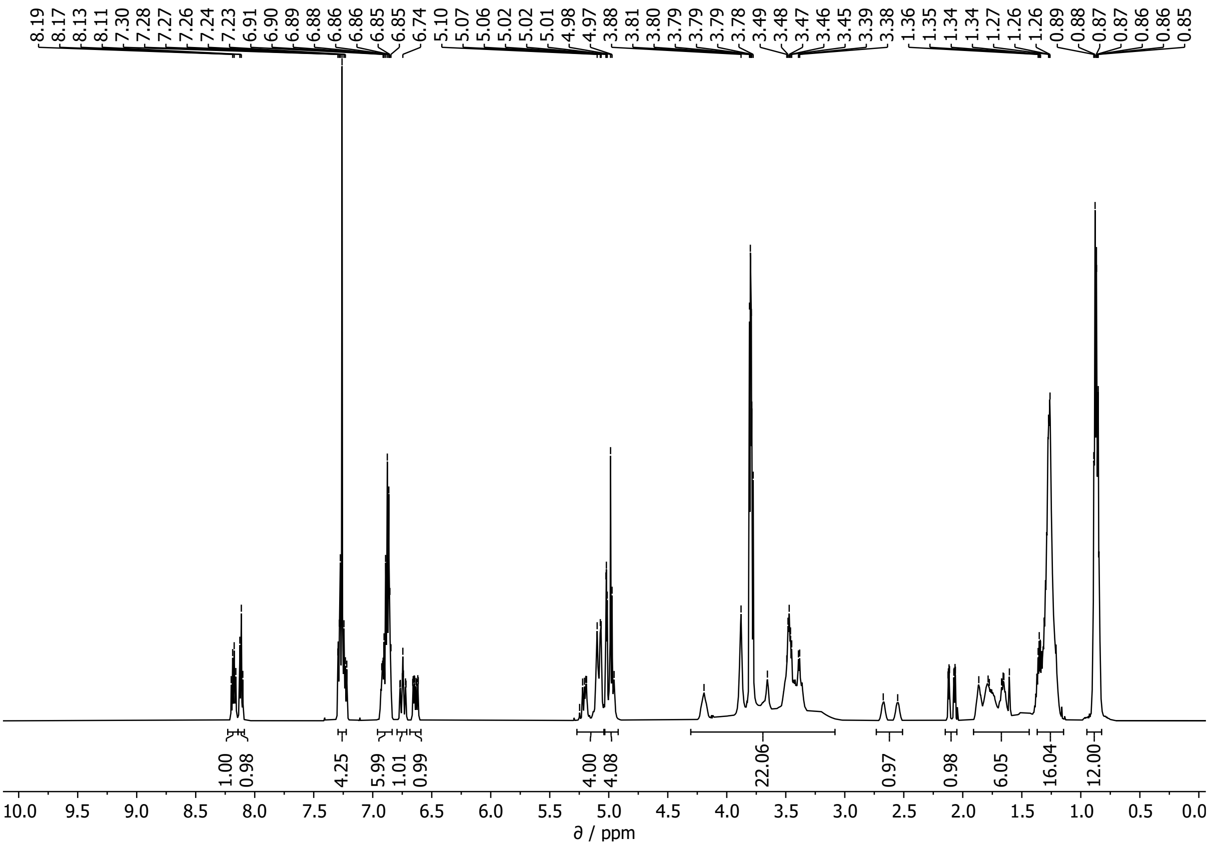

Figure S20 ^{1}H NMR spectrum (700 MHz, $CDCl_3$, 298 K) of **9b**.

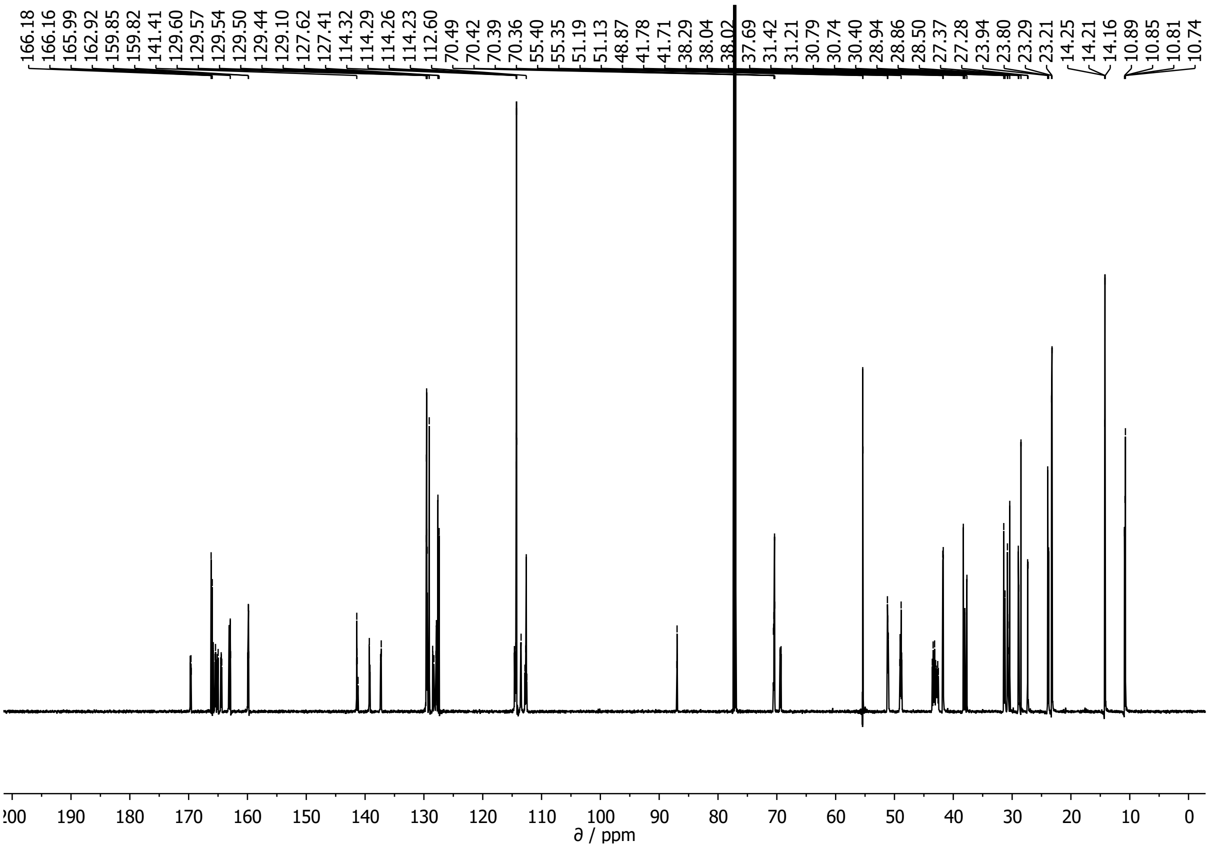

Figure S21 ^{13}C NMR spectrum (176 MHz, $CDCl_3$, 298 K) of **9b**.

Synthesis of 10a

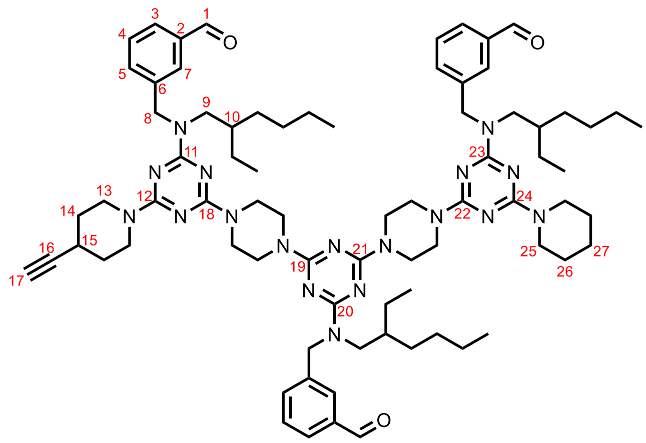

To a solution of **9a** (240 mg, 0.274 mmol, 1.0 eq.) in THF (3 mL), **7a** (676 mg, 1.37 mmol, 5.0 eq) and DIPEA (239 µL, 1.37 mmol, 5.0 eq.) were added. The solution was heated at 65 °C under microwave irradiation for 30 minutes then cooled to room temperature. The solvent was removed under reduced pressure and the obtained residue was redissolved in ethyl acetate (30 mL). The organic layer was washed with water (30 mL), dried with magnesium sulphate and the solvent removed under reduced pressure. The obtained residue was purified by silica flash chromatography (pet ether/ ethyl acetate (8:2) then ethyl acetate/ methanol (80:20)). **10a** was obtained as a white foam (225 mg, 0.169 mmol, 62%).

^{1}H NMR (700 MHz, $CDCl_3$): δ_H = [9.98, 9.97 (s, 3H, H_1)], 7.79 – 7.68 (br, 6H, $H_{3,7}$), 7.52 (br, 3H, H_5), 7.44 (br, 3H, H_4), 4.86 (br, 6H, H_8), 4.18 and 4.08 (rotamers, br, 2H, H_{13}), 3.92 – 3.59 (br, 20H, H_{25} and protons of piperazine rings), 3.45 (br, 7H, $H_{9,13}$), 3.36 (br, 1H, H_{13}), 2.65 and 2.59 (rotamers, br, 1H, H_{15}), 2.11 and 2.08 (rotamers, s, 1H, H_{17}), 1.84 (br, 1H, H_{14}), 1.78 (br, 4H, $H_{10,14}$), 1.64 (br, 2H, $H_{14,27}$), 1.59 (br, 1H, H_{27}), 1.55 (br, 3H, $H_{14,26}$), 1.47 (br, 2H, H_{26}), 1.37 – 1.16 (br, 24H, CH_2 protons of alkyl chains), 0.90 – 0.80 (br, 18H, methyl protons of alkyl chains).

^{13}C NMR (176 MHz, $CDCl_3$): δ_C = [192.6, 192.6, 192.5 C_1)], 166.4 ($C_{11,20,23}$), [165.6, 165.6, 165.5, 165.4, 165.3, 165.2, 165.1 ($C_{12,18,19,21,22,24}$)], [141.3, 141.3, 141.2, 141.1 (C_6)], 136.6 (C_2), [133.9, 133.8 (C_5)], [129.1, 129.1, 129.1 (C_4)], [128.8, 128.7 (C_7)], [128.5, 128.4, 128.2 (C_3)], [87.1, 87.0 (rotamers, C_{16})], 69.4 (C_{17}), 50.0 ($C_{8,9}$), [44.3, 44.2 (rotamers, C_{25})], 43.2 (carbons of piperazine rings)], [41.8, 41.8 (rotamers, C_{13})], 38.1 (C_{10}), [31.4, 31.3 (rotamers, C_{14})], [30.8, 29.8, 29.1, 29.0 (CH_2 carbons of alkyl chains)], [27.4, 27.3 (rotamers, C_{15})], [26.0, 25.9 (rotamers, C_{26})], [25.2, 25.1 (rotamers, C_{27})], [24.0, 23.3 (CH_2 carbons of alkyl chains)], [14.3, 10.9 (methyl carbons of alkyl chains)].

HRMS (ES+): Calculated for $C_{77}H_{109}N_{18}O_3^+$, 1333.8925; found 1333.8935.

FT-IR (ATR): ν_{max} 3309, 2955, 2925, 2854, 2728, 1702, 1527, 1480, 1432, 1368, 1352, 1310, 1240, 1183, 997, 807, 778, 735, 651.

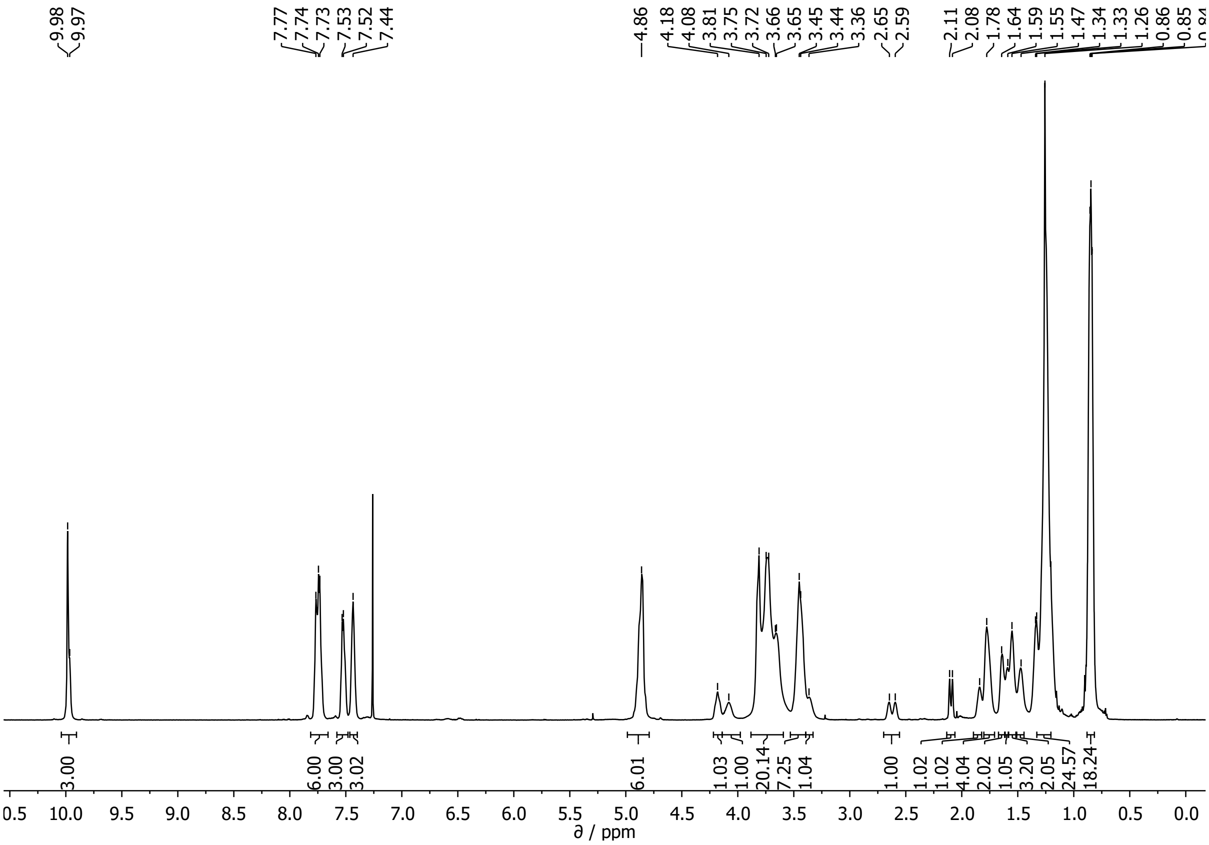

Figure S22 ^{1}H NMR spectrum (700 MHz, $CDCl_3$, 298 K) of **10a**.

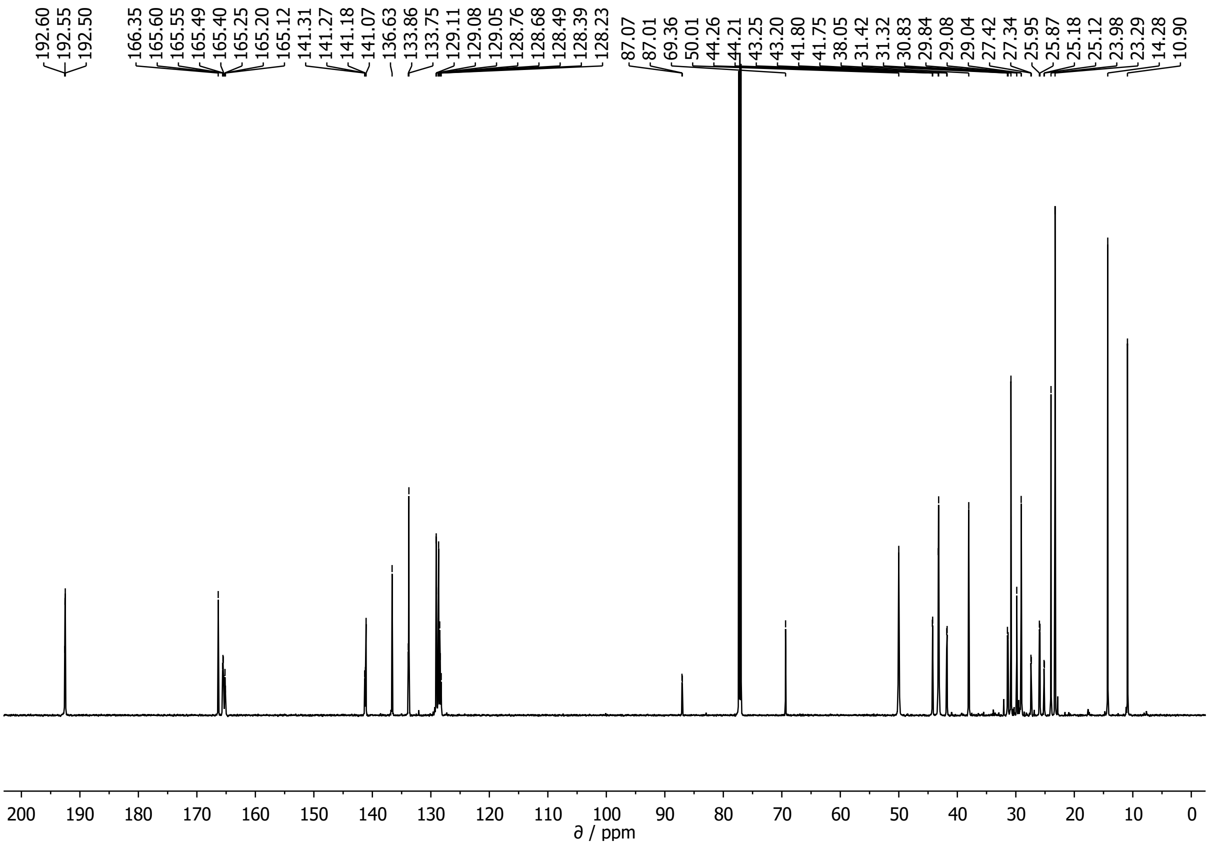

Figure S23 ^{13}C NMR spectrum (176 MHz, $CDCl_3$, 298 K) of **10a**.

Synthesis of 10b

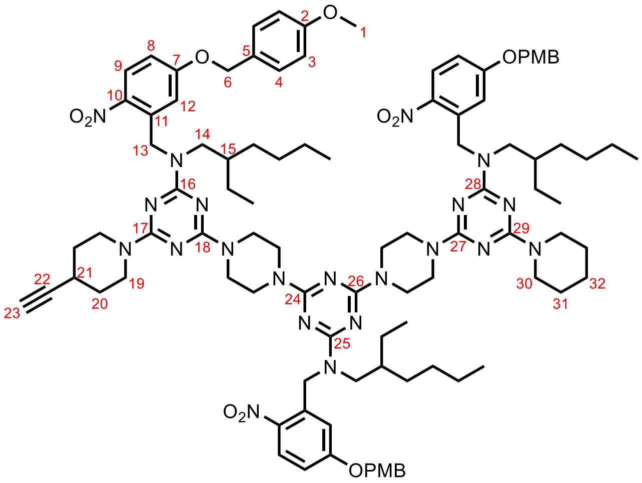

To a solution of **9b** (1.02 g, 0.861 mmol, 1.0 eq.) in THF (9 mL), **7b** (2.78 g, 2.78 mmol, 5.0 eq.) and DIPEA (749 µL, 4.30 mmol, 5.0 eq.) were added. The solution was heated at 65 °C under microwave irradiation for 30 minutes then cooled to room temperature. The solvent was removed under reduced pressure and the obtained residue was redissolved in ethyl acetate (30 mL). The organic layer was washed with water (30 mL), dried with magnesium sulphate and the solvent removed under reduced pressure. The obtained residue was purified by silica flash chromatography (pet ether/ ethyl acetate (8:2) then ethyl acetate/ methanol (85:15)). **10b** was obtained as a white foam (1.41 g, 0.786 mmol, 91%).

^{1}H NMR (700 MHz, CDCl$_3$): δ_H = 8.23 – 8.03 (br, 3H, H_9), 7.43 – 7.17 (br, 6H, H_4), 6.90 – 6.82 (br, 9H, $H_{3,8}$), 6.83 – 6.71 (br, 3H, H_{12}), 5.28 – 5.05 (br, 6H, H_{13}), 5.03 – 4.89 (br, 6H, H_6), 4.23 – 3.02 (br, 39H, $H_{1,14,19,30}$ and protons of piperazine rings), 2.68 and 2.56 (rotamers, br, 1H, H_{21}), [2.14, 2,13, 2.09, 2.08 (rotamers, s, 1H, H_{23})], 1.88 (br, 1H, H_{20}), 1.82 (br, 3H, H_{15}), 1.67 (br, 3H, H_{20}), 1.59 (br, 4H, H_{31}), 1.45 (br, 2H, H_{32}), 1.42 – 1.20 (br, 24H, CH_2 protons of alkyl chain), 0.93 – 0.81 (br, 18H, methyl protons of alkyl chain).

^{13}C NMR (176 MHz, CDCl$_3$): δ_C = 166.2 ($C_{16,25,28}$), [165.5, 165.4, 165.4, 165.3, 165.3, 165.2, 165.2, 165.1, 165.1, 165.0, 165.0, 164.9 ($C_{17,18,24,26,27,29}$)], [162.9, 162.9 (C_7)], 159.8 (C_2), 141.4 (C_{10}), [139.6, 139.4, 139.3 (C_{11})], 129.5 (C_4), [127.9, 127.8 (C_9)], [127.7, 127.6, 127.6 (C_5)], 114.4 (C_{12}), 114.2 (C_3), [112.8, 112.7, 112.6 (C_8)], 87.0 (C_{22}), 70.3 (C_6), 69.4 and 69.3 (rotamers, C_{23}), 55.3 (C_1), [51.0, 51.0 (C_{14})], [48.9, 48.9, 48.8 (C_{13})], [44.2, 44.1, 43.3, 43.1, 42.9, 41.7, 41.7 ($C_{19,30}$ and carbons of piperazine rings)], [38.3, 38.2 (C_{15})], 31.4 and 31.2 (rotamers, C_{20}), [30.8, 29.8, 28.9 (CH_2 carbons of alkyl chain)], 27.4 and 27.3 (rotamers, C_{21}),

26.0 and 25.7 (rotamers, C_{31}), 25.1 and 25.0 (rotamers, C_{32}), [24.0, 23.3 (CH_2 carbons of alkyl chain)], [14.2, 10.8 (methyl carbons of alkyl chain)].

HRMS (ES+): Calculated for $C_{98}H_{130}N_{21}O_{12}^{+}$, 1793.0208; found 1793.0175.

FT-IR (ATR): ν_{max} 3299, 2955, 2928, 2855, 1612, 1579, 1530, 1480, 1433, 1369, 1351, 1317, 1251, 1191, 1178, 1072, 1035, 997, 843, 808, 753, 735.

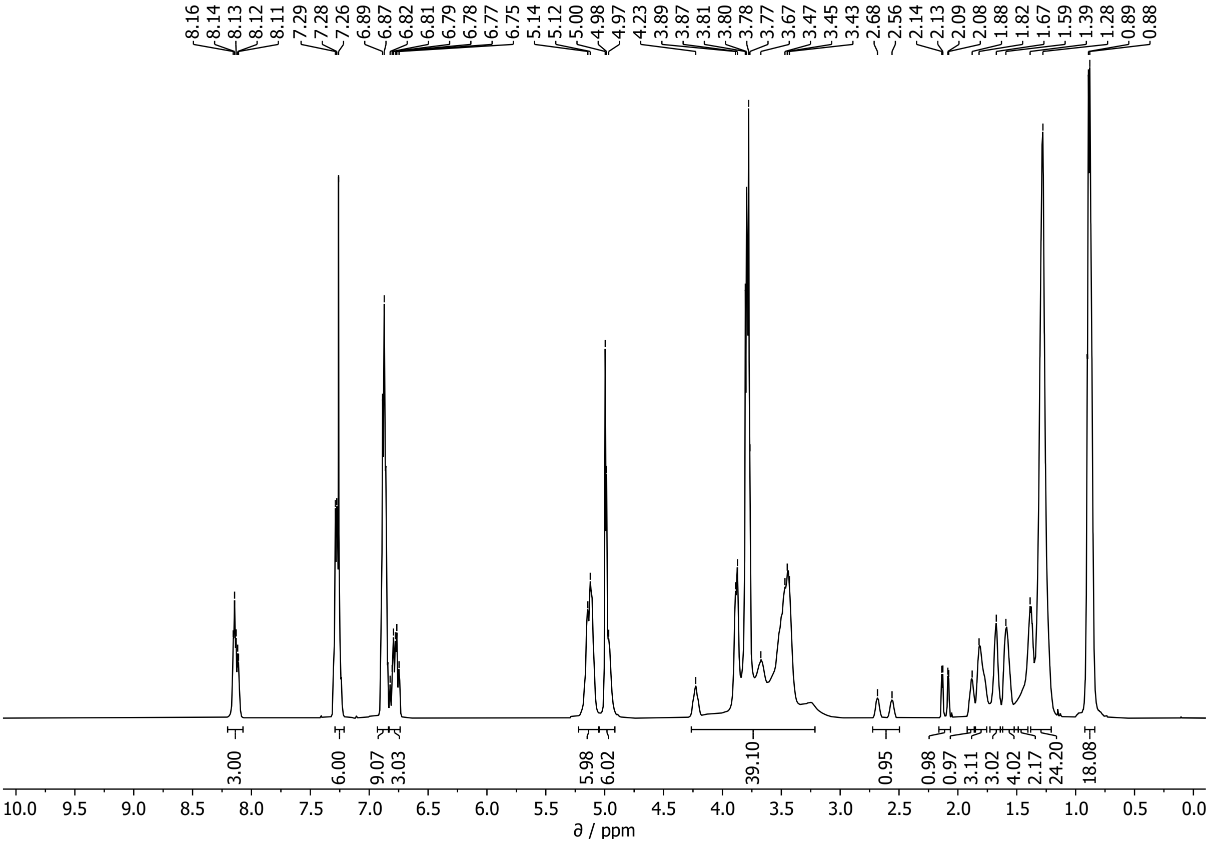

Figure S24 ^{1}H NMR spectrum (700 MHz, $CDCl_3$, 298 K) of **10b**.

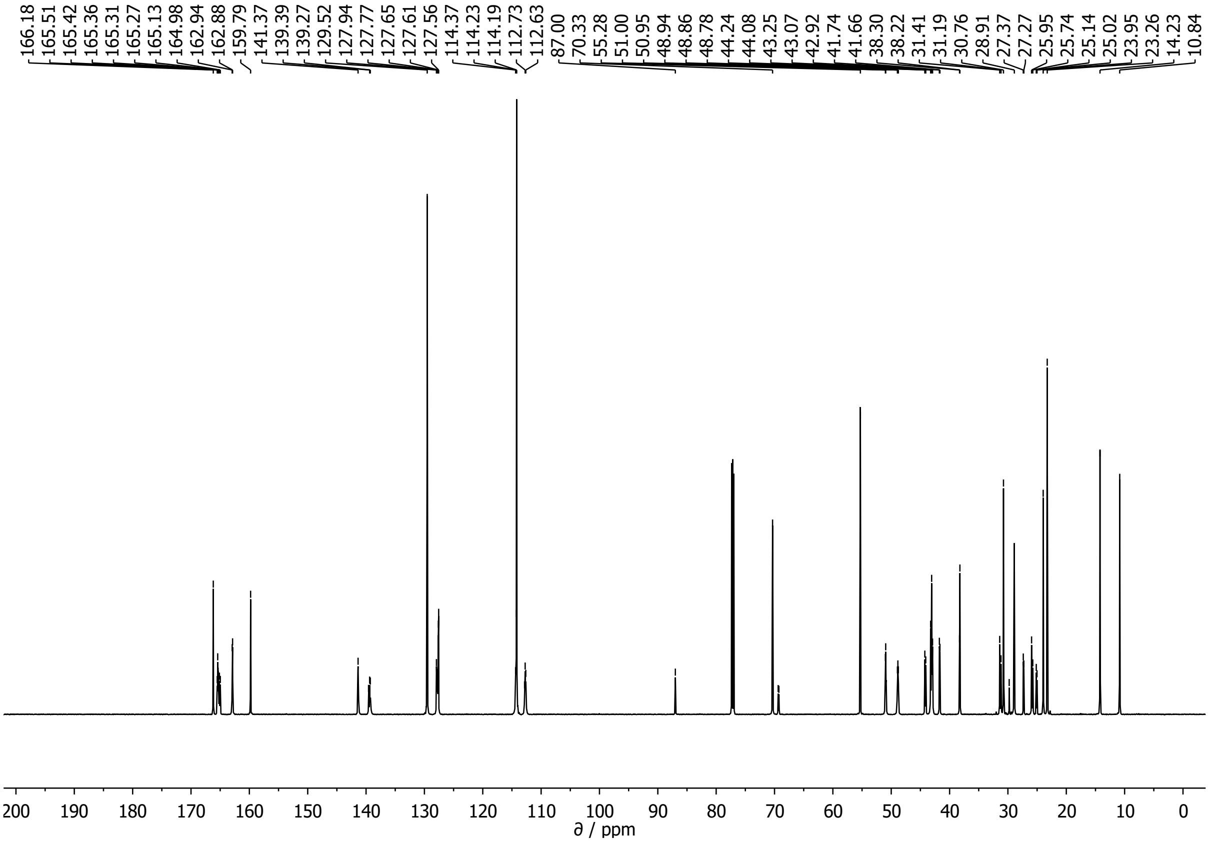

Figure S25 ^{13}C NMR spectrum (176 MHz, $CDCl_3$, 298 K) of **10b**.

Synthesis of 11

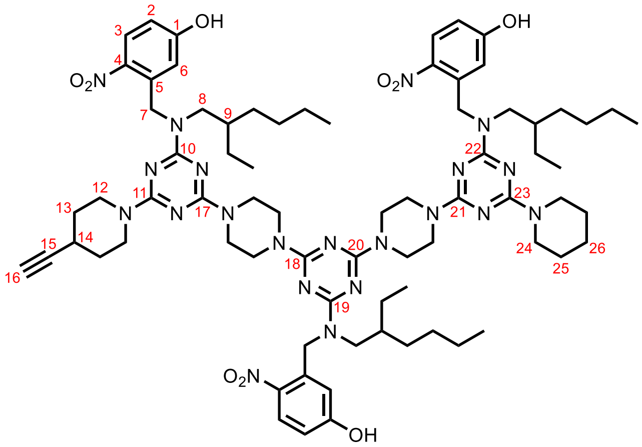

To a solution of **10b** (50 mg, 0.028 mmol, 1.0 eq.) in DCM (4 mL), trifluoroacetic acid (4 mL) was added and the solution was stirred at room temperature for 5 minutes. The solvent was removed under reduced pressure and the residue was redissolved in ethyl acetate (10 mL). The organic phase was washed with water (10 mL) and the aqueous phase was extracted with ethyl acetate (10 mL). The combined organic phases were dried over magnesium sulphate and the solvent was removed under reduced pressure. The obtained residue was purified by silica flash chromatography (DCM / methanol (95:5)). **11** was obtained as a yellow foam (40 mg, 0.028 mmol, quantitative).

^{1}H NMR (700 MHz, $CDCl_3$): δ_H = 8.04 (br, 3H, H_3), 6.71 – 6.14 (br, 6H, $H_{2,6}$), 5.07 (br, 6H, H_7), 4.20 – 3.14 (br, 30H, $H_{8,12,24}$ and protons of piperazine rings), [2.67, 2.63, 2.56, 2.53 (rotamers, br s, 1H, H_{14})], [2,12, 2.10, 2.06, 2.04 (rotamers, s, 1H, H_{16})], 1.83 (br, 4H, $H_{9,13,25}$), 1.70 – 1.34 (br, 7H, $H_{13,25,28}$), 1.26 (br, 24H, CH_2 protons of alkyl chains), 0.87 (br, 18H, methyl protons of alkyl chains).

^{13}C NMR (176 MHz, $CDCl_3$): δ_C = [166.3, 166.1, 166.0, 165.9 ($C_{10,19,22}$)], [165.5, 165.3, 165.3, 165.1, 165.0, 165.0, 164.9 ($C_{11,17,18,20,21,23}$)], [161.7, 161.2 (C_1)], [141.1, 140.9, 140.7 (C_4)], [139.8, 139.7, 139.6 (C_5)], [128.6, 128.4, 128.3 (C_3)], [114.8, 114.8, 114.7 (C_6)], [113.8, 113.5, 113.4, 113.3, 113.2 (C_2)], 86.8 (C_{15}), [69.6, 69.6, 69.5, 69.5 (rotamers, C_{16})], 51.4 (C_8), [49.1, 49.0, 48.8 (C_7)], [46.4, 44.5, 44.4, 43.2, 43.0, 41.9, 41.8 ($C_{12,24}$ and carbons of piperazine rings)], 38.3 (C_9), [31.4, 31.3 ($C_{13,25}$)], [31.2, 30.8, 30.7, 29.8, 29.8, 29.5 (CH_2 carbons of alkyl chains)], [29.0, 27.2 (rotamers, C_{14})], [26.8, 26.3, 25.9, 25.9 ($C_{13,25}$)], 25.7 (C_{26}), [25.0, 25.0, 24.9 ($C_{13,25}$)], [24.0, 23.3 (CH_2 carbons of alkyl chains)], [14.3, 14.3, 14.2, 10.9, 10.9, 10.9 (methyl carbons of alkyl chains)].

HRMS (ES+): Calculated for $C_{74}H_{106}N_{21}O_9^{+}$, 1432.8482; found 1432.8503.

FT-IR (ATR): ν_{max} 3305, 3080 (br), 2954, 2925, 2855, 1615, 1596, 1580, 1524, 1478, 1433, 1368, 1330, 1299, 1255, 1191, 1071, 996, 844, 808.

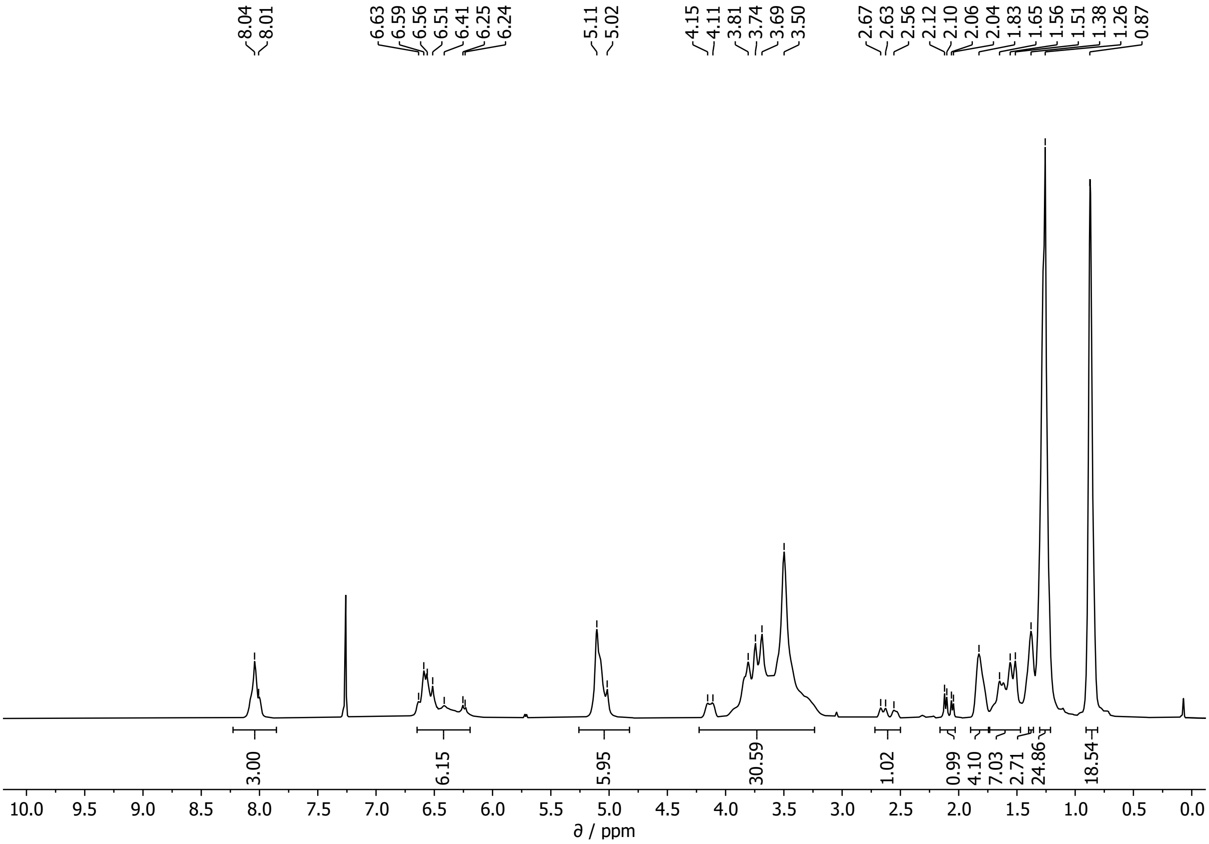

Figure S26 ^{1}H NMR spectrum (700 MHz, $CDCl_3$, 298 K) of **11**.

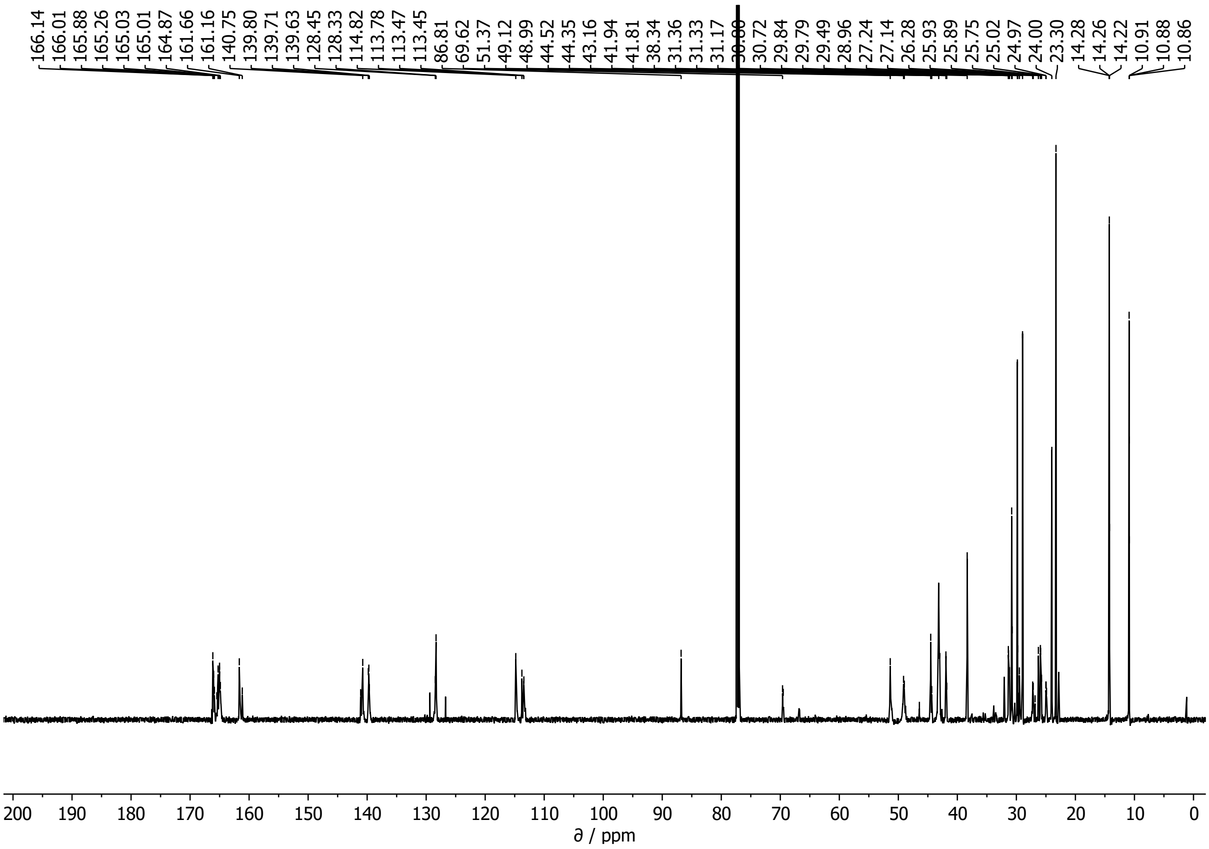

Figure S27 ^{13}C NMR spectrum (176 MHz, $CDCl_3$, 298 K) of **11**.

Synthesis of 12

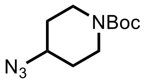

Compound **12** was synthesised according to the literature procedure.[3]

Synthesis of 13

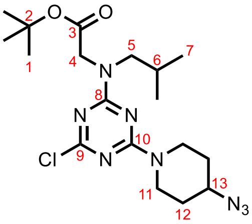

To a solution of *tert*-butyl-4-azidopiperidine-1-carboxylate (337 mg, 1.49 mmol, 1.0 eq.) in DCM (9 mL), trifluoroacetic acid (3 mL) was added and the solution was stirred for 2 hours. The solvent was removed under a nitrogen flow and the salt was redissolved in THF (2 mL). To a solution of **3** (500 mg, 1.49 mmol, 1.0 eq.) in THF (6 mL), the solution of 4-azidopiperidinium trifluoroacetate and DIPEA (1.0 mL, 6.0 mmol, 4 eq.) were added dropwise and the solution was stirred at room temperature of 2 hours. The solvent was removed under reduced pressure and the obtained residue was redissolved in ethyl acetate (20 mL). The organic layer was washed with water (20 mL), dried with magnesium sulphate and the solvent removed under reduced pressure. The obtained residue was purified by silica flash chromatography (pet ether/ ethyl acetate (95:5)). **13** was obtained as a colourless oil (561 mg, 1.32 mmol, 89%).

^{1}H NMR (700 MHz, CDCl$_3$): δ_H = 4.20 and 4.15 (rotamers, br, 2H, H_{11}), 4.08 (rotamer, m, 1H, H_4), 3.98 (rotamer, s, 1H, H_4), 3.66 (m, 1H, H_{13}), 3.45 – 3.32 (br, 4H, $H_{5,11}$), 1.97 (non, J = 6.8 Hz, 1H, H_6), 1.89 and 1.58 (rotamers, br, 4H, H_{12}), 1.44 and 1.40 (rotamers, s, 9H, H_1), 0.90 and 0.90 (rotamers, d, J = 6.7 Hz, 6H).

^{13}C NMR (176 MHz, CDCl$_3$): δ_C = 169.5 and 169.2 (rotamers, C_9), 168.9 and 168.7 (rotamers, C_3), 165.8 and 165.7 (rotamers, C_8), 164.3 and 164.0 (rotamers, C_{10}), 81.8 and 81.6 (rotamers, C_2), 57.6 and 57.6 (rotamers, C_{13}), 56.3 and 55.8 (rotamers, C_5), 51.3 and 50.9 (rotamers, C_4), 41.0 and 40.9 (rotamers, C_{11}), [30.6, 30.5, 30.4 (rotamers, C_{12})], 28.2 (C_1), 27.7 and 27.6 (rotamers, C_6), 20.5 and 20.2 (rotamers, C_7).

HRMS (ES+): Calculated for $C_{18}H_{30}ClN_8O_2^+$, 425.2180; found 425.2186.

FT-IR (ATR): ν_{max} 2957, 2932, 2870, 2091, 1741, 1559, 1492, 1455, 1433, 1366, 1314, 1281, 1223, 1186, 1146, 1094, 1020, 970, 911, 841, 801, 729, 648, 560.

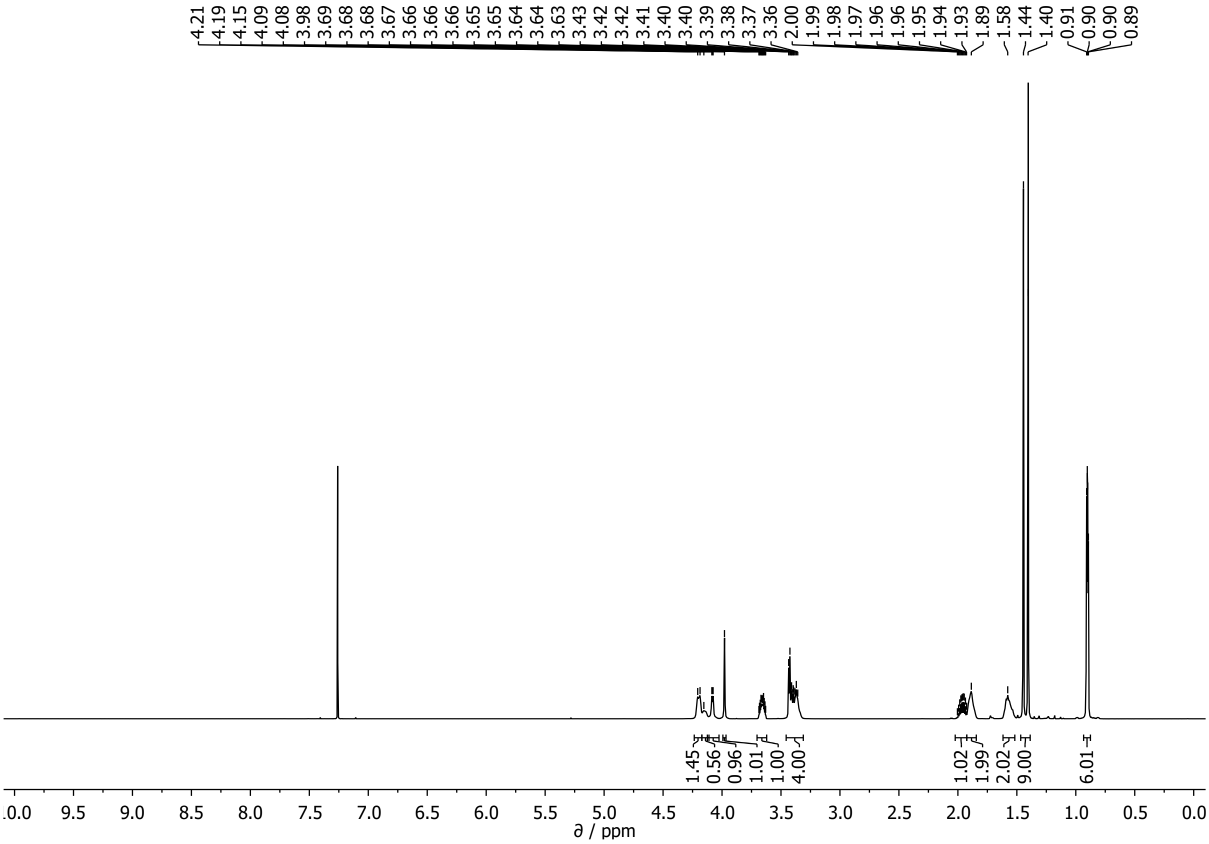

Figure S28 ^{1}H NMR spectrum (700 MHz, $CDCl_3$, 298 K) of **13**.

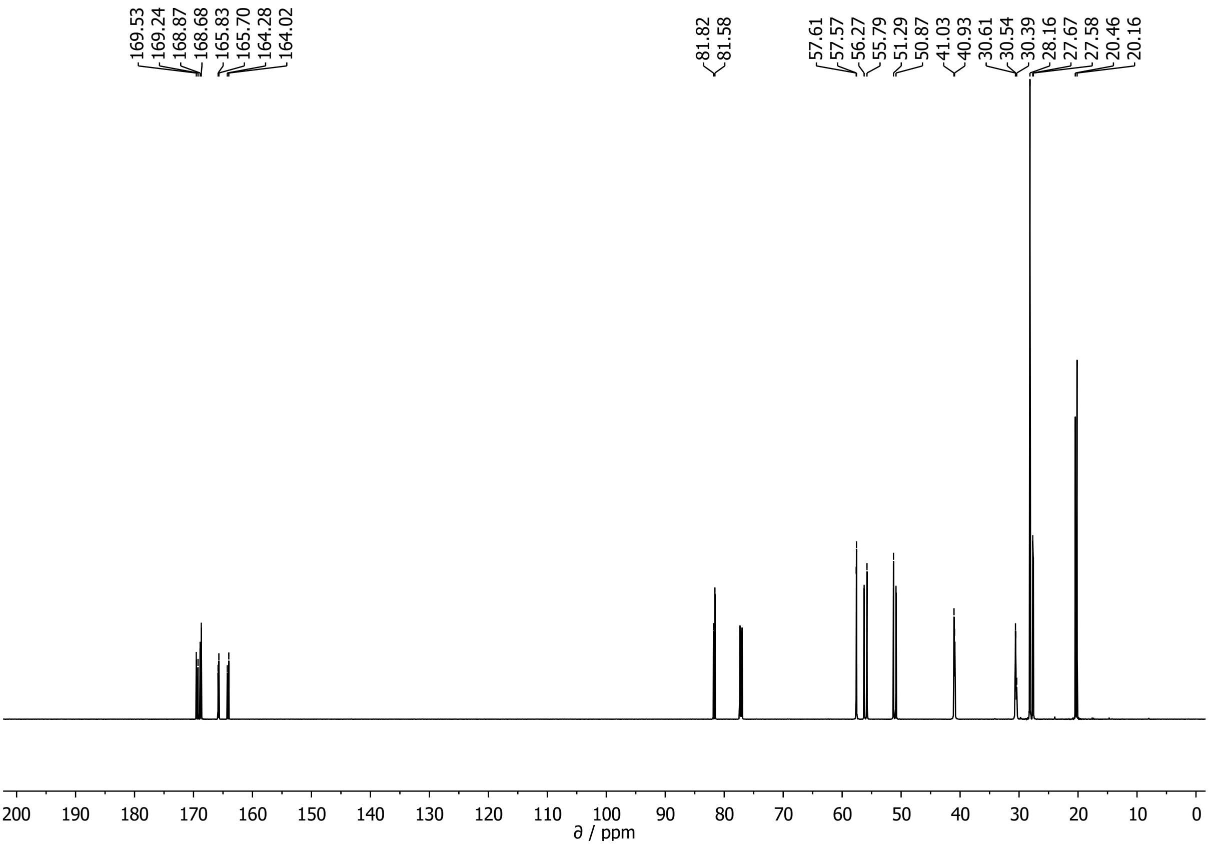

Figure S29 ^{13}C NMR spectrum (176 MHz, $CDCl_3$, 298 K) of **13**.

Synthesis of 14

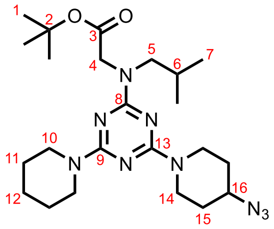

To a solution of **13** (561 mg, 1.32 mmol, 1.0 eq.) in THF (8 mL), piperidine (2.61 mL, 26.4 mmol, 20 eq.) was added. The solution was heated at 70 °C under microwave irradiation for 20 minutes. The solvent was removed under reduced pressure and the obtained residue was redissolved in ethyl acetate (30 mL). The organic layer was washed with hydrochloric acid (1 M, 3 x 30 mL), dried with magnesium sulphate and the solvent removed under reduced pressure. **14** was obtained with no further purification as a colourless oil (625 mg, 1.32 mmol, quantitative).

^{1}H NMR (700 MHz, CDCl$_3$): δ_H = 4.34 (m, 2H, H_{14}), 4.00 and 3.99 (rotamers, s, 2H, H_4), 3.69 and 3.66 (rotamers, t, J = 5.5 Hz, 4H, H_{10}), 3.57 (m, 1H, H_{16}), 4.40 and 3.39 (rotamers, d, J = 4.2 Hz, 2H, H_5), 3.19 (m, 2H, H_{14}), 1.97 (m, 1H, H_6), 1.87 (br, 2H, H_{15}), 1.60 (br, 2H, H_{12}), 1.54 (d, J = 18.0 Hz, 1H), 1.57 – 1.47 (br, 6H, $H_{11,15}$), 1.41 (s, 9H, H_1), 0.90 (d, J = 6.7 Hz, 6H, H_7).

^{13}C NMR (176 MHz, CDCl$_3$): δ_C = 170.3 and 170.3 (rotamers, C_3), 166.2 (C_8), [165.2, 165.2, 165.0, 164.9 ($C_{9/13}$)], 80.8 (C_2), 58.5 and 58.5 (rotamers, C_{16}), 56.0 (C_5), 51.5 and 51.4 (rotamers, C_4), 44.2 and 44.0 (rotamers, C_{10}), 41.0 and 40.9 (rotamers, C_{14}), 30.8 and 30.7 (rotamers, C_{15}), 28.2 (C_1), 28.2 and 28.1(rotamers, C_6) , 25.9 and 25.9 (rotamers, C_{11}), 25.2 and 25.1 (rotamers, C_{12}), 20.6 (C_7).

HRMS (ES+): Calculated for $C_{23}H_{40}N_9O_2^+$, 474.3305; found 474.3308.

FT-IR (ATR): ν_{max} 3002, 2931, 2853, 2091, 1745, 1529, 1481, 1457, 1440, 1366, 1312, 1287, 1250, 1212, 1149, 1100, 1019, 992, 980, 909, 851, 807, 730, 647, 558, 499, 457.

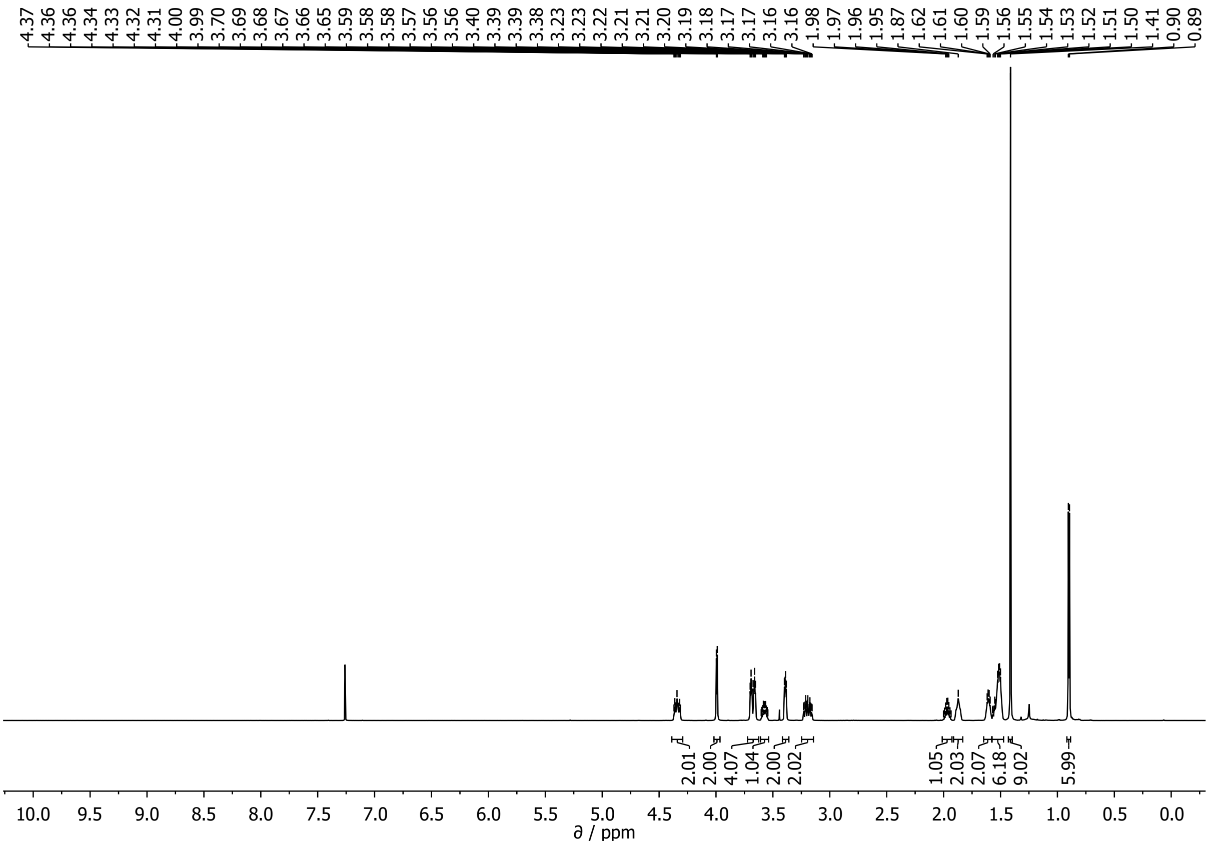

Figure S30 ^{1}H NMR spectrum (700 MHz, $CDCl_3$, 298 K) of **14**.

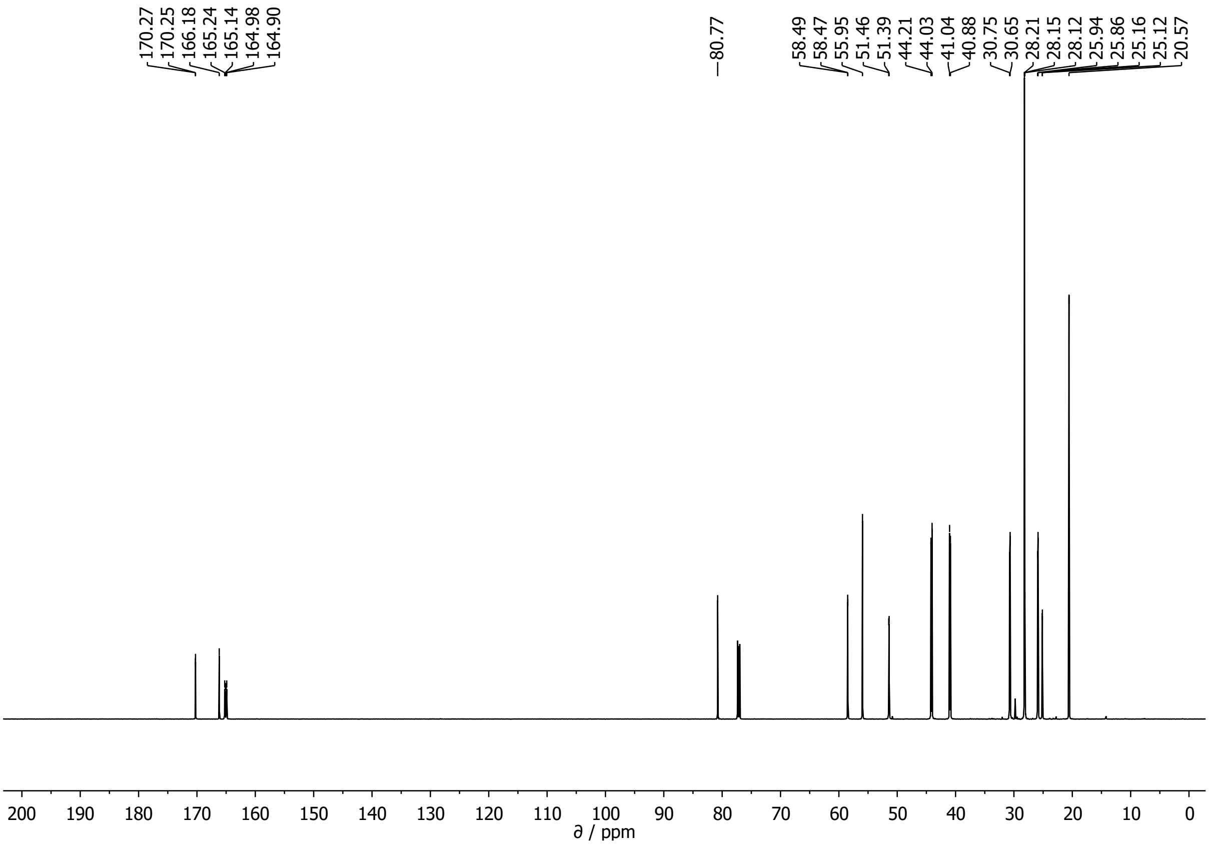

Figure S31 ^{13}C NMR spectrum (176 MHz, $CDCl_3$, 298 K) of **14**.

Synthesis of 15

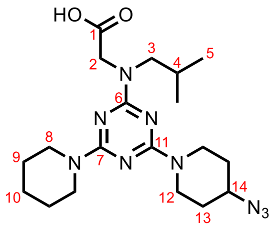

To a solution of **14** (625 mg, 1.32 mmol, 1 eq.) in DCM (10 mL), TFA (10 mL) was added and the solution was stirred at room temperature overnight. The solvent was removed under reduced pressure and the obtained residue was redissolved in ethyl acetate (20 mL). The organic layer was washed with citric acid solution (3 x 20 mL), dried with magnesium sulphate and the solvent removed under reduced pressure. **15** was obtained without further purification as a white foam (529 mg, 1.27 mmol, 96%).

^{1}H NMR (700 MHz, THF-*d₈*): δ_H = 4.94 (br s, 1H, COOH), 4.36 (m, 2H, H_{12}), 4.10 and 4.09 (rotamers, s, 2H, H_2), 3.72 and 3.68 (rotamers, t, J = 5.8 Hz, 4H, H_8), 3.65 (m, 1H, H_{14}), 3.42 (d, J = 7.5 Hz, 2H, H_3), 3.22 and 3.17 (rotamers, ddd, J = 13.0, 9.7, 3.0 Hz, 2H, H_{12}), 2.02 (non, J = 6.8 Hz, 1H, H_4), 1.85 (m, 2H, H_{13}), 1.63 (m, 2H, H_{10}), 1.54 – 1.41 (br, 6H, $H_{9,13}$), 0.91 (d, J = 6.7 Hz, 6H, H_5).

^{13}C NMR (176 MHz, THF-*d₈*): δ_C = 172.0 (C_1), 167.3 (C_6), [166.2, 166.2, 166.0, 165.9 ($C_{7,11}$)], 59.5 and 59.5 (rotamers, C_{14}), 56.5 and 56.4 (rotamers, C_3), 50.6 and 50.5 (rotamers, C_2), 45.0 (C_8), 41.9 and 41.9 (rotamers, C_{12}), 31.6 (C_{13}), 28.9 (C_4), 26.9 (C_9), 26.1 (C_{10}), 20.9 (C_5).

HRMS (ES+): Calculated for $C_{19}H_{32}N_9O_2^+$, 418.2673; found 418.2694.

FT-IR (ATR): ν_{max} 3000 (br), 2929, 2855, 2092, 1720, 1615, 1532, 1485, 1441, 1373, 1287, 1250, 1198, 1137, 1100, 1019, 807.

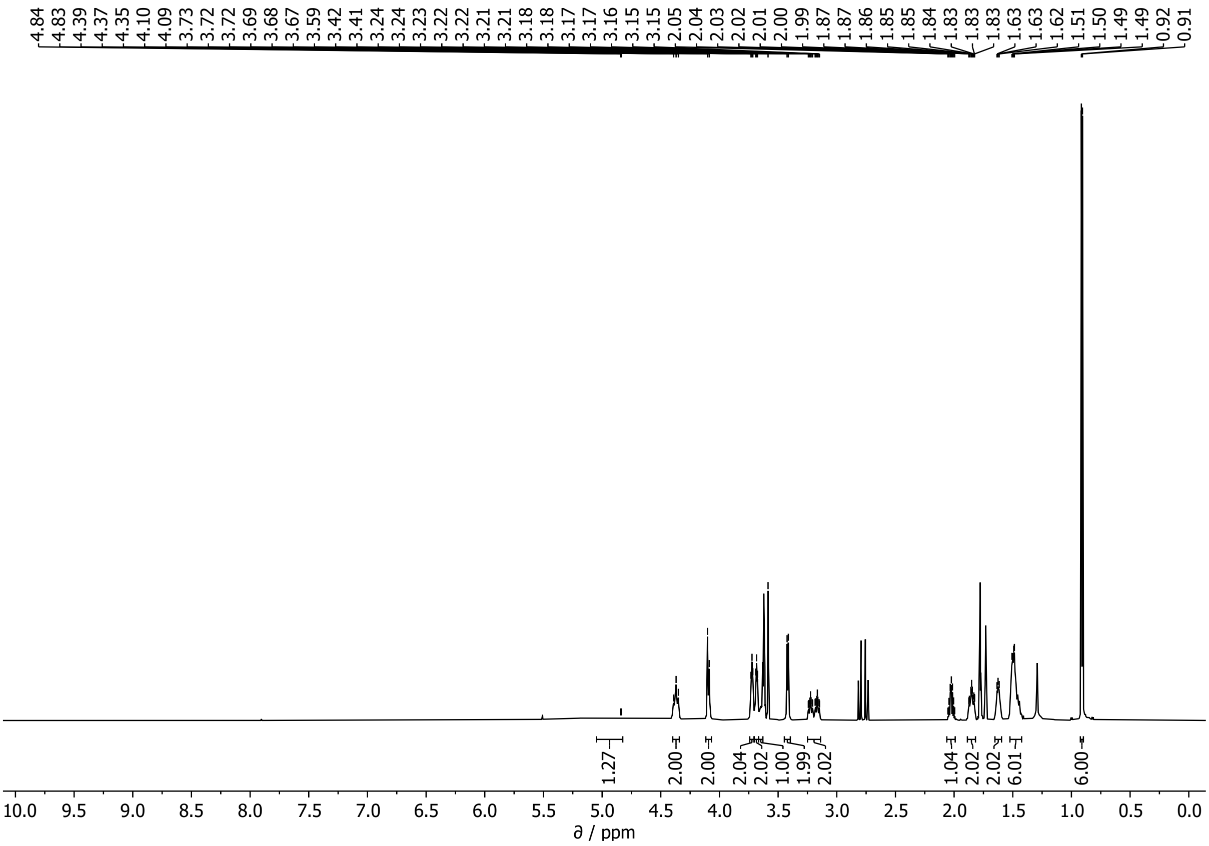

Figure S32 ^{1}H NMR spectrum (700 MHz, THF-d_8, 298 K) of **15**.

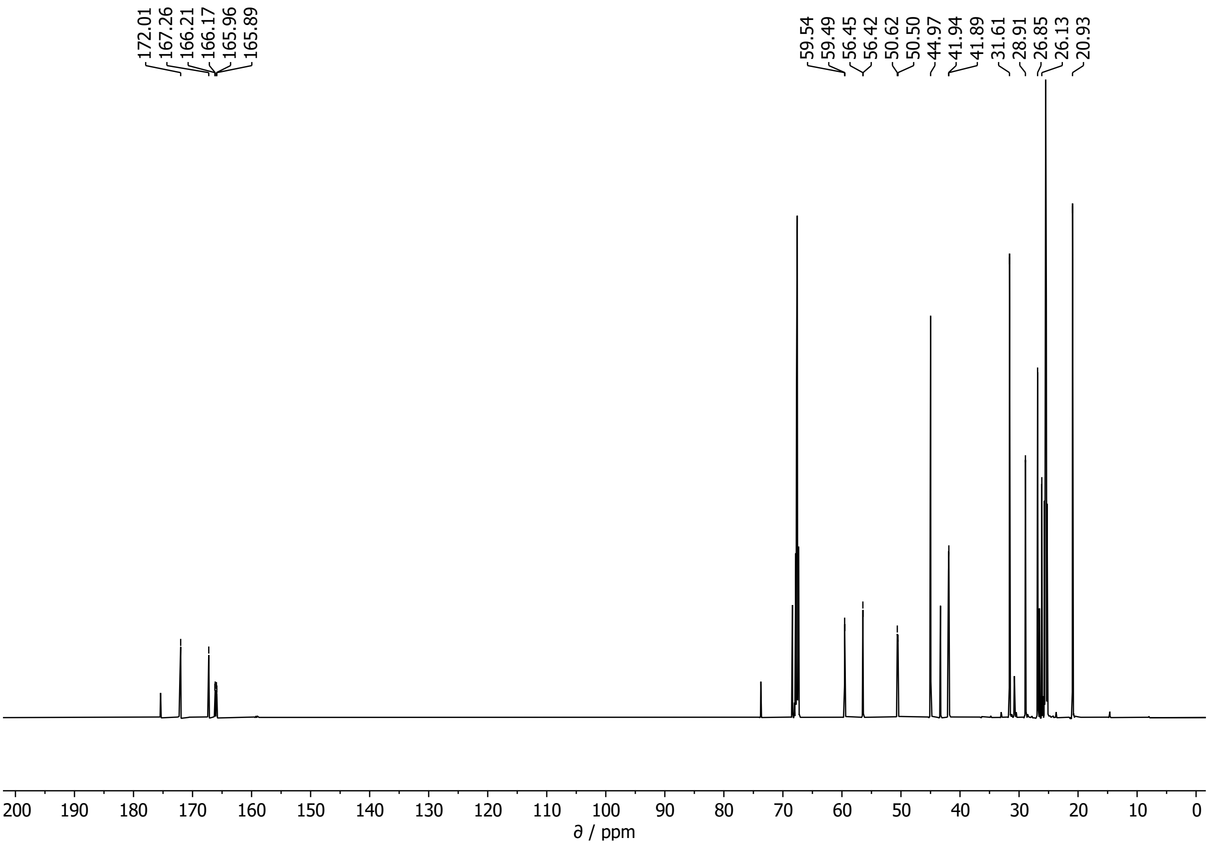

Figure S33 ^{13}C NMR spectrum (176 MHz, THF-d_8, 298 K) of **15**.

Synthesis of 16a

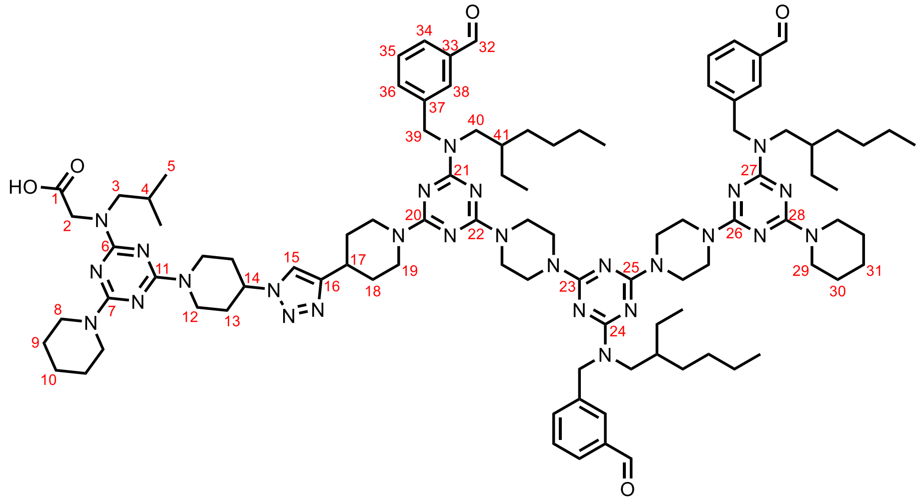

To a solution of **10a** (196 mg, 0.147 mmol, 1.0 eq.) in dry THF (17 mL), **15** (61.4 mg, 0.147 mmol, 1.0 eq.), tetrakis(acetonitrile)copper(I) hexafluorophosphate (126 mg, 0.338 mmol, 2.3 eq.) and TBTA (179 mg, 0.338 mmol, 2.3 eq.) were added. The solution was stirred at room temperature overnight under an atmosphere of nitrogen. The solvent was removed under reduced pressure and the obtained residue was redissolved in ethyl acetate (30 mL). The organic layer was washed with water (30 mL) and EDTA solution (0.01 M, 30 mL), dried with magnesium sulphate and the solvent removed under reduced pressure. The obtained residue was purified by silica flash chromatography (DCM/ ethyl acetate (10 – 80%)). **16a** was obtained as a white foam (248 mg, 0.142 mmol, 96%).

^{1}H NMR (500 MHz, $CDCl_3$): δ_H = [9.98, 9.96 (s, 3H, H_{32})], [7.77, 7.74, 7.72 (br, 6H, $H_{34,38}$)], 7.52 (br, 3H, H_{36}), 7.43 (br, 3H, H_{35}), 7.27 (s, 1H, H_{15}), 4.96 – 4.65 (br, 11H, $H_{8,12,14,19,29,39}$ and protons of piperazine rings), 4.14 (s, 2H, H_2), 3.92 – 3.53 (br, 24H, $H_{8,12,19,29}$ and protons of piperazine rings), 3.52 – 3.37 (br, 8H, $H_{3,40}$), 3.09 – 2.81 (br, 5H, $H_{8,12,17,19,29}$ and protons of piperazine rings), 2.22 (br, 2H, H_{13}), 2.03 (br, 3H, $H_{4,18}$), 1.93 (br, 2H, H_{13}), 1.77 (br, 3H, H_{41}), 1.25 (br, 24H, CH_2 protons of alkyl chains), 0.89 (d, J = 6.6 Hz, 6H, H_5), 0.84 (br, 18H, methyl protons of alkyl chains).

^{13}C NMR (126 MHz, $CDCl_3$): δ_C = [192.6, 192.5 (C_{32})], 172.5 (C_1), 166.3 ($C_{21,24,27}$), 165.6 (C_6), [165.5, 165.2, 165.2, 165.1 ($C_{7,11,20,22,23,25,26,28}$)], [152.3, 152.2 (rotamers, C_{16})], [141.2, 141.1, 141.0 (C_{37})], 136.6 (C_{33}), [133.8, 133.8 (C_{36})], [129.1, 129.1, 129.0, 129.0 (C_{35})], [128.8, 128.7, 128.7, 128.6 (C_{38})], [128.5, 128.3, 128.2 (C_{34})], [117.3, 117.2 (rotamers, C_{15})], 58.4 (C_{14}), 56.9 (C_3), 53.4 (C_2), 50.1 (C_{40}), 50.0 (C_{39}), [44.6, 44.2, 44.2, 43.4, 43.2, 42.4 ($C_{8,12,19,29}$ and carbons of piperazine rings)], [38.0, 38.0 (C_{41})], [34.3, 34.2 (rotamers, C_{17})], 32.5 (C_{13}),

[32.1, 32.0, 31.9 (rotamers, C_{18})] , [30.8, 29.8, 29.8, 29.8, 29.7, 29.6, 29.5, 29.4, 29.2, 29.1 (CH_2 carbons of alkyl chain)], 27.9 (C_4), [25.9, 25.8, 25.2, 25.1, 24.9 ($C_{9,10,30,31}$)], [24.0, 23.3, 22.8 (CH_2 carbons of alkyl chain)], 20.3 (C_5), [14.3, 14.2, 10.9 (methyl carbons of alkyl chain)].

HRMS (ES+): Calculated for $C_{96}H_{140}N_{27}O_5^+$, 1751.1525; found 1751.1546.

FT-IR (ATR): ν_{max} 2954, 2925, 2900 (br), 2853, 1735, 1700, 1525, 1480, 1431, 1369, 1351, 1302, 1252, 1183, 1136, 1099, 994, 910, 851, 807, 733, 651.

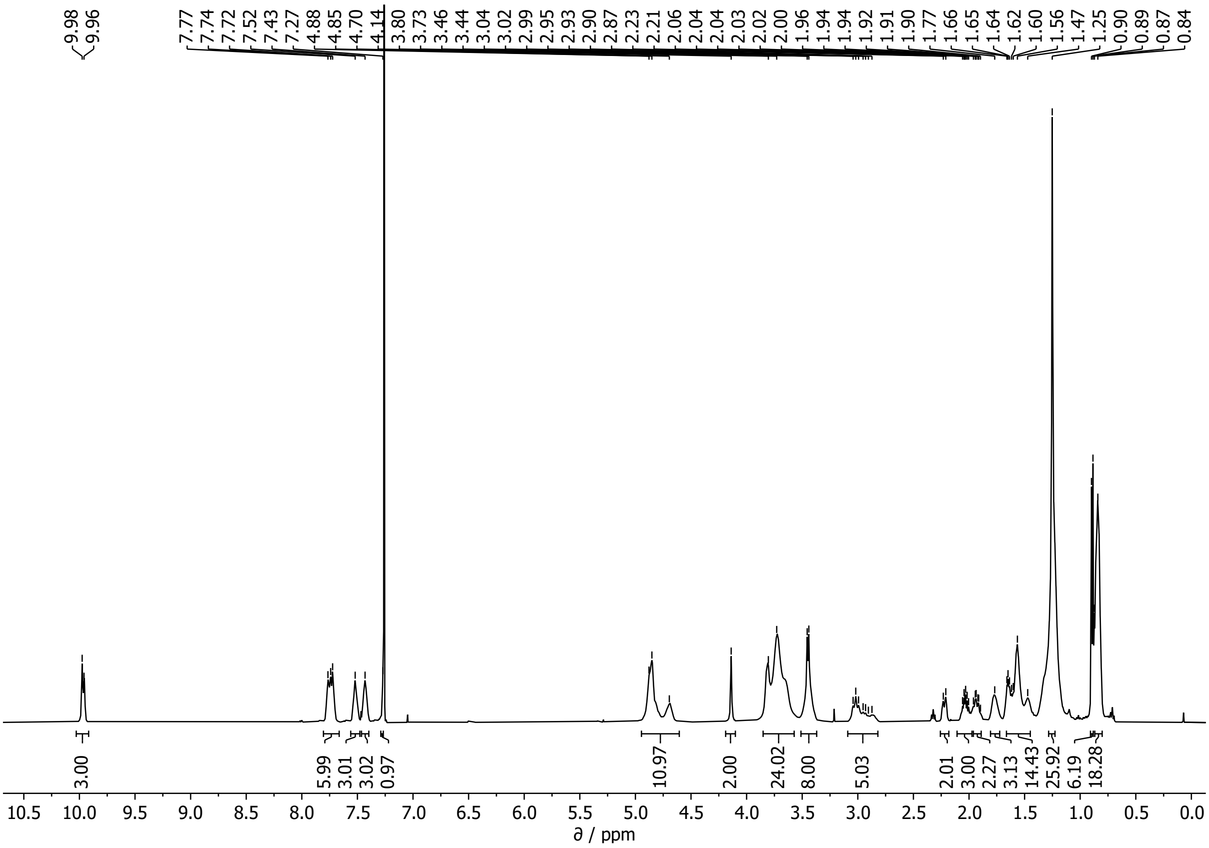

Figure S34 ^1H NMR spectrum (500 MHz, $CDCl_3$, 298 K) of **16a**.

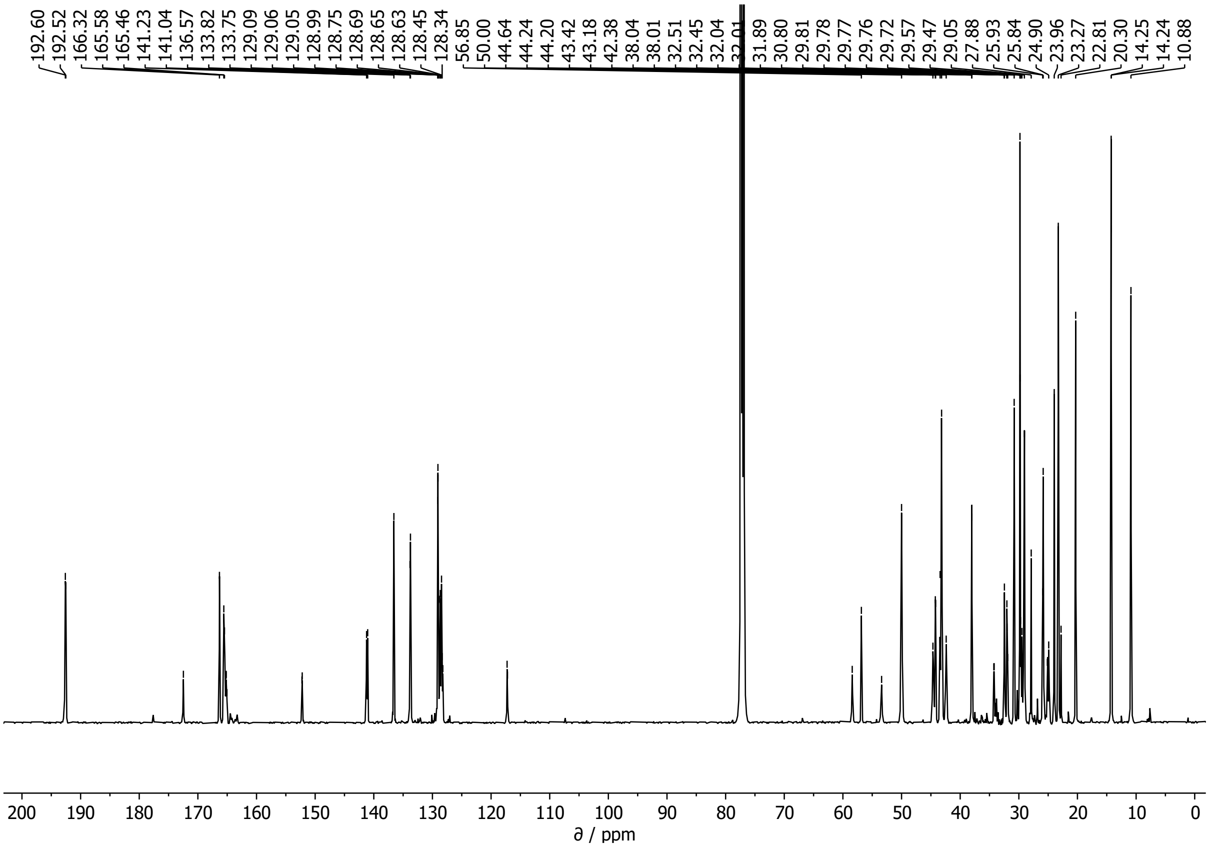

Figure S35 ^{13}C NMR spectrum (126 MHz, $CDCl_3$, 298 K) of **16a**.

Synthesis of 16b

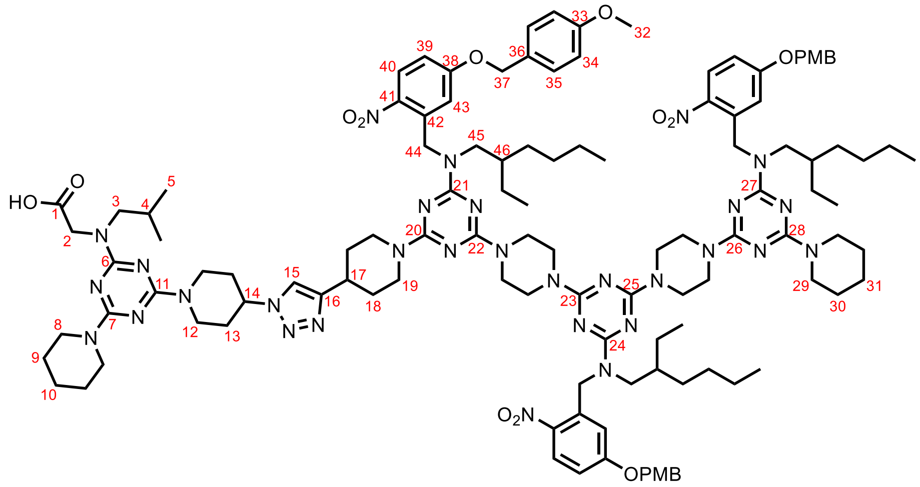

To a solution of **10b** (578 mg, 0.322 mmol, 1.0 eq.) in dry THF (40 mL), **15** (134 mg, 0.322 mmol, 1.0 eq.), tetrakis(acetonitrile)copper(I) hexafluorophosphate (175 mg, 0.468 mmol, 1.5 eq.) and TBTA (248 mg, 0.468 mmol, 1.5 eq.) were added. The solution was stirred at room temperature overnight under an atmosphere of nitrogen. The solvent was removed under reduced pressure and the obtained residue was redissolved in ethyl acetate (30 mL). The organic layer was washed with water (30 mL) and EDTA solution (0.01 M, 30 mL), dried with magnesium sulphate and the solvent removed under reduced pressure. The obtained residue was purified by silica flash chromatography (DCM/ ethyl acetate (10 – 80%)). **16b** was obtained as a white foam (541 mg, 0.245 mmol, 76%).

^{1}H NMR (500 MHz, CDCl$_3$): δ_H = 8.11 (br, 3H, H_{40}), 7.25 (br, 7H, $H_{15,35}$), 6.86 (br, 9H, $H_{34,39}$), 6.76 (br, 3H, H_{43}), 5.11 (br, 6H, H_{44}), 4.98 (br, 6H, H_{37}), 4.91 – 4.41 (br, 5H, H_{14}, $H_{8/12/19/29}$ and protons of piperazine rings), 4.14 (br, 2H, H_2), 3.91 – 3.15 (br, 41H, $H_{3,8,29,32,45}$ and protons of piperazine rings), 3.12 – 2.60 (br, 5H, H_{17}, $H_{8/12/19/29}$ and protons of piperazine rings), 2.21 (br, 2H, H_{13}), 2.09 (br, 2H, H_{18}), 2.03 (br, 1H, H_4), 1.94 (br, 2H, H_{13}), 1.80 (br, 3H, H_{46}), 1.71 – 1.48 (br, 10H, $H_{9,10,18,30,31}$), 1.36 (br, 4H, $H_{9,30}$), 1.27 (br, 24H, CH_2 protons of alkyl chains), 0.87 (br, 24H, H_5 and methyl protons of alkyl chains).

^{13}C NMR (126 MHz, CDCl$_3$): δ_C = [166.2, 166.2 ($C_{6,21,24,27}$)], [165.6, 165.5, 165.5, 165.4, 165.4, 165.3, 165.2, 165.2, 165.1, 165.1, 165.0, 164.9 ($C_{1,7,11,20,22,23,25,26,28}$)], [162.9, 162.9 ($C_{38}$)], [159.8, 159.7 ($C_{33}$)], 152.0 ($C_{16}$), [141.4, 141.3 ($C_{41}$)], [139.5, 139.4, 139.3 (C_{42})], 129.5 (C_{35}), [127.9, 127.8 (C_{40})], [127.6, 127.6, 127.5 (C_{36})], 117.3 (C_{15}), 114.4 (C_{43}), 114.2 (C_{34}), [112.8, 112.6 (C_{39})], 70.3 (C_{37}), 58.4 (C_{14}), 56.7 (C_3), [55.3, 55.3 (C_{32})], 53.4 (C_2), 50.9 (C_{45}), 48.9 (C_{44}), [44.6, 44.2, 44.1, 43.2, 43.1, 42.9, 42.3 ($C_{8,12,19,29}$ and carbons of piperazine rings)],

[38.3, 38.2 (C_{46})], [34.2, 34.1 (rotamers, C_{17})], 32.4 (C_{13}), [32.0, 32.0, 32.0 (rotamers, C_{18})], 31.7 (C_{13}), [30.7, 29.8, 28.9, 28.9 (CH_2 carbons of alkyl chains)], 27.8 (C_4), [26.0, 25.9, 25.8, 25.7 ($C_{9,30}$)], [25.1, 25.0, 24.9 ($C_{10,31}$)], [23.9, 23.3 (CH_2 carbons of alkyl chains)], 23.2 (C_5), [14.2, 14.2, 10.8, 10.8 (methyl carbons of alkyl chains)].

HRMS (ES+): Calculated for $C_{117}H_{161}N_{30}O_{14}^{+}$, 2210.2803; found 2210.2905.

FT-IR (ATR): ν_{max} 3000 (br), 2998, 2954, 2927, 2853, 1731, 1612, 1578, 1528, 1479, 1433, 1370, 1350, 1317, 1283, 1252, 1177, 1072, 1034, 995, 909, 844, 807, 733.

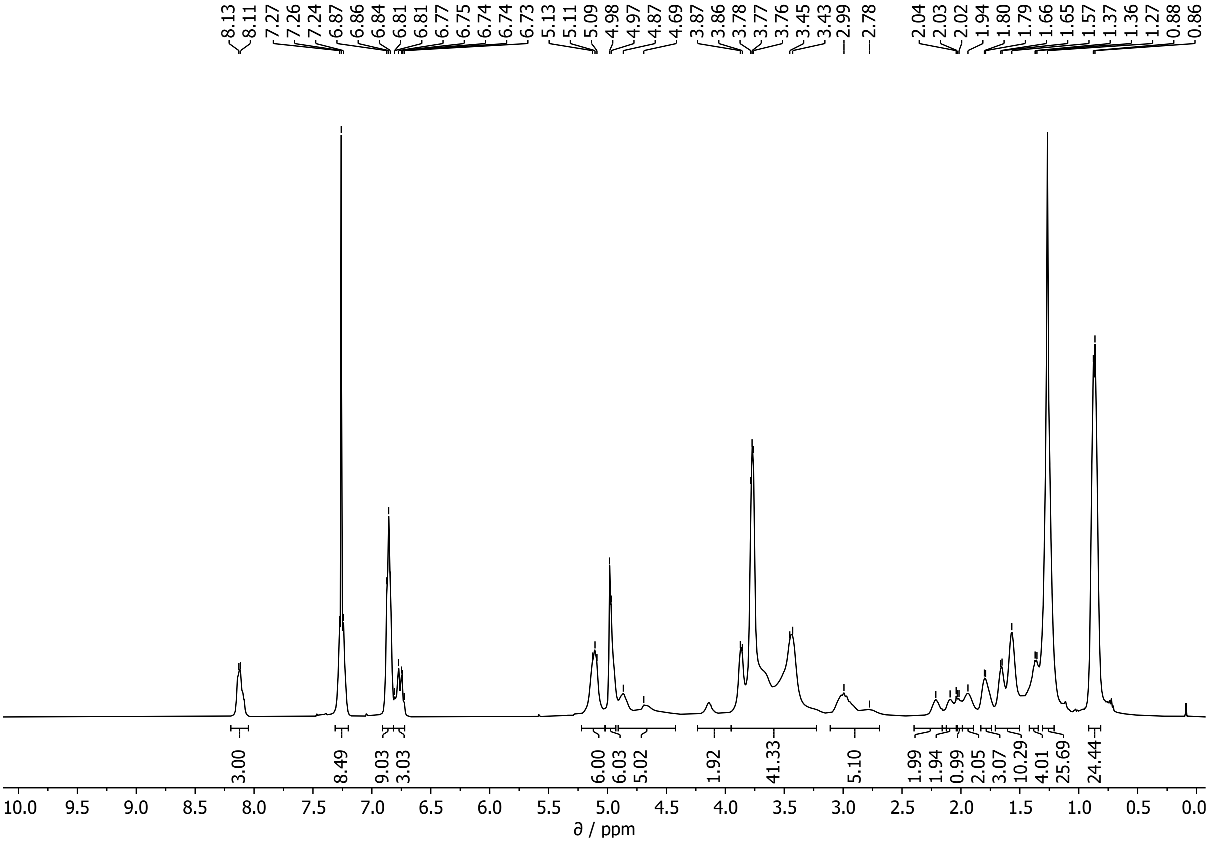

Figure S36 ^{1}H NMR spectrum (500 MHz, $CDCl_3$, 298 K) of **16b**.

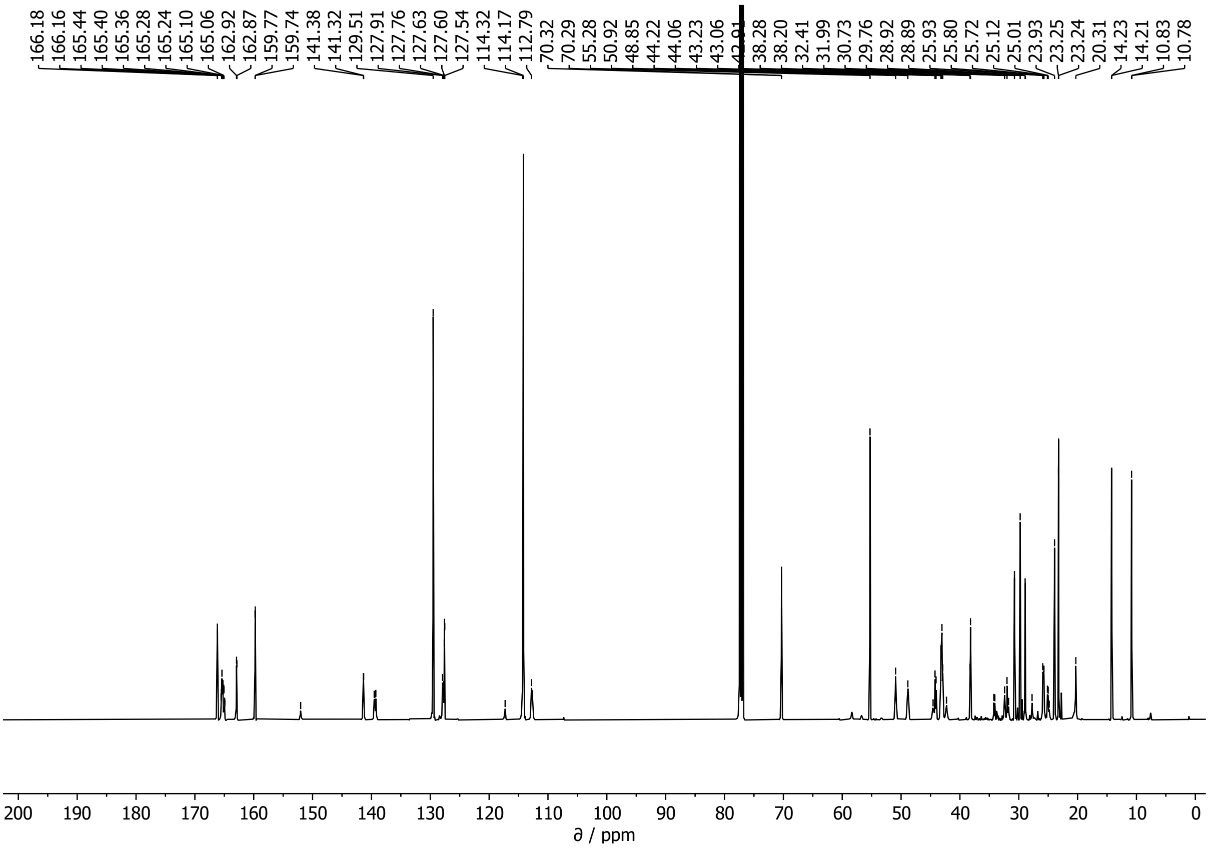

Figure S37 ^{13}C NMR spectrum (126 MHz, $CDCl_3$, 298 K) of **16b**.

Synthesis of 17

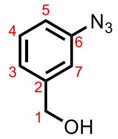

17 was prepared according to a literature procedure.[4,5]

Synthesis of 18

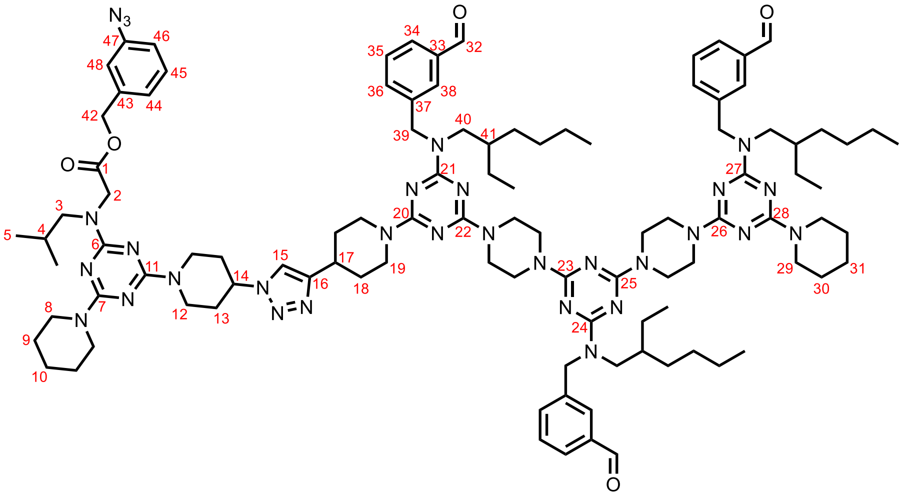

To a solution of **16a** (248 mg, 0.142 mmol, 1 eq.) in dry DCM (10 mL), **17** (21.1 mg, 0.142 mmol, 1 eq.), EDC (136 mg, 0.71 mmol, 5 eq.) and DMAP (173 mg, 1.42 mmol, 10 eq) were added and the solution was stirred at room temperature overnight under nitrogen. The solution was diluted with DCM (10 mL) and washed with hydrochloric acid solution (0.1 M, 3 x 20 mL). The organic layer was dried over magnesium sulphate and the solvent was removed under reduced pressure. The obtained residue was purified by silica flash chromatography (DCM / ethyl acetate (8:2)). **18** was obtained as a yellow oil (177 mg, 0.0940 mmol, 66%).

^{1}H NMR (700 MHz, CDCl$_3$): δ_H = [9.98, 9.96 (s, 3H, H_{32})], [7.77, 7.74, 7.72 (br, 6H, $H_{34,38}$)], 7.53 (br, 3H, H_{36}), 7.43 (br, 3H, H_{35}), [7.30, 7.27 (rotamers, t, J = 7.7 Hz, 1H, H_{45})], [7.27, 7.21 (rotamers, s, 1H, H_{15})], [7.07, 7.03 (rotamers, d, J = 7.6 Hz, 1H, H_{44})], [6.96, 6.93 (rotamers, d, J = 7.6 Hz, 1H, H_{46})], [6.95, 6.92 (rotamers, s, 1H, H_{48})], [5.11, 5.10 (rotamers, s, 2H, H_{42})], 4.93 – 4.70 (br, 10H, $H_{8,12,19,29,39}$ and protons of piperazine rings), [4.69, 4.61 (rotamers, br, 1H, H_{14})], [4.17, 4.16 (rotamers, s, 2H, H_2)], 3.89 – 3.53 (br, 24H, $H_{8,12,19,29}$ and protons of piperazine rings), 3.52 – 3.38 (br, 8H, $H_{3,40}$), 3.08 – 2.74 (br, 5H, $H_{8,12,17,19,29}$ and protons of piperazine rings), [2.19, 2.18 (rotamers, br, 1H, H_{13})], 2.07 (br, 1H, H_{18}), 1.99 (non, J = 6.9 Hz, 1H, H_4), 1.89 (br, 1H, H_{13}), 1.83 – 1.70 (br, 5H, $H_{13,41}$), 1.67 – 1.40 (br, 14H, $H_{9,10,18,30,31}$),

[1.25, 1.24 (br, 24H, CH_2 protons of alkyl chains), [0.92, 0.91 (rotamers, d, J = 5.9 Hz, 6H, H_5), 0.84 (br, 18H, methyl protons of alkyl chains).

^{13}C NMR (176 MHz, $CDCl_3$): δ_C = [192.6, 192.5 (C_{32})], [170.9, 170.9 (rotamers, C_1)], [166.4, 166.1 ($C_{6,21,24,27}$)], [165.6, 165.5, 165.3, 165.2, 165.1, 164.9, 164.8 ($C_{7,11,20,22,23,25,26,28}$)], 152.1 ($C_{16}$), [141.3, 141.2, 141.2, 141.2 (C_{37})], [140.5, 140.4 (rotamers, C_{47})], 138.1 (C_{43}), 136.6 (C_{33}), [133.8, 133.7 (C_{36})], 130.0 (C_{45}), [129.1, 129.1 (C_{35})], 128.7 (C_{38}), [128.5, 128.3, 128.2 (C_{34})], [124.4, 124.4 (rotamers, C_{44})], 118.8 (C_{46}), [118.5, 118.4 (rotamers, C_{48})], [117.1, 117.0 (rotamers, C_{15})], [65.7, 65.6 (rotamers, C_{42})], [58.8, 58.8 (rotamers, C_{14})], 55.9 (C_3), [50.8, 50.7 (rotamers, C_2)], 50.0 ($C_{39,40}$), [44.3, 44.2, 44.1, 43.4, 43.2, 42.2, 42.0 ($C_{8,12,19,29}$ and carbons of piperazine rings)], 38.0 (C_{41}), [34.3, 34.2 (rotamers, C_{17})], [32.6, 32.5 (rotamers, C_{13})], [32.0, 32.0, 31.9 (rotamers, C_{18})], [30.8, 29.8, 29.1 (CH_2 carbons of alkyl chains)], [28.2, 28.2 (rotamers, C_4)], [25.9, 25.8 (rotamers, $C_{9,10,30,31}$)], [25.2, 25.1 (rotamers, $C_{9,10,30,31}$)], [24.0, 23.3, 21.2, 20.5 (CH_2 carbons of alkyl chains)], [14.3, 14.3, 10.9 (C_5 and methyl carbons of alkyl chains)].

HRMS (ES+): Calculated for $C_{103}H_{145}N_{30}O_5^+$, 1882.2014; found 1882.2034.

FT-IR (ATR): ν_{max} 2952, 2927, 2854, 2114, 1747, 1701, 1530, 1484, 1434, 1370, 1353, 1289, 1253, 1182, 1137, 1099, 995, 807.

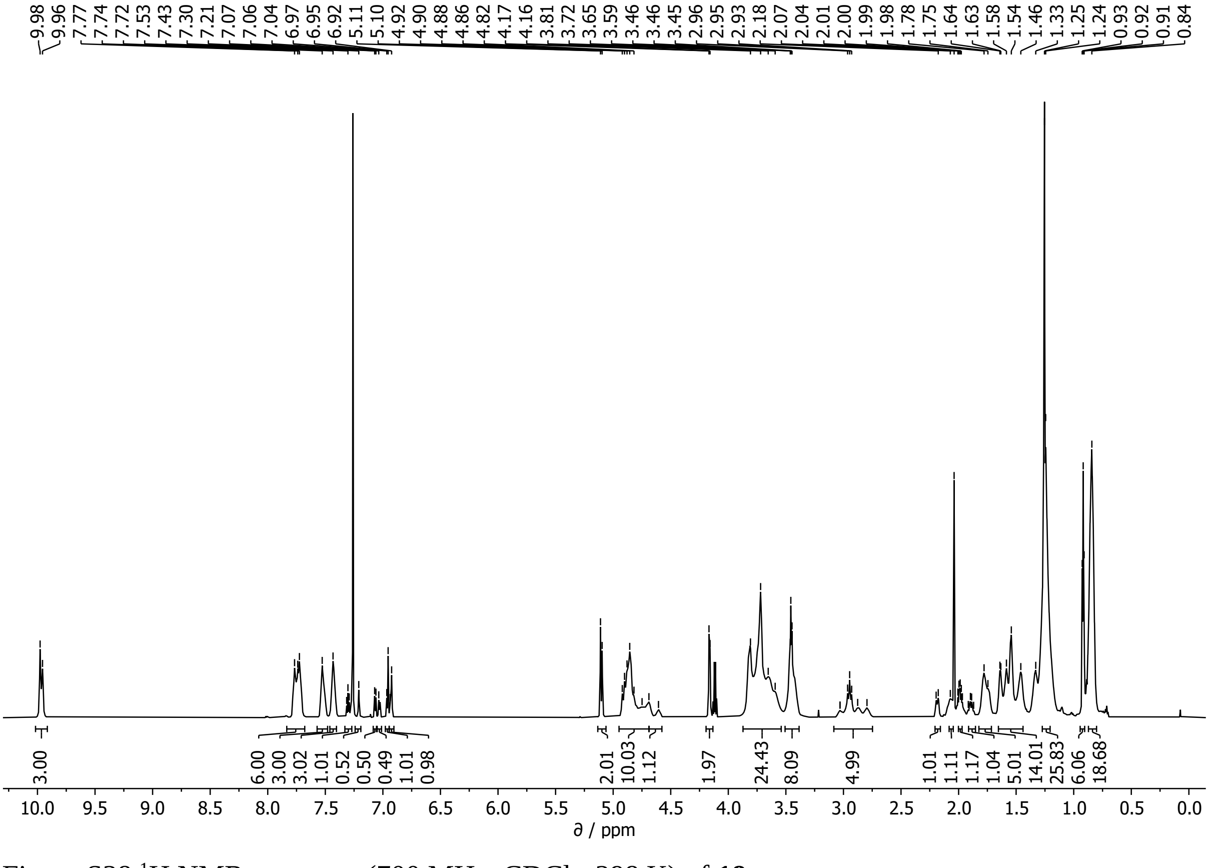

Figure S38 ^{1}H NMR spectrum (700 MHz, $CDCl_3$, 298 K) of **18**.

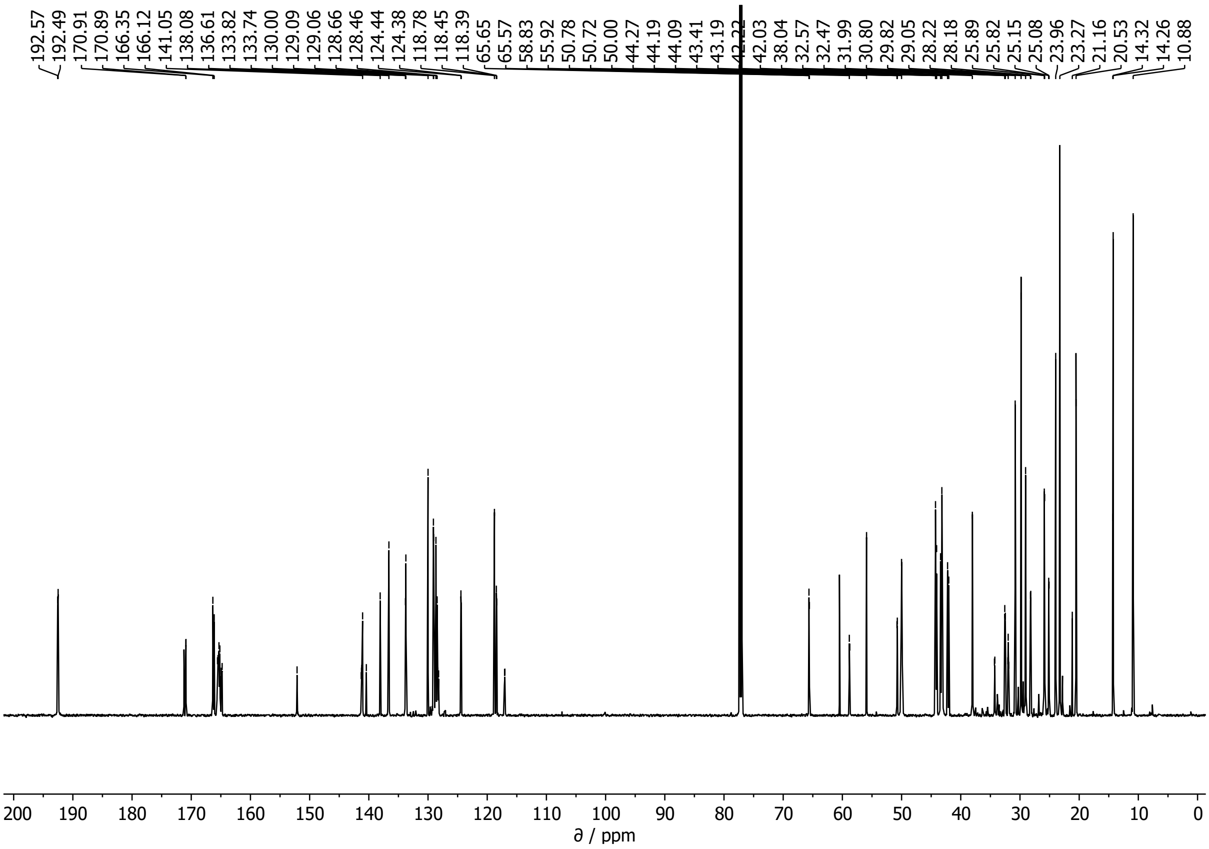

Figure S39 ^{13}C NMR spectrum (176 MHz, $CDCl_3$, 298 K) of **18**.

Synthesis of 19

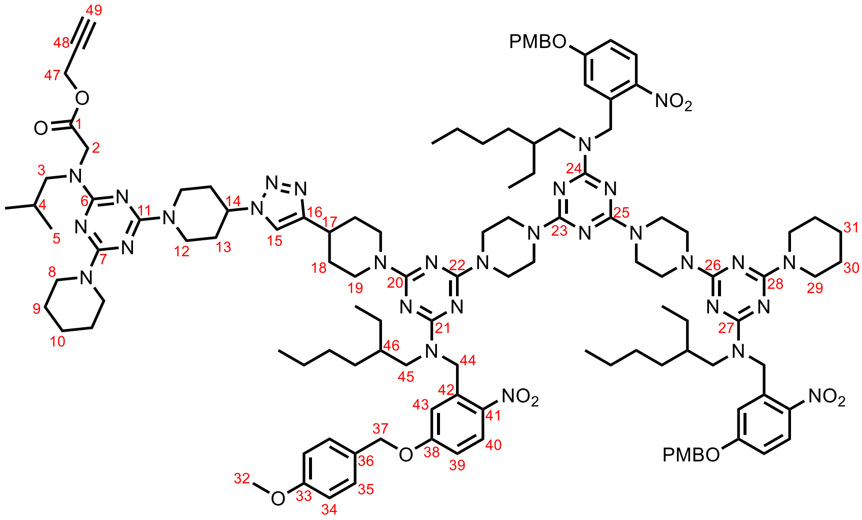

To a solution of **16b** (250 mg, 0.113 mmol, 1 eq.) in dry DCM (10 mL), propargyl alcohol (50 μL, 0.87 mmol, 8 eq.), EDC (108 mg, 0.565 mmol, 5 eq.) and DMAP (138 mg, 1.13 mmol, 10 eq.) were added and the solution was stirred at room temperature overnight under nitrogen. The solution was diluted with DCM (10 mL) and washed with hydrochloric acid solution (0.1 M, 3 x 20 mL). The organic layer was dried over magnesium sulphate and the solvent was removed under reduced pressure. **19** was obtained as an orange oil and used without further purification (252 mg, 0.112 mmol, quantitative).

^{1}H NMR (700 MHz, CDCl$_3$): δ_H = 8.11 (br, 3H, H_{40}), 7.21 (br, 7H, $H_{15,35}$), 6.85 (br, 9H, $H_{34,39}$), 6.76 (br, 3H, H_{43}), 5.11 (br, 6H, H_{44}), [4.98, 4.97 (br, 6H, H_{37})], 4.95 – 4.80 (br, 4H, $H_{8/12/19/29}$ and protons of piperazine rings), 4.69 and 4.69 (rotamers, s, 2H, H_{47}), 4.66 (br, 1H, H_{14}), 4.15 (s, 2H, H_2), 3.92 – 3.15 (br, 41H, $H_{3,8,12,19,29,32,45}$ and protons of piperazine rings), 3.09 – 2.67 (br, 5H, $H_{8,12,17,19,29}$ and protons of piperazine rings), 2.46 and 2.44 (rotamers, br, 1H, H_{49}), 2.17 (br, 2H, H_{13}), 2.09 (br, 1H, H_{18}), 2.00 (non, J = 6.8 Hz, 1H, H_4), 1.87 (br, 2H, H_{13}), 1.79 (br, 3H, H_{46}), 1.70 – 1.49 (br, 15H, $H_{9,10,18,30,31}$), 1.44 – 1.15 (br, 24H, CH_2 protons of alkyl chains), 0.92 (m, 6H, H_5), 0.86 (br, 18H, methyl protons of alkyl chains).

^{13}C NMR (176 MHz, CDCl$_3$): δ_C = [170.4, 170.4 (rotamers, C_1)], [166.2, 166.2, 166.1 ($C_{6,21,24,27}$)], [165.6, 165.6, 165.5, 165.5, 165.5, 165.4, 165.3, 165.3, 165.2, 165.0, 165.0, 164.8 ($C_{7,11,20,22,23,25,28,28}$)], [163.0, 162.9 ($C_{38}$)], [159.8, 159.8 ($C_{33}$)], [152.1, 152.1 (rotamers, C_{16})], [141.4, 141.4 (C_{41})], [139.6, 139.5, 139.3 (C_{42})], 129.6 (C_{35}), [128.0, 127.8, 127.7 (C_{40})], [127.7, 127.7, 127.6 (C_{36})], 117.1 (C_{15}), 114.4 (C_{43}), 114.2 (C_{34}), [112.9, 112.8, 112.7 (C_{39})], [77.7, 77.6 (rotamers, C_{48})], [75.1, 75.0 (rotamers, C_{49})], [70.4, 70.3 (C_{37})], [58.8, 58.8 (rotamers, C_{14})], 55.9 (C_3), [55.4, 55.3 (C_{32})], 52.2 (C_{47}), [51.0, 51.0, 50.9 (C_{45})], 50.5 (C_2),

[48.9, 48.8 (C_{44})], [44.3, 44.3, 44.2, 44.1, 43.4, 43.3, 43.1, 43.0 ($C_{8,19,29}$ and carbons of piperazine rings)], [42.2, 42.2 (rotamers, C_{12})], [38.3, 38.3 (C_{46})], [34.3, 34.2 (rotamers, C_{17})], 32.6 (C_{13}), [32.1, 32.0 (rotamers, C_{18})], [31.8, 30.8, 29.0, 28.9 (CH_2 carbons of alkyl chains)], [28.2, 28.2 (rotamers, C_4)], [26.0, 25.9, 25.9, 25.8, 25.2, 25.1, 25.1, 25.1 ($C_{9,10,30,31}$)], [24.0, 23.3 (CH_2 carbons of alkyl chains)], [20.5, 20.5 (rotamers, C_5)], [14.3, 14.2, 10.9, 10.8 (methyl carbons of alkyl chains)].

HRMS (ES+): Calculated for $C_{120}H_{163}N_{30}O_{14}^{+}$, 2248.2960; found 2248.2964.

FT-IR (ATR): ν_{max} 3306, 2956, 2924, 2853, 1758, 1612, 1578, 1526, 1477, 1429, 1370, 1349, 1338, 1316, 1283, 1250, 1175, 1099, 1072, 1023, 994, 909, 843, 807, 732, 651.

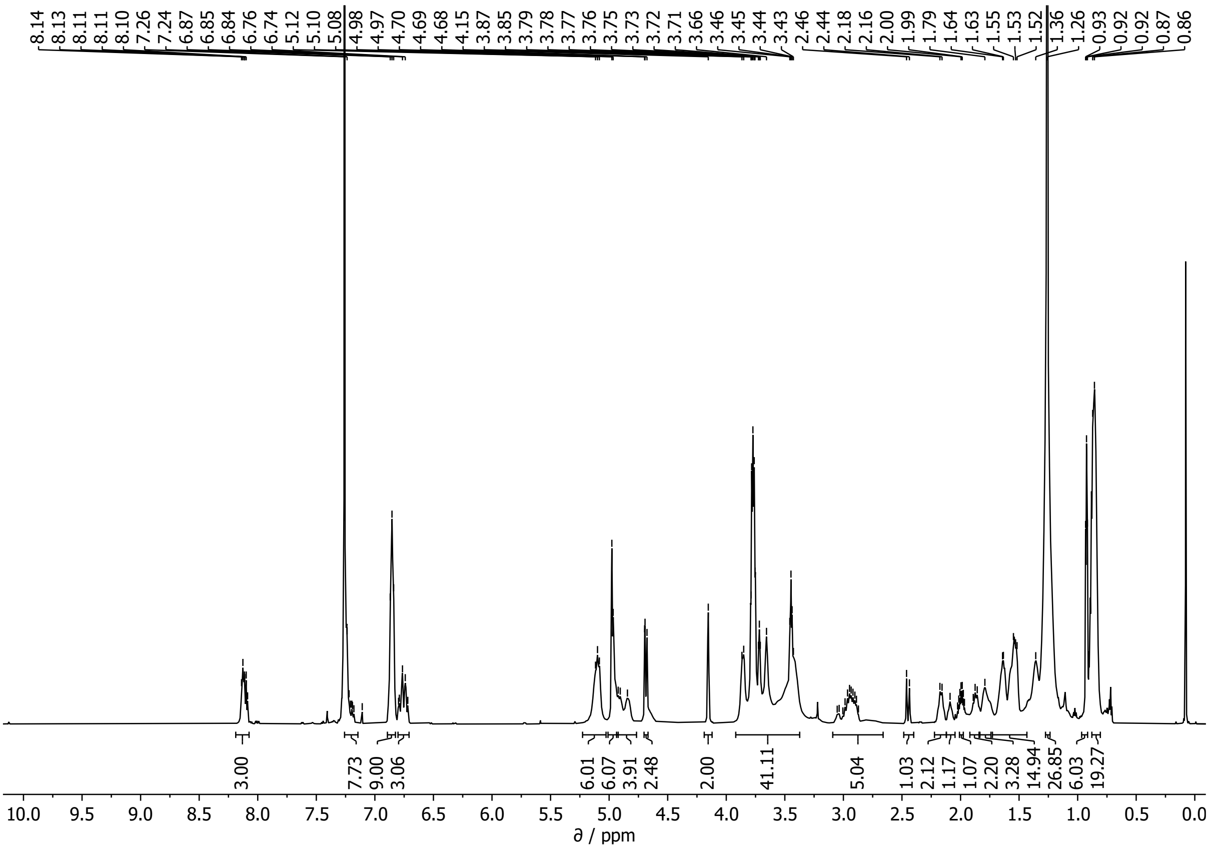

Figure S40 ^{1}H NMR spectrum (700 MHz, $CDCl_3$, 298 K) of **19**.

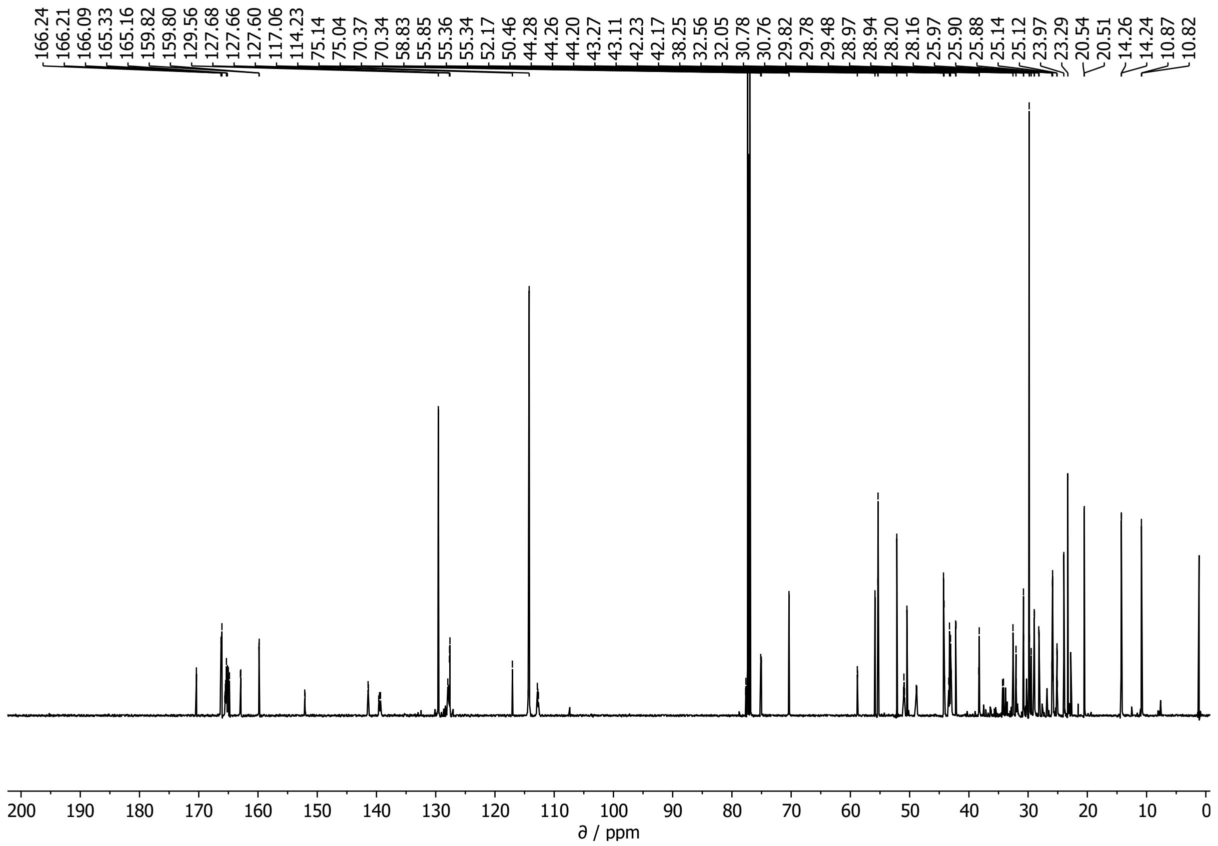

Figure S41 ^{13}C NMR spectrum (176 MHz, $CDCl_3$, 298 K) of **19**.

Synthesis of 20

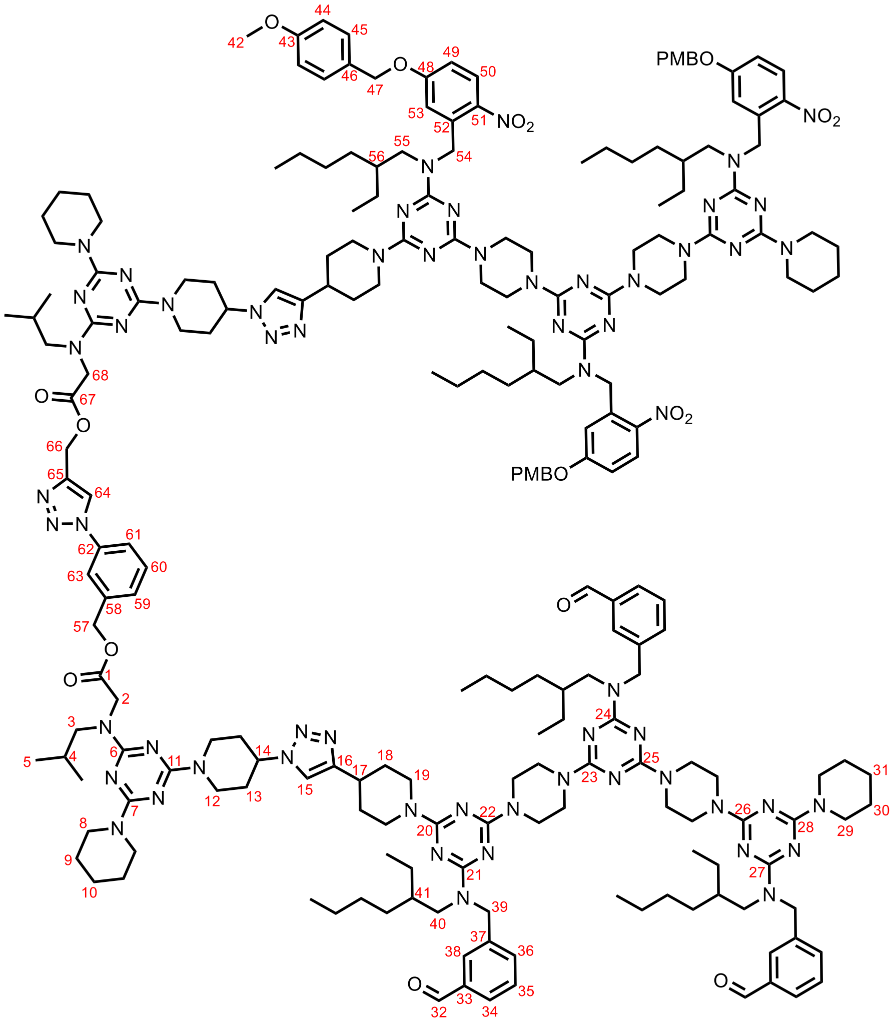

To a solution of **19** (120 mg, 0.0531 mmol, 1.00 eq.) in dry THF (5 mL), **18** (100 mg, 0.0531 mmol, 1.00 eq.), tetrakis(acetonitrile)copper(I) hexafluorophosphate (20 mg, 0.053 mmol, 1.0 eq.) and TBTA (28 mg, 0.053 mmol, 1.0 eq.) were added. The solution was stirred at room temperature overnight under an atmosphere of nitrogen. The solvent was removed under reduced pressure and the obtained residue was redissolved in ethyl acetate (20 mL). The organic layer was washed with water (20 mL) and EDTA solution (0.01 M, 20 mL), dried with magnesium sulphate and the solvent removed under reduced pressure. The obtained residue was purified by silica flash chromatography (DCM / ethyl acetate (20 – 50%)). **20** was obtained as a white foam (186 mg, 0.0450 mmol, 85%).

^{1}H NMR (700 MHz, CDCl$_3$): δ_H = [9.98, 9.96 (s, 3H, H_{32})], 8.10 (br, 3H, H_{50}), [7.95, 7.94 (rotamers, s, 1H, H_{64})], 7.74, (br, 6H, $H_{34,38}$), [7.68, 7.67, 7.66 (rotamers, br, 1H, H_{63})], 7.62 (br, 1H, H_{61}), 7.53 (br, 3H, H_{36}), 7.48 (br, 1H, H_{60}), 7.42 (br, 3H, H_{35}), [7.38, 7.35 (rotamers,

d, J = 7.0 Hz, 1H, H_{59})], 7.27 (s, 2H, H_{15}), 7.26 (br, 6H, H_{45}), 6.85 (br, 9H, $H_{44, 49}$), 6.76 (br, 3H, H_{53}), [5.34, 5.32 (rotamers, s, 2H, H_{66})], [5.21, 5.19 (rotamers, s, 2H, H_{57})], 5.09 (br, 6H, H_{54}), 4.97 (br, 6H, H_{47}), 4.93 – 4.47 (br, 16H, $H_{8,12,14,19,29,39}$ and protons of piperazine rings), 4.19 (s, 2H, $H_{2/68}$), 4.16 (s, 2H, $H_{2/68}$), 3.92 – 3.31 (br, 73H, $H_{3,8,12,19,,29,40,42,55}$ and protons of piperazine rings), 3.08 – 2.63 (br, 10H, $H_{8,12,17,19,29}$ and protons of piperazine rings), 2.18 (br, 2H, H_{13}), 2.06 (br, 3H, H_{13}), 1.99 (br, 4H, $H_{4,18}$), 1.91 (br, 3H, H_{13}), 1.84 – 1.43 (br, 36H, $H_{9,10,18,30,31,41,56}$), 1.26 (br, 48H, CH_2 protons of alkyl chains), 0.92 (br, 12H, H_5), 0.85 (br, 36H, methyl protons of alkyl chains.).

^{13}C NMR (176 MHz, $CDCl_3$): δ_C = [192.6, 192.5 (C_{32})], [171.3, 171.2, 171.2 (rotamers, C_{67})] [170.9, 170.9 (rotamers, C_1)], [166.4, 166.3, 166.2, 166.1 ($C_{6,21,24,27}$)], [166.6, 166.5, 166.5, 165.4, 165.3, 165.2, 165.2, 165.0, 165.0, 164.8, 164.8 ($C_{7,11,20,22,23,25,26,28}$)], [163.0, 162.9 ($C_{48}$)], [159.8, 159.8 ($C_{43}$)], 152.1 ($C_{16}$), [143.9, 143.8 (rotamers, C_{65})], [141.4, 141.4 (C_{51})], [141.3, 141.2, 141.2, 141.2 (C_{37})], [139.6, 139.5, 139.3 (C_{52})], [138.4, 138.3, 138.3 (rotamers, C_{58})], [137.1, 137.0 (rotamers, C_{62})], [136.6, 136.6 (C_{33})], [133.8, 133.8 (C_{36})], [130.1, 130.1 (rotamers, C_{60})], [129.6, 129.5 (C_{45})], [129.1, 129.1, 129.0 (C_{35})], [128.7, 128.7 (C_{34})], 128.5 (C_{38}), [128.4, 128.3, 128.3, 128.2 (rotamers, C_{59})], [128.0, 127.8, 127.7 (C_{50})], [127.7, 127.7, 127.6 (C_{46})], [122.2, 122.2, 122.1 (rotamers, C_{64})], [120.2, 120.1, 120.1 (rotamers, C_{61})], [120.0, 120.0, 120.0, 119.9 (rotamers, C_{63})], [117.2, 117.2 (C_{15})], 114.4 (C_{53}), 114.2 (C_{44}), 112.8 (C_{49}), [70.4, 70.4 (C_{47})], 65.5 (C_{57}), [58.8, 58.8, 58.7, 58.6 (C_{14})], 57.9 (C_{66}), 55.9 (C_3), [55.4, 55.4 (C_{42})], [51.0, 50.9 (C_{55})], [50.7, 50.7 (rotamers, $C_{2/68}$)], [50.6, 50.5 (rotamers, $C_{2/68}$)], 50.0 ($C_{39,40}$), [49.0, 48.9, 48.8 (C_{54})], [44.3, 44.3, 44.2, 44.1, 43.4, 43.3, 43.2, 43.1, 43.0, 42.2, 42.2, 42.1 ($C_{8,12,19,29}$ and carbons of piperazine rings)], [38.3, 38.3 (C_{56})], [38.1, 38.0 (C_{41})], [34.3, 34.3, 34.2 (C_{17})], [32.6, 32.4 (C_{13})], [32.1, 32.0, 31.9 (C_{18})], [30.8, 29.8, 29.1, 29.0, 29.0, 28.9 (CH_2 carbons of alkyl chains)], [28.2, 28.2 (C_4)], [26.0, 25.9, 25.9, 25.9, 25.8, 25.8, 25.2, 25.1, 25.1, 25.0 ($C_{9,10,30,31}$)], [24.0, 23.3 (CH_2 carbons of alkyl chains)], [20.6, 20.5 (C_5)], [14.3, 10.9 (methyl carbons of alkyl chains)].

HRMS (ES+): Calculated for $C_{223}H_{308}N_{60}O_{19}^{2+}$, 2065.2484; found 2065.2715.

FT-IR (ATR): ν_{max} 2956, 2928, 2854, 1755, 1699, 1611, 1578, 1530, 1482, 1433, 1370, 1341, 1315, 1252, 1180, 1101, 1071, 994, 910, 845, 807, 732, 651.

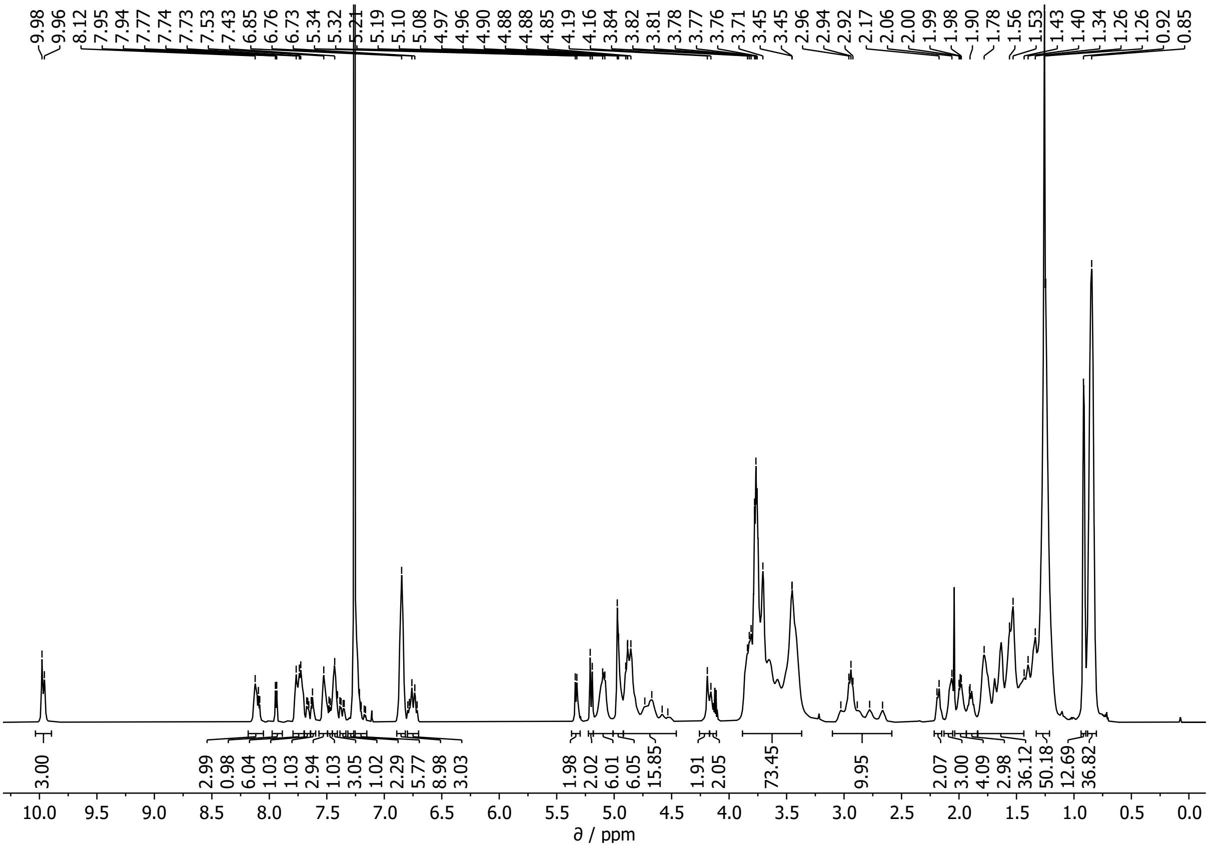

Figure S42 ^{1}H NMR spectrum (700 MHz, $CDCl_3$, 298 K) of **20**.

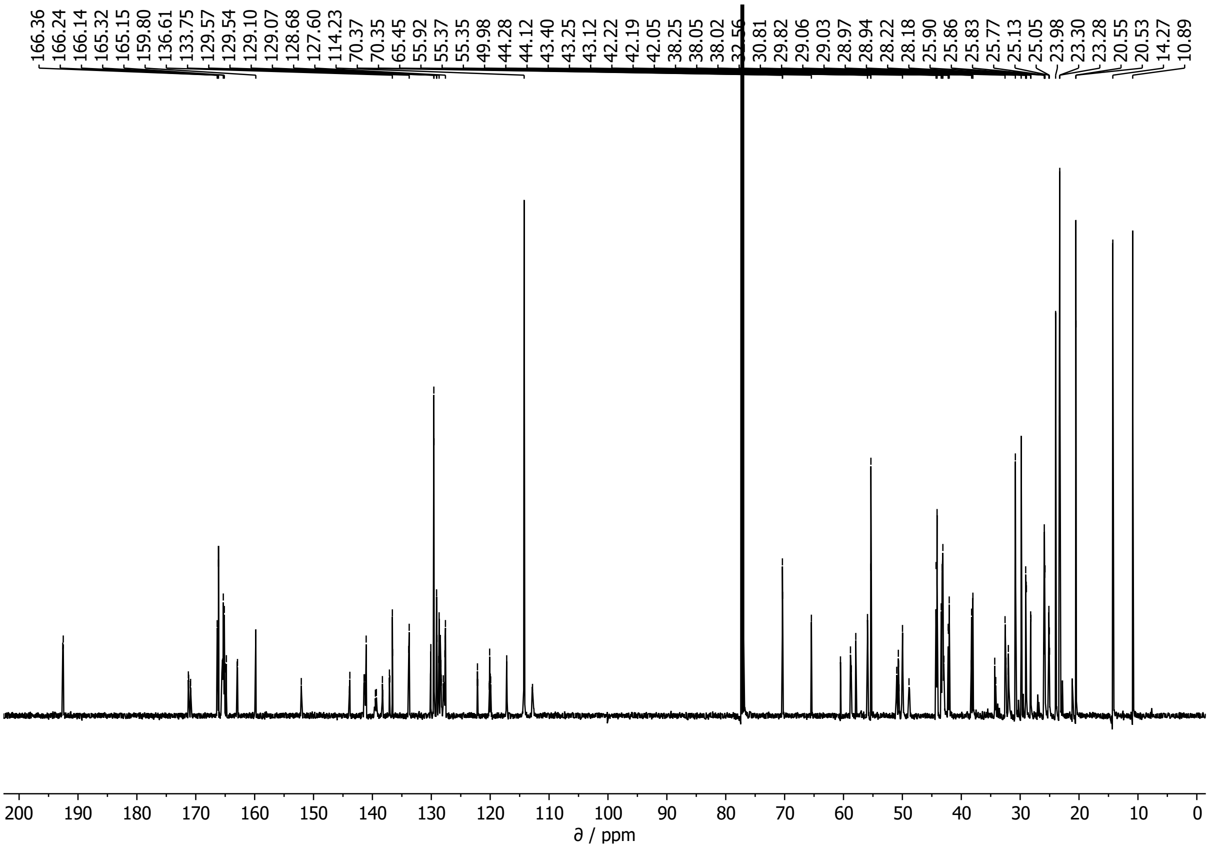

Figure S43 ^{13}C NMR spectrum (176 MHz, $CDCl_3$, 298 K) of **20**.

Synthesis of 21

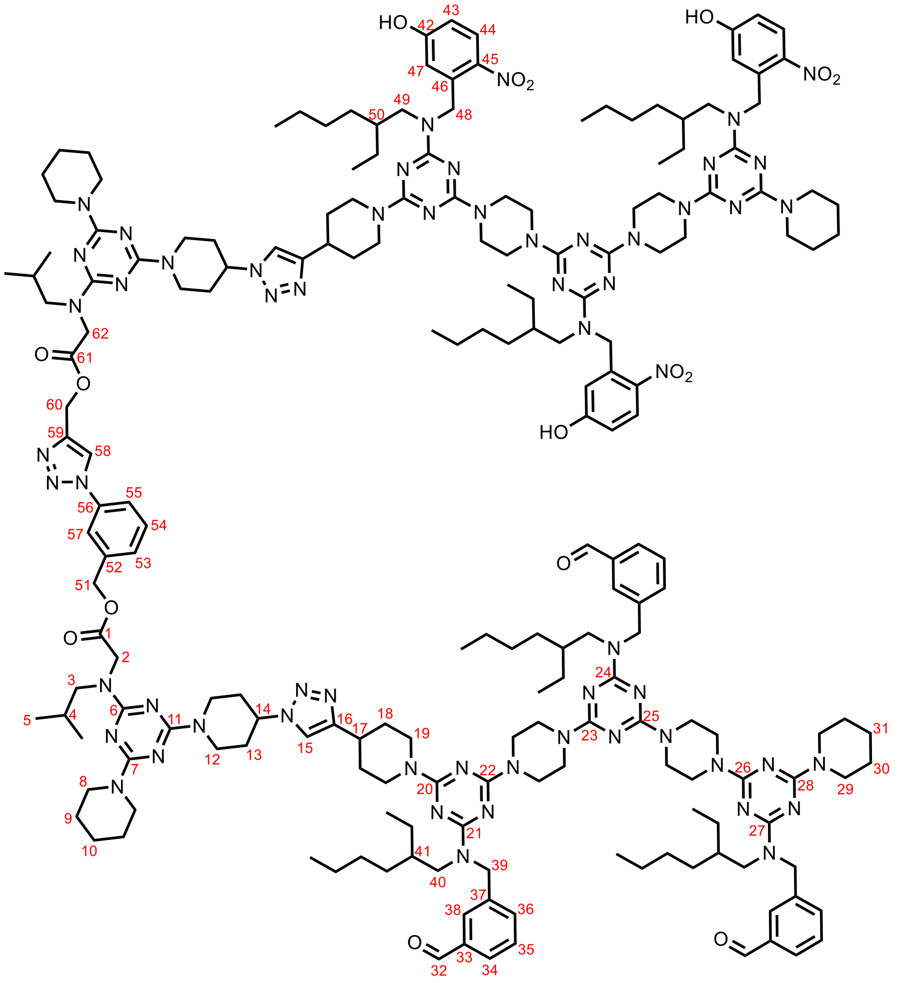

To a solution of **20** (50 mg, 0.012 mmol, 1.0 eq.) in DCM (2 mL), trifluoroacetic acid (2 mL) was added and the solution was stirred at room temperature for 5 minutes. The solvent was removed under reduced pressure and the residue was redissolved in ethyl acetate (10 mL). The organic phase was washed with water (10 mL) and the aqueous phase was extracted with ethyl acetate (10 mL). The combined organic phases were dried over magnesium sulphate and the solvent was removed under reduced pressure. The obtained residue was purified by silica flash chromatography (DCM / methanol (95:5)). **21** was obtained as a pale yellow oil (45 mg, 0.012 mmol, quantitative).

^{1}H NMR (500 MHz, $CDCl_3$): δ_H = 9.97 (br, 3H, H_{32}), 8.03 (br, 3H, H_{44}), 7.94 (s, 1H, H_{58}), 7.74 (br, 6H, $H_{34,38}$), 7.65 (br s, 1H, H_{57}), 7.60 (br, 1H, H_{55}), 7.51 (br, 3H, H_{36}), 7.47 (br, 1H, H_{54}), 7.43 (br, 3H, H_{35}), 7.35 (br, 1H, H_{53}), 7.28 (br, 2H, H_{15}), 6.68 (br, 6H, $H_{43,47}$), 5.31 (br, 2H, H_{60}), [5.20, 5.18 (rotamers, s, 2H, H_{51})], 5.09 (br, 6H, H_{48}), 4.95 – 4.43 (br, 16H, $H_{8,12,14,19,29,39}$ and protons of piperazine rings), 4.17 (s, 2H, H_2), 4.14 (s, 2H, H_{62}), 3.88 – 3.23

(br, 64H, $H_{3,8,12,19,29,40,49}$ and protons of piperazine rings), 3.07 – 2.62 (br, 10H, $H_{8,12,17,19,29}$ and protons of piperazine rings), 2.36 – 1.44 (br, 48H, $H_{9,10,13,18,30,21}$), 1.25 (br, 48H, CH_2 protons of alkyl chains), 0.90 (br, 12H, H_5), 0.84 (br, 36H, methyl protons of alkyl chains).

^{13}C NMR (126 MHz, $CDCl_3$): δ_C = [192.8, 192.6 (C_{32})], [171.4, 171.3 (rotamers, C_{61})], 171.1 (C_1), [166.3, 166.2, 166.1 ($C_{6,21,24,27}$)], [165.5, 165.3, 165.1, 165.0, 164.8 ($C_{7,11,20,22,23,25,26,28}$)], 162.3 ($C_{42}$), 151.9 ($C_{16}$), 143.8 ($C_{59}$), [141.2, 141.1, 141.0 (C_{37})], 140.7 (C_{45}), 139.8 (C_{46}), [138.4, 138.3 (rotamers, C_{52})], [137.0, 136.9, 136.8 (rotamers, C_{56})], 136.6 (C_{33}), [133.9, 133.8 (C_{36})], 130.2 (C_{54}), 129.1 (C_{35}), [128.8, 128.6, 128.6, 128.4 ($C_{34,38,53}$)], [128.3, 128.2 (C_{44})], [122.4, 122.3 (rotamers, C_{58}), 120.2 (C_{55}), 120.0 (C_{57}), 117.7 (C_{15}), [115.1, 115.0 (C_{47})], [114.1, 113.9 (C_{43})], [65.5, 65.1 (rotamers, C_{51})], [59.0, 58.9 (C_{14})], 57.7 (C_{60}), 56.0 (C_3), 55.4 (C_{49}), 51.0 ($C_{2,62}$), 50.7 ($C_{39,40}$), 50.0 (C_{48}), [48.9, 44.3, 44.2, 44.1, 43.2, 42.2, 42.0 ($C_{8,12,19,29}$ and carbons of piperazine rings)], 38.2 (C_{50}), 38.0 (C_{41}), [34.0, 33.8, 33.5 (C_{17})], [32.5, 32.4, 32.3, 32.1, 31.9, 31.8 ($C_{13,18}$)], [30.8, 30.3, 29.8, 29.6, 29.6, 29.5, 29.4, 29.4, 29.2, 29.0, 29.0 (CH_2 carbons of alkyl chains)], 28.2 (C_4), [26.8, 25.9, 25.9, 25.8, 25.1, 25.0 ($C_{9,10,30,31}$)], [24.0, 23.3, 22.8 (CH_2 carbons of alkyl chains)], 20.5 (C_5), [14.3, 10.9 (methyl carbons of alkyl chains)].

HRMS (ES+): Calculated for $C_{199}H_{284}N_{60}O_{16}^{2+}$, 1885.1627; found 1885.1542.

FT-IR (ATR): ν_{max} 3066, 2954, 2926, 2900 (br), 2853, 1750, 1700, 1525, 1478, 1432, 1369, 1331, 1303, 1253, 1184, 1100, 1070, 995, 910, 854, 807, 755, 733, 699, 650.

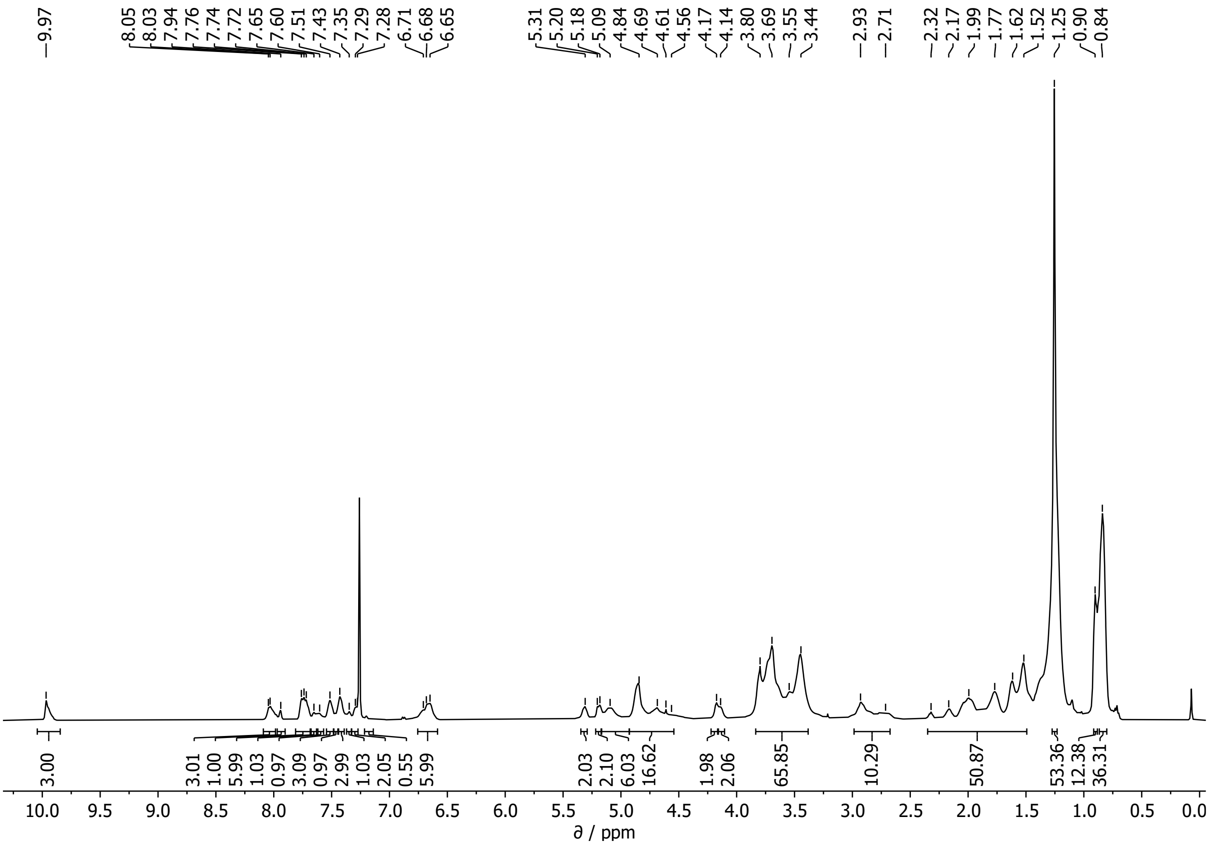

Figure S44 ^{1}H NMR spectrum (500 MHz, $CDCl_3$, 298 K) of **21**.

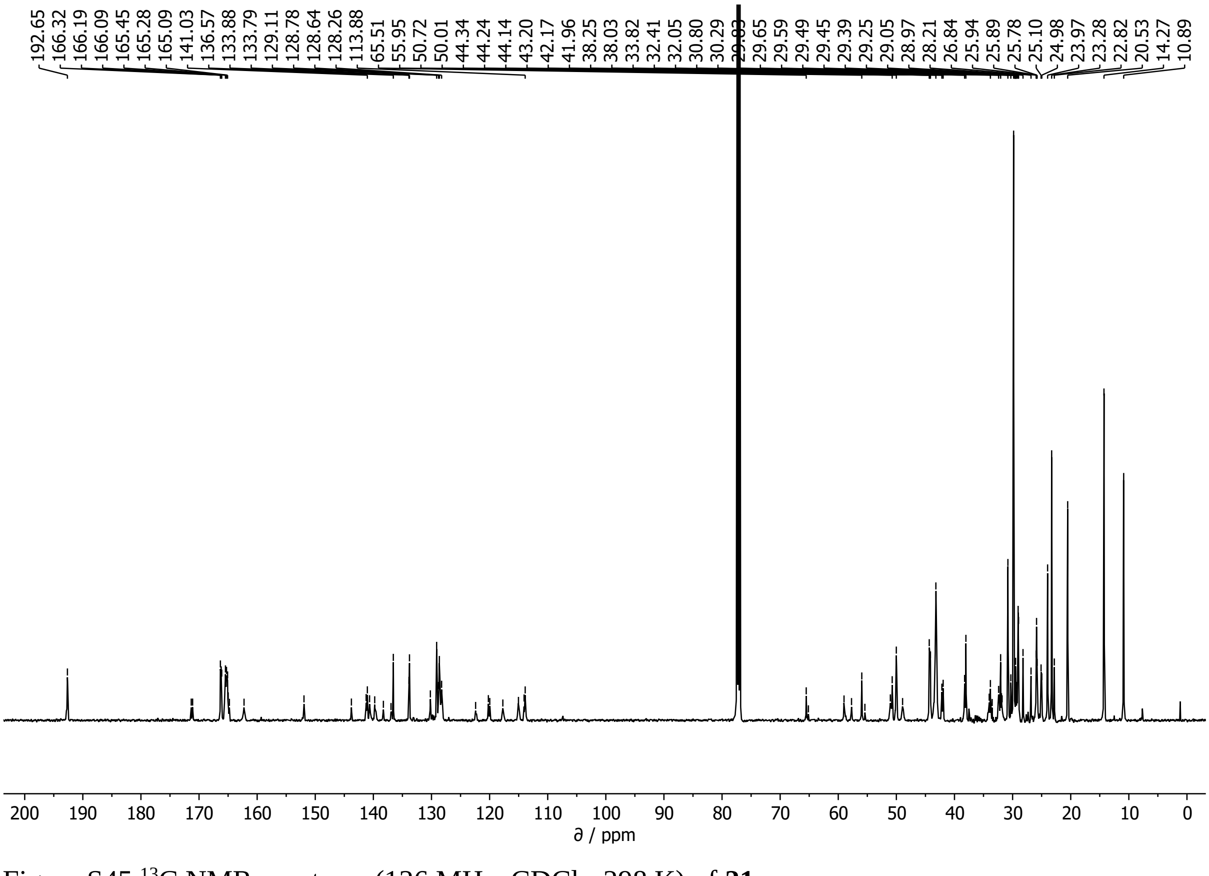

Figure S45 ^{13}C NMR spectrum (126 MHz, $CDCl_3$, 298 K) of **21**.

3. Determination of the relative imine stabilities

3,5-di-*tert*-Butylbenzaldehyde (10 mM) was mixed with benzylamine (15 mM) and **2** (15 mM) in $CDCl_3$. The mixture was left for 8 days before the equilibrium between the two imine species was determined by ^1H NMR (Figure S46 and Figure S47).

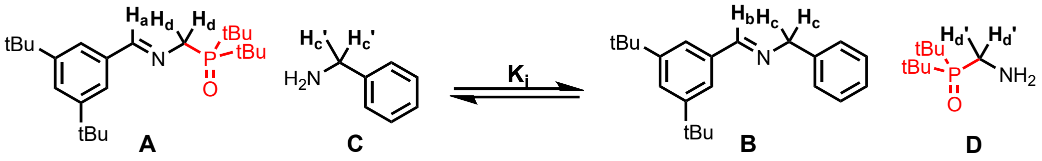

Figure S46 The equilibrium established by the mixing of 3,5-di-*tert*-butylbenzaldehyde with benzylamine (**C**) and **2** (**D**) to yield imines **A** and **B**. The protons used to determine the equilibrium constant (K_i) are labelled.

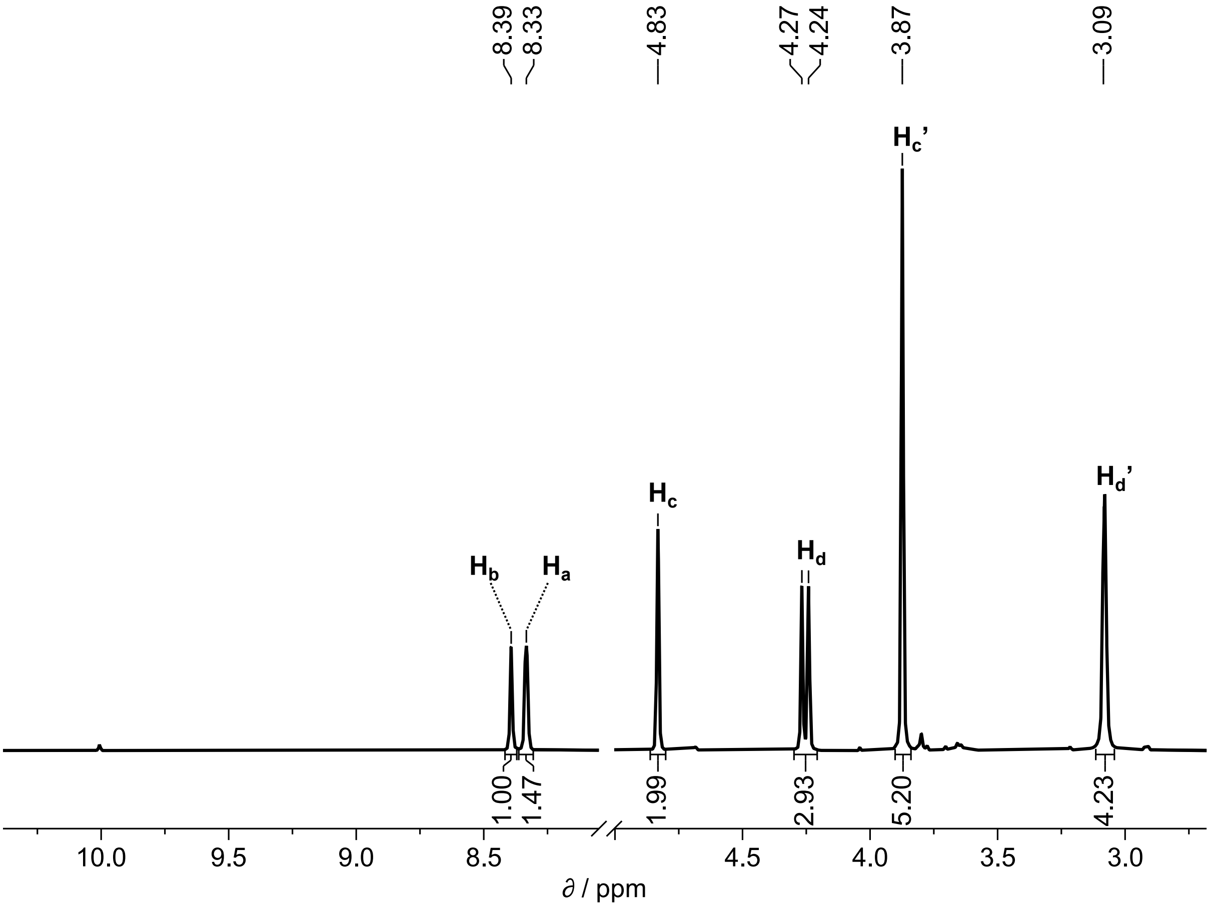

Figure S47 ^1H NMR spectrum (400 MHz, $CDCl_3$, 298 K) of the equilibrated imine mixture. The protons are labelled according to Figure S3.1.

The equilibrium constant is defined as:

$$K_i = \frac{[\boldsymbol{B}][\boldsymbol{D}]}{[\boldsymbol{A}][\boldsymbol{C}]}$$

so can be expressed as:

$$K_i = \frac{[I_b][I_{d'}]}{[I_a][I_{c'}]}$$

where I_x is the integral of proton x. Evaluating the equation gives a value for K_i of 0.55.

To determine the equilibrium proportions of each imine, at an arbitrary aldehyde concentration x, when two equivalents of each amine are used, it is known that:

$$[\boldsymbol{A}] + [\boldsymbol{B}] = x$$

$$[\boldsymbol{A}] + [\boldsymbol{D}] = 2x$$

$$[\boldsymbol{B}] + [\boldsymbol{C}] = 2x$$

Therefore:

$$K_i = \frac{[\boldsymbol{B}]([\boldsymbol{B}]+x)}{(x-[\boldsymbol{B}])(2x-[\boldsymbol{B}])}.$$

Solving the quadratic equation allows the percentage of each imine to be determined:

$$Percentage\ \boldsymbol{A} = 1 - \alpha$$

$$Percentage\ \boldsymbol{B} = \alpha$$

where:

$$\alpha = \frac{3K_i+1-\sqrt{(3K_i+1)^2-8(K_i-1)K_i}}{2(K_i-1)}.$$

For K_i = 0.55, the proportion of imine **A** is 61% and the proportion of imine **B** is 39%.

4. Procedure for the dynamic imine formation in DCM

Stock solutions of **2**, benzylamine, **11** and **18** were made up in DCM-d_2 for studies of the unlinked system. For the linked system a stock of **21** was made up. For the unlinked system, calculated amounts of **11** (1 eq.), **18** (1 eq.), **2** (6 eq.) and benzylamine (6 eq.) were mixed and diluted with a calculated volume of DCM-d_2 to give a concentration of **11** of 1.0 mM in 600 µL. For the linked system calculated amounts of **21** (1 eq.), **2** (6 eq.) and benzylamine (6 eq.) were mixed and diluted with a calculated volume of DCM-d_2 to give a concentration of **21** of 10 mM in 200 µL. The reactions were left for 1 day to equilibrate and ^{1}H NMR and ^{31}P NMR spectra were recorded. For the unlinked system, the mixture was diluted to give template concentrations of 0.75 mM, 0.50 mM, 0.25 mM and 0.10 mM. After each dilution, the solution was re-equilibrated for 1 day and a ^{1}H NMR spectrum was recorded. A ^{31}P NMR spectrum was recorded for the 0.1 mM solution. For the linked system, the 10 mM mixture was diluted to give template concentrations of 1.0 mM and 0.10 mM. After each dilution, the solution was re-equilibrated for 1 day then ^{1}H and ^{31}P NMR spectra were taken.

To determine the shift of the signals of each imine, the blank strand (10 mM) was mixed separately with **2** (60 mM) and benzylamine (60 mM) and diluted with calculated volumes of DCM-d_2 to make solutions of 200 µL. For the unlinked system **18** was used as the blank, for the linked system **21** was used as the blank. The solutions were equilibrated for 1 day then ^{1}H NMR spectra were recorded. The solution of **18** (1 eq.) with **2** (6 eq.) was diluted to give a blank concentration of 1.0 mM and a ^{31}P NMR spectrum was recorded.

To verify equilibration of the imine mixtures at 1.0 mM and 0.1 mM of template, ^{1}H NMR spectra of the mixtures with **21** were taken over time and the percentage yield of each imine calculated using the integrals of the signals of each imine. No change in mixtures was observed after 1 day (Figure S48).

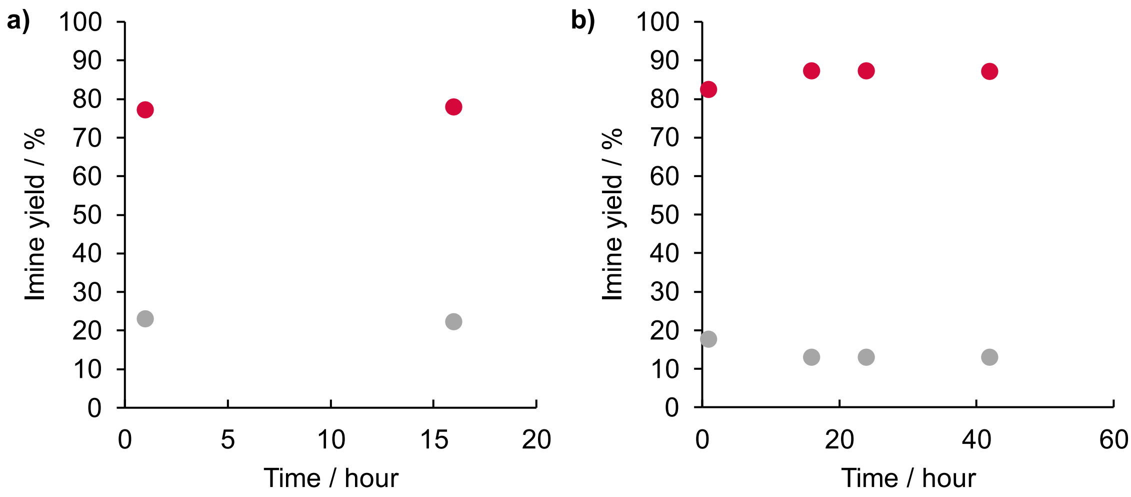

Figure S48 The total yield of the phosphine oxide imine (red) and benzyl imine (grey) over time for (a) the mixture of **21** (1.0 mM) with **2** (6.0 mM) and benzylamine (6.0 mM) in DCM-d_2 after dilution from a solution of **21** at 10 mM and (b) the mixture of **21** (0.1 mM) with **2** (0.6 mM) and benzylamine (0.6 mM) in DCM-d_2 after dilution from a solution of **21** at 1.0 mM.

To equilibrate the 0.1 mM solution in toluene, the DCM was removed by flushing under a stream of nitrogen. The residue was redissolved in toluene (1.0 mL) and re-equilibrated for 1 day.

5. Procedure for imine trapping and linker cleavage

To a solution of the imine mixture (0.1 mM of template) in toluene (1 mL), trichlorosilane (0.5 M) and DMF (0.5 M) were added and the solution stirred vigorously for 5 minutes. The solvent was removed by flushing under a stream of nitrogen and the residue was redissolved in ethyl acetate (0.5 mL). The solution was washed with saturated sodium hydrogen carbonate solution (0.5 mL) and the organic phase was collected. The solvent was flushed under a stream of nitrogen and the obtained residue was redissolved in THF (1 mL) and injected into the UPLC machine (6 μL).

For ester hydrolysis, the solvent was flushed and the residue was redissolved in THF (0.75 mL) and water (0.25 mL). Lithium hydroxide solution (3 drops, 1 M) was added and the solution stirred at room temperature overnight. The solution was injected into the UPLC machine (6 μL).

6. Study of the re-equilibration of the imine mixture in toluene

To determine the rate of re-equilibration of the imine library in toluene, a solution of **21** (3.0 mM), **2** (10.5 mM) and benzylamine (10.5 mM) in DCM (200 µL) was prepared and allowed to equilibrate overnight. The solution was flushed under nitrogen to remove the solvent and the residue redissolved in toluene (6.0 mL), giving a concentration of **21** of 0.1 mM. Aliquots of the solution (100 µL) were taken at time intervals, reduced, hydrolysed and analysed by UPLC as previously described. The yield of each copy strand over time was monitored and no change in the product distribution was observed after 1 day (Figure S49).

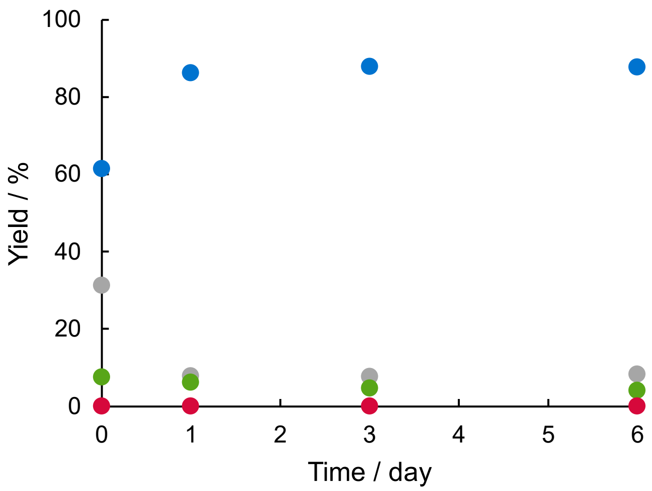

Figure S49 The yield of each copy strand as a function of the imine library equilibration time. The **A3** strand is shown in blue, **A2O** is shown in grey, **AO2** is shown in green and **O3** is shown in red.

7. Models of the linked and unlinked systems

The population of species according to each model was calculated using Musketeer software.[6] The software calculates a distribution of species based on the association constant for the formation of a 4-nitrophenol•phosphine oxide H-bond (K_h), the equilibrium constant for imine exchange between **2** and benzylamine (K_i), the effective molarity for any intramolecular equilibrium within a duplex (EM) and the degeneracy of the species. From the species distribution, the total percentage yield of phosphine oxide imine (P_{PO}) is calculated. For the unlinked system, the experimentally determined P_{PO} over the template concentration range 0.1 – 1.0 mM was fitted with known values of K_h and K_i leaving EM as the only variable, a value of 10 mM was obtained. The model used for the unlinked system is shown in Figure 6 and the model used for the linked system is shown in Figure S50. The model of the linked system assumes that H-bonds only form between directly opposite recognition units.

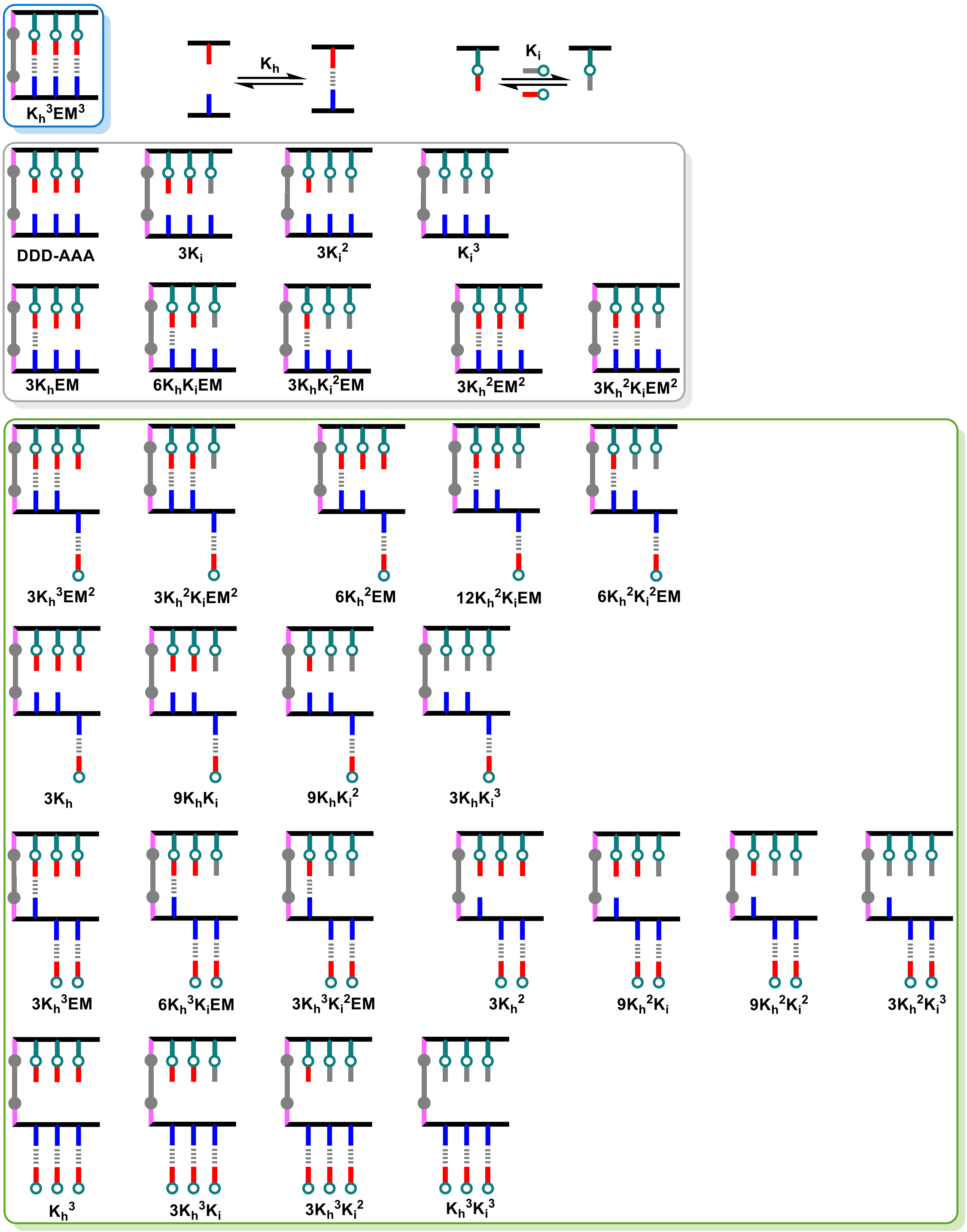

Figure S50 Cartoon representation of the species present in the imine library formed from a mixture of **21**, **2** and benzylamine. The global equilibrium constant relative to the free **DDD-AAA** species with no H-bonds is shown for each species. K_h is the association constant for formation of a 4-nitrophenol•phosphine oxide H-bond, K_i is the equilibrium constant for imine exchange between **2** and benzylamine, EM is the effective molarity for any intramolecular equilibrium within a duplex, and the numbers reflect the degeneracy of each species (only one of each sequence isomer is illustrated). Species are grouped as copy strands that are present as the fully assembled duplex (blue), duplexes with frayed H-bonds (grey), and duplexes that are partially denatured due to interaction with **2** (green).

8. DOSY NMR experiments

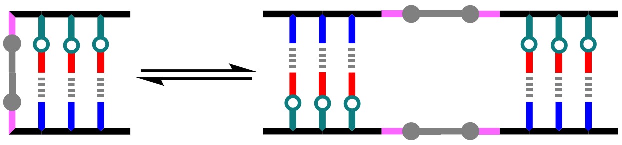

Figure S51 The possible competing dimerization of the tri-imine.

DOSY NMR experiments were performed to determine whether the fully assembled tri-imine could dimerise, giving a different mechanism for templating (Figure S51).

21 (7.5 mg, 0.002 mmol, 10 mM) and **2** (1.1 mg, 0.006 mmol, 30 mM) were mixed in CD_2Cl_2 (200 µL). The mixture was allowed to equilibrate for 1 day to yield the tri-imine (10 mM in CD_2Cl_2). DOSY NMR experiments were performed on the solution at 10 mM, 1.0 mM and 0.1 mM and compared to a DOSY NMR experiment of **21** at 0.1 mM. For **21**, the diffusion coefficient was obtained from the decaying signal at δ 9.96 ppm. For the tri-imine, the diffusion coefficient was obtained from the decaying signal at δ 8.17 ppm. Table S1 shows the DOSY diffusion coefficient for each solution and Figure S52 shows a plot of the normalised signal decay against the diffusing weighting, where the diffusion coefficient is given by the gradient.

Species	D / × 10^{-10} m^2s^{-1}
**21**	4.6
Tri-imine at 10 mM	2.3
Tri-imine at 1.0 mM	3.7
Tri-imine at 0.1 mM	4.1

Table S1 DOSY diffusion coefficient for each species.

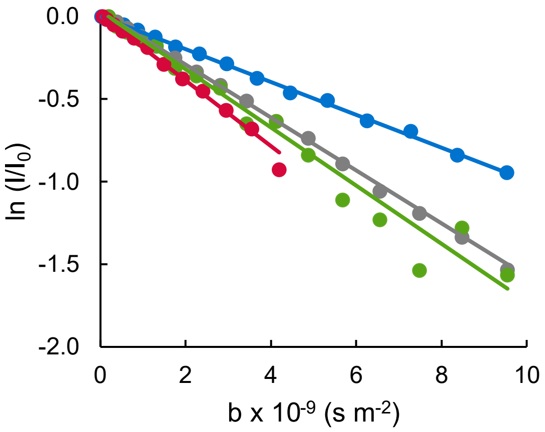

Figure S52 Plot of the natural logarithm of the normalised signal decay against the diffusing weighting for **21** (red) and the tri-imine at 10 mM (blue), 1.0 mM (grey) and 0.1 mM (green).

The similarities of the diffusion coefficients for the solutions at 1.0 mM and 0.1 mM to that of **21** indicate no dimerization whilst the larger diffusion coefficient at 10 mM indicates some dimerization.

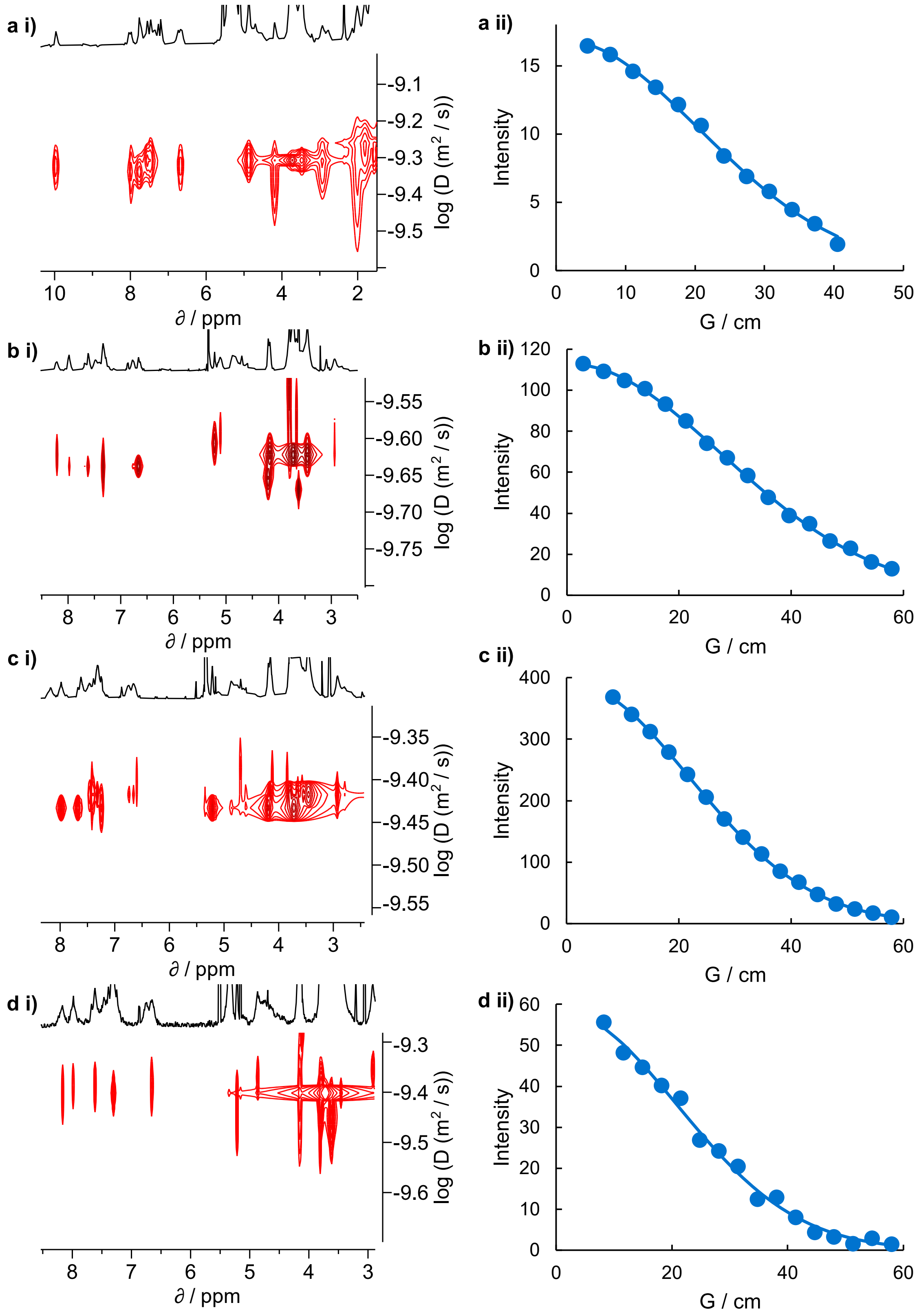

Figure S53 (i) ^{1}H-pseudo 2D-plots of DOSY (400 or 500 MHz, CD_2Cl_2, 298 K, Δ = 0.1 s, δ = 2 ms) and (ii) plots of signal intensity against the gradient strength, with fits to the mono-exponential Stejskal-Tanner function, for (a) **21** (0.1 mM) and solutions of the tri-imine at (b) 10 mM, (c) 1.0 mM and (d) 0.1 mM.

9. Analysis of the reduced and cleaved copy mixture

The extracted ion chromatogram of the 4^+ peaks of each possible copy strand was obtained and the area integrated on MassLynx software. The obtained area integrals were used to calculate the yield of each copy (Table S2).

Product	Extracted ion intensity peak area ($\times 10^7$ arbitrary units)	Yield (%)
**A3**	4.42	85
**A2O**	0.46	9
**AO2**	0.21	4
**O3**	0.13	2

Table S2 The extracted ion intensity peak area and estimated yield for each product.

The total yield of phosphine oxide imine before reduction is given as:

$$P_{PO} = \frac{3A_{A3}+2A_{A2O}+A_{AO2}}{3(A_{A3}+A_{A2O}+A_{AO2}+A_{O3})}$$

where A_x is the integrated area of the peak in the extracted ion chromatogram of species x. The obtained results give P_{PO} = 92%.

10. References

(1) Troselj, P.; Bolgar, P.; Ballester, P.; Hunter, C. A. High-Fidelity Sequence-Selective Duplex Formation by Recognition-Encoded Melamine Oligomers. *J. Am. Chem. Soc.* **2021**, *143* (23), 8669–8678. https://doi.org/10.1021/jacs.1c02275.

(2) Balduzzi, F.; Munasinghe, V.; Evans, O. N.; Lorusso Notaro Francesco, A.; Anderson, C. J.; Nigrelli, S.; Escobar, L.; Cabot, R.; Smith, J. T.; Hunter, C. A. Length and Sequence-Selective Polymer Synthesis Templated by a Combination of Covalent and Noncovalent Base-Pairing Interactions. *J. Am. Chem. Soc.* **2024**, *146* (47), 32837–32847. https://doi.org/10.1021/jacs.4c13452.

(3) Dhiman, M.; Cons, R.; Evans, O. N.; Smith, J. T.; Anderson, C. J.; Cabot, R.; Soloviev, D. O.; Hunter, C. A. Selective Duplex Formation in Mixed Sequence Libraries of Synthetic Polymers. *J. Am. Chem. Soc.* **2024**, *146* (13), 9326–9334. https://doi.org/10.1021/jacs.4c01381.

(4) Yanagisawa, T.; Kuratani, M.; Seki, E.; Hino, N.; Sakamoto, K.; Yokoyama, S. Structural Basis for Genetic-Code Expansion with Bulky Lysine Derivatives by an Engineered Pyrrolysyl-tRNA Synthetase. *Cell Chem. Biol.* **2019**, *26* (7), 936-949.e13. https://doi.org/10.1016/j.chembiol.2019.03.008.

(5) Umezawa, N.; Matsumoto, N.; Iwama, S.; Kato, N.; Higuchi, T. Facile Synthesis of Peptide–Porphyrin Conjugates: Towards Artificial Catalase. *Bioorg. Med. Chem.* **2010**, *18* (17), 6340–6350. https://doi.org/10.1016/j.bmc.2010.07.018.

(6) Soloviev, D. O.; Hunter, C. A. Musketeer: A Software Tool for the Analysis of Titration Data. *Chem. Sci.* **2024**, *15* (37), 15299–15310. https://doi.org/10.1039/D4SC03354J.
